# Roles of CatSper channels in the pathogenesis of asthenozoospermia and the therapeutic effects of acupuncture-like treatment on asthenozoospermia

**DOI:** 10.7150/thno.51869

**Published:** 2021-01-01

**Authors:** Zi-Run Jin, Dong Fang, Bo-Heng Liu, Jie Cai, Wen-Hao Tang, Hui Jiang, Guo-Gang Xing

**Affiliations:** 1 Neuroscience Research Institute, Peking University; Department of Neurobiology, School of Basic Medical Sciences, Peking University Health Science Center; Key Laboratory for Neuroscience, Ministry of Education of China & National Health Commission of China, Beijing 100191, China.; 2 The Second Affiliated Hospital of Xinxiang Medical University, Henan, Xinxiang 453002, China.; 3 Department of Urology, the Third Hospital, Peking University, Beijing 100191, China.

**Keywords:** idiopathic asthenozoospermia, male infertility, CatSper, acupuncture, sperm motility

## Abstract

**Rationale:** Idiopathic asthenozoospermia (iAZS) is one of the major causes of male infertility and has no effective therapeutic treatment. Understanding the potential mechanisms that cause it may be helpful in seeking novel targets and treatment strategies for overcoming the problem of low sperm motility in iAZS individuals.

**Methods:** Computer-assisted semen analysis (CASA) was utilized to assess the sperm motility. RT-qPCR, Western blot, immunofluorescence staining, and calcium imaging analysis were performed to examine the expression and function of CatSper channels. Hyperactivation and acrosome reaction were used to evaluate the functional characteristics of epididymal sperm. *In vivo* fertility assay was applied to determine the fertility of rats. CatSper1 knockdown and overexpression experiments were performed to confirm the roles of CatSper channels in the pathogenesis of iAZS and the therapeutic effects of electroacupuncture (EA) treatment on AZS model rats.

**Results:** Here, we reported a functional down-regulation of CatSper channel from CatSper1 to CatSper 4 in the sperm of both iAZS patients and ornidazole (ORN)-induced AZS model rats, and an impaired sperm function characterized by a reduction of protein tyrosine phosphorylation, hyperactivation, and acrosome reaction in the epididymal sperm of AZS rats. Knockdown of CatSper1 in the testis tissues is sufficient to induce AZS in normal rats, and this action was validated by the reversal effects of CatSper1 overexpression. Transcutaneous electrical acupoint stimulation (TEAS) and electroacupuncture (EA) at 2 Hz frequency improve the sperm motility via enhancing the functional expression of CatSper channels in the sperm. Gene silencing *CatSper1* in the sperm abolishes the therapeutic effects of 2 Hz-EA treatment on AZS rats.

**Conclusions:** We conclude that a functional down-regulation of CatSper channel in the sperm may be a contributor or a downstream indicator for a portion of AZS, especially iAZS, while 2 Hz-TEAS or EA treatment has a therapeutic effect on iAZS through inducing the functional up-regulation of CatSper channels in the sperm. This study provides a novel mechanism for the pathogenesis of some AZS especially iAZS, and presents a potential therapeutic target of CatSper for iAZS treatment. Acupuncture treatment like TEAS may be used as a promising complementary and alternative medicine (CAM) therapy for male infertility caused by iAZS in clinical practice.

## Introduction

Infertility is a worldwide problem affecting about 15% of couples trying to conceive. Asthenozoospermia (AZS), which characterized by reduced forward sperm motility, is a common cause of male infertility. Nevertheless, a number of causes can lead to AZS, such as varicocele, endocrine abnormality, environmental factors, inflammation, drug injury, and some basic diseases, *etc*. However, no clear causes have been diagnosed in some cases using routine clinical examinations, and these cases have been categorized as idiopathic AZS (iAZS) 1. The mechanisms underlying the pathogenesis of iAZS are largely unknown.

A sperm-specific calcium channel, CatSper (cation channel of sperm), which allows calcium influx into sperm, plays a vital role in the regulation of sperm motility, hyperactivity and male fertility 2-4. Male mice lacking CatSper genes 5-8 or men with genetic mutation of CatSper channels 9-13 are completely infertile due to lack of sperm hyperactivation. The mammalian CatSper channel is a sophisticated complex that comprises at least ten different proteins, including four pore-forming α subunits (CatSper1-CatSper4) and six accessory subunits (CatSper β, γ, δ, ε, ζ, and EFCAB9) 3, 14. Among them, all the four α CatSper genes are proved necessary for sperm hyperactivated motility and male fertility 7. It is now considered that CatSper1 and CatSper2 are required for the beat of sperm flagella and hyperactivated sperm motility 5, 8, 15, 16, while CatSper3 and CatSper4 also participate in the acrosome reaction and egg coat penetration 6, 17, 18. Targeted disruption of CatSper1-CatSper4 genes in mouse leads to complete loss of CatSper current (I_CatSper_) and identical phenotype of male infertility, suggesting that all the four α subunits are essential for a functional CatSper channel 6, 7, 15. CatSper genes are exclusively expressed in the testis during spermatogenesis, and a reduction of CatSper gene expression was found among patients who lack sperm motility 19. In addition, the gene expression of CatSper1, 2, 3, and 4 was lower in infertile men with AZS as compared with fertile individuals with normal sperm parameters 20, and the mRNA levels of CatSper2 and CatSper3 in the low-motile spermatozoa were lower than those in the high-motile fraction of human ejaculated spermatozoa 21. Consistently, a decreased expression of CatSper1 and CatSper2 protein was observed in the sperm of AZS patients 22, 23. In a previous study, we found that the expressions of both CatSper1 mRNA and protein were decreased in the epididymal spermatozoa of AZS model rats, whereas up-regulation of CatSper1 by Sheng-Jing-San, a traditional Chinese medicine (TCM) recipe treatment improves the sperm motility of AZS rats 24. However, whether and how the decreased CatSper channels contribute to the pathogenesis of iAZS remain unclear.

Moreover, the ideal therapies for iAZS have not been established 25. Originating from TCM, acupuncture-like therapies like electroacupuncture (EA) and transcutaneous electrical acupoint stimulation (TEAS), have been widely used as an adjunctive treatment for many kinds of illnesses including infertility 26-28. Although the effect and safety of acupuncture for the treatment of male infertile patients with iAZS have been demonstrated in several studies 29-32, the molecular mechanisms underlying the acupuncture-induced improvement of sperm quality especially sperm motility are not clear yet.

In this study, we first investigated the roles and mechanisms of CatSper channels in the pathogenesis of iAZS. Then we investigated whether acupuncture-like treatment exerts its actions on iAZS patients and AZS model rats via CatSper channels.

## Results

### Decreases in expression and function of CatSper channels in the sperm of iAZS patients

**Figure 1:**
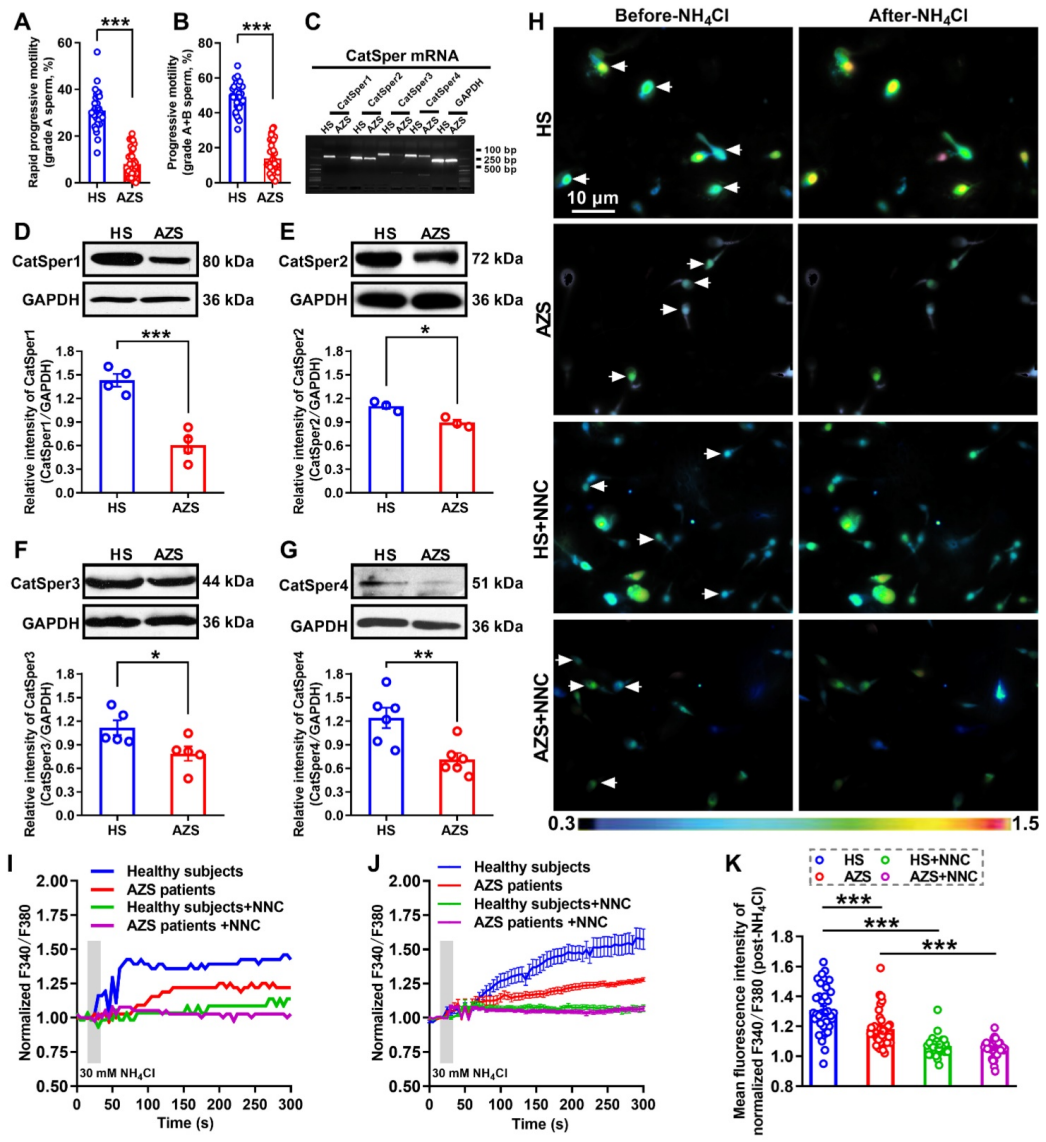
** Reduction of sperm motility and functional CatSper channels expression in the spermatozoa of idiopathic asthenozoospermic (iAZS) patients.** (A-B) Sperm motility including rapid progressive motility (grade A) sperm (A) and progressive motility (grade A+B) sperm (B) in AZS patients (n = 35) and healthy subjects (HS) (n = 35). (C-G) CatSper mRNA (C) and protein (from CatSper1 to CatSper4) (D-G) expression in the spermatozoa of AZS patients and HS controls (n = 3-6 tested subjects per group). (H) Representative fluorescence images from Fura-2 loaded human sperm before and after 30 mM NH_4_Cl treatment in different groups as indicated. Arrows indicate the [Ca^2+^]_i_ fluorescent signals of the sperm in response to NH_4_Cl treatment. Scale bar = 10 μm. (I) Representative single sperm fluorescence traces. (J) Changes in normalized [Ca^2+^]_i_ fluorescent signals of all tested sperm. (K) Summary plot of normalized [Ca^2+^]_i_ fluorescent signals of all tested sperm in response to NH_4_Cl treatment (n = 32-39 spermatozoa from 4 to 6 tested subjects per group). All data are presented as mean ± SEM.^ *^P < 0.05, ^**^P < 0.01, ^***^P < 0.001. Unpaired *t* test for (A)-(B), and (D)-(G); one-way ANOVA with Sidak's *post-hoc* test for (K).

To determine whether decreased CatSper channels in the sperm is responsible for the pathogenesis of iAZS, we first examined the alterations of CatSper abundance, at both protein and mRNA levels, in the sperm of idiopathic AZS (iAZS) patients. Validation of patients with iAZS was performed by computer-assisted semen analysis (CASA), and a reduction of sperm motility including rapid progressive motility (grade A) and progressive motility (grade A+B) sperm was found in iAZS patients compared with healthy subjects (HS) (Figure 1A-B). The mRNA expression of all the four pore-forming alpha subunits of CatSper channels including CatSper1, 2, 3, and 4 was observed in the sperm of iAZS patients and HS controls by reverse transcription-PCR (Figure 1C). Then, using Western blotting assay, we found a substantial decrease in the abundance of CatSper protein from CatSper1 to CatSper4 in the sperm of iAZS patients compared with HS controls (Figure 1D-G). Consistently, using a Ca^2+^ sensitive fluorescent probe to examine the NH_4_Cl-evoked, CatSper-mediated Ca^2+^ influx into sperm, we observed a significant reduction of NH_4_Cl-induced [Ca^2+^]_i_ fluorescent signals in the sperm of iAZS patients in contrast to HS controls (Figure 1H-K, and Video S1-1). As a summarization, a consistent decrease in NH_4_Cl-induced [Ca^2+^]_i_ fluorescent signals was observed in single spermatozoa (I) as well as in all tested sperm (J) and the mean fluorescence intensity of post-NH_4_Cl in all tested sperm (K) from iAZS patients. Moreover, we found that NNC 55-0396, a CatSper channels inhibitor substantially blocked the NH_4_Cl-induced [Ca^2+^]_i_ fluorescent signals in each group (Figure 1H-K, and Video S1-2, S1-3), indicating that the NH_4_Cl-evoked Ca^2+^ influx into sperm is mainly mediated by CatSper channels. These results suggest that the decreased CatSper channels in the sperm are involved in the pathogenesis of iAZS in patients.

### Improvement of sperm motility and functional up-regulation of CatSper channels in the sperm of iAZS patients by 2 Hz-TEAS treatment

**Figure 2:**
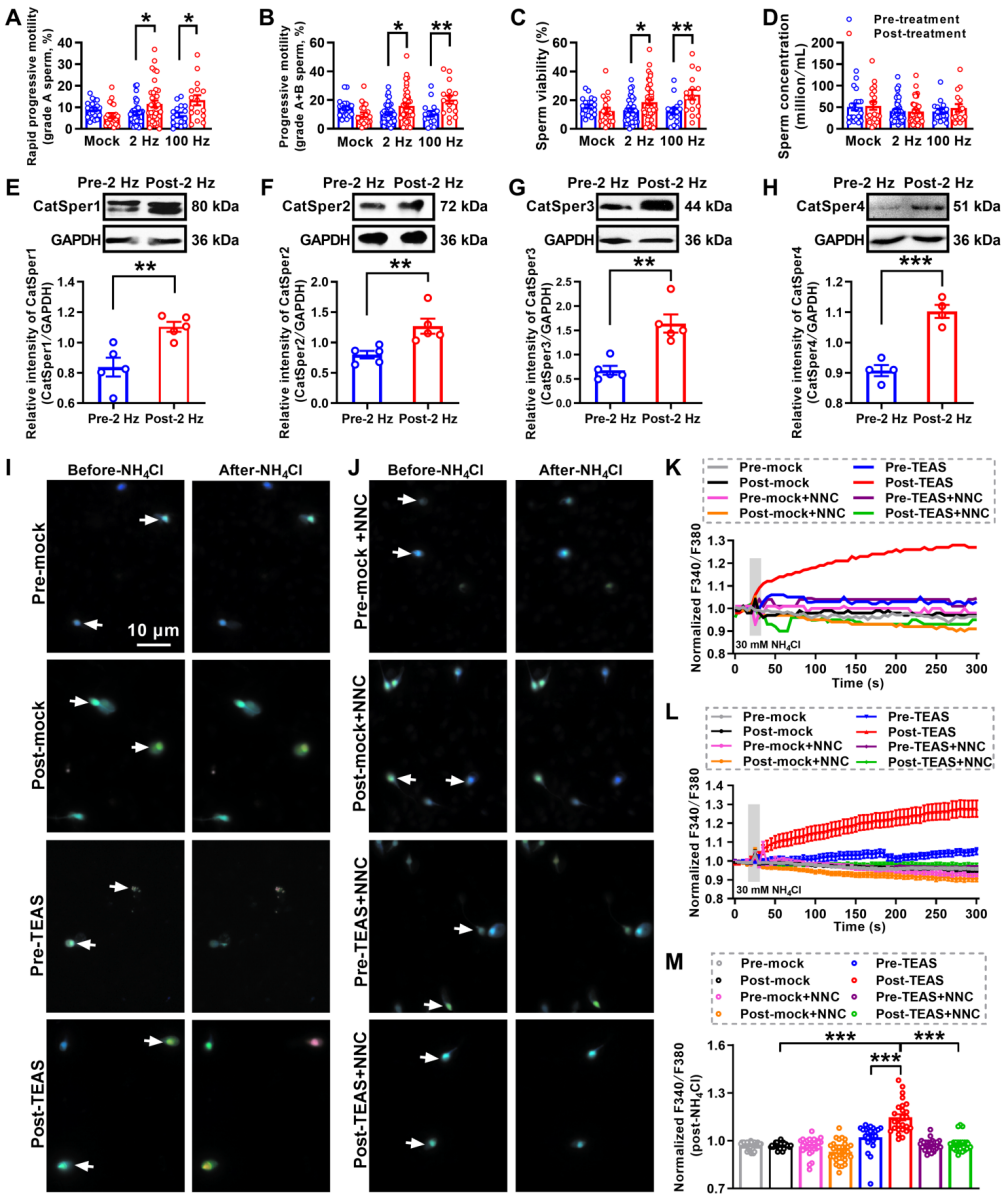
** Improved sperm motility and enhanced functional CatSper channels expression in the spermatozoa of idiopathic asthenozoospermic (iAZS) patients with 2 Hz-TEAS treatment.** (A-D) Improvement of sperm motility and sperm viability by 2 Hz- or 100 Hz-TEAS treatment to AZS patients (n = 16-41 tested subjects per group). (E-H) Elevated CatSper protein abundance (from CatSper1 to CatSper4) in the sperm of AZS patients treated with 2 Hz-TEAS treatment (n = 4-5 tested subjects per group). (I-J) Representative fluorescence images from Fura-2 loaded human sperm before and after administering 30 mM NH_4_Cl in different groups as indicated. Arrows indicate the [Ca^2+^]_i_ fluorescent signals of the sperm in response to NH_4_Cl treatment. Scale bar = 10 μm. (K) Representative single sperm fluorescence traces. (L) Changes in normalized [Ca^2+^]_i_ fluorescent signals of all tested sperm. (M) Summary plot of normalized [Ca^2+^]_i_ fluorescent signals of all tested sperm in response to NH_4_Cl treatment (n = 23-33 spermatozoa from three tested subjects per group). All data are presented as mean ± SEM.^ *^P < 0.05, ^**^P < 0.01, ^***^P < 0.001. One-way ANOVA with Sidak's *post-hoc* test for (A)-(D), and (M); Unpaired *t* test for (E)-(H). See also Figure S1.

Up to now, the ideal therapies for iAZS have not been established. We and others have found that acupuncture-like therapies like EA or TEAS treatment can improve the sperm motility in AZS model rats or patients 30, 32, 33. To further determine whether the increased CatSper channels in the sperm are responsible for the underlying mechanisms of the acupuncture-mediated improvement of sperm quality especially sperm motility in iAZS patients, we first examined the alterations of sperm quality in TEAS treated-iAZS patients. Using CASA technique, we found a significant improvement of sperm motility (increased grade A sperm and grade A+B sperm) and sperm viability both in 2 Hz- and 100 Hz-TEAS treated-iAZS patients (Figure 2A-D). Notwithstanding the effective rate of 100 Hz-TEAS treatment (93.75%) was even higher than 2 Hz-TEAS treatment (65.85%) to iAZS patients, no significant alteration was observed on CatSper1 and CatSper3 protein abundance in the sperm of 100 Hz-TEAS treated-iAZS patients (Figure S1). In contrast, a significant increase in the abundance of CatSper protein (from CatSper1 to CatSper4) in the sperm was found in 2 Hz-TEAS treated-iAZS patients (Figure 2E-H). Therefore, we examined the effects of 2 Hz-TEAS treatment on the CatSper-mediated Ca^2+^ influx into sperm of iAZS patients. Strikingly, we found a prominent increase in NH_4_Cl-evoked [Ca^2+^]_i_ fluorescent signals in the sperm of iAZS patients after 2 Hz-TEAS treatment (Figure 2I-J, and Video S2-1, S2-2). As a summarization, a consistent increase in NH_4_Cl-evoked [Ca^2+^]_i_ fluorescent signals was seen in single spermatozoa (K) as well as in all tested sperm (L) and the mean fluorescence intensity of post-NH_4_Cl in all tested sperm (M) from iAZS patients. Moreover, we found that the CatSper channel inhibitor NNC 55-0396 (NNC) blocked the NH_4_Cl-evoked [Ca^2+^]_i_ fluorescent signals in each group (Figure 2I-M, and Video S2-3 to S2-6), indicating that the NH_4_Cl-evoked Ca^2+^ influx into sperm is mainly mediated by CatSper channels. Altogether, these results suggest that the functional up-regulation of CatSper channels is involved in the potential mechanisms of 2 Hz TEAS-induced improvement of sperm motility in iAZS patients.

### Decreases in expression and function of CatSper channels in the epididymal sperm and testis tissues of AZS model rats

To further clarify the underlying mechanism by which how the decreased CatSper channels in the sperm contributes to the pathogenesis of AZS in animals, we first developed a rat model of AZS by intragastric administration of ornidazole (ORN) as described elsewhere 34. Here, we used ornidazole rather than cyclophosphamide to induce the rat model of AZS was due to the reason that cyclophosphamide exists a serious general toxicity and may also induce a decreased sperm concentration, as observed in our previous study 24. Therefore, the cyclophosphamide-induced rat model is not a pure AZS but oligoasthenozoospermia animal model. In contrast, ornidazole has the advantage of fewer side-effects and it acts rapidly and reversibly to decrease the sperm motility 35, and the ornidazole-induced AZS is proposed by its' inhibition on sperm energy metabolism that is essential for spermatozoa motility and maturation 36. Although there seems to be differences in fertility between the iAZS patients and the ornidazole-induced AZS rat model (*e.g.*, the former is infertility and the latter is subfertility), the ornidazole-induced AZS in rats is still a feasible AZS animal model to be used widely in basic researches 37, 38.

**Figure 3:**
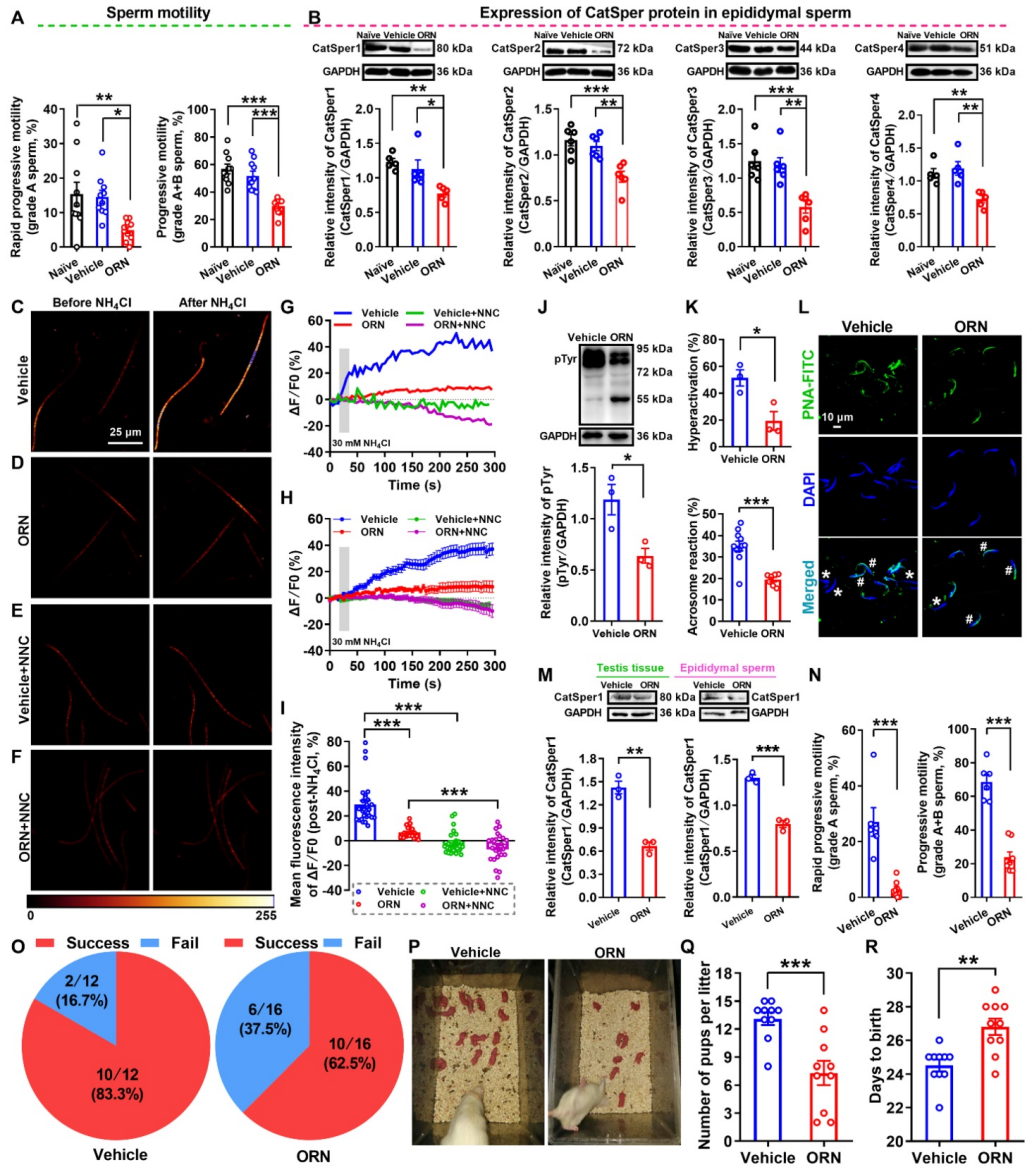
** Reduced sperm motility and functional CatSper channels expression in the spermatozoa, and impaired fertility in asthenozoospermic (AZS) model rats.** (A) Sperm motility in epididymal sperm of naïve rats, vehicle-treated rats, and ornidazole (ORN)-induced AZS rats (n = 10 rats per group). (B) CatSper protein (from CatSper1 to CatSper4) abundance (n = 5-6 rats per group). (C-F) Representative fluorescence images from Fluo-4 loaded sperm before and after 30 mM NH_4_Cl treatment in different groups as indicated. Scale bar = 25μm. (G) Representative single sperm fluorescence traces. (H) Changes in normalized [Ca^2+^]_i_ fluorescent signals of all tested sperm. (I) Summary plot of normalized [Ca^2+^]_i_ fluorescent signals of all tested sperm in response to NH_4_Cl treatment (n = 26-29 spermatozoa from 4-6 rats per group). (J-L) Protein tyrosine phosphorylation (pTyr) (J), hyperactivation (K), and acrosome reaction (AR) (K-L) in the epididymal sperm of vehicle- and ORN-treated rats (n = 3 rats per group). (L) Shown are representative images of sperm acrosome reaction of vehicle- and ORN-treated rats. Asterisk and pound sign indicate the sperm that has acrosome reaction (acrosome disappeared) and has no acrosome reaction (acrosome existed), respectively. Scale bar = 10 μm. (M-R) *In vivo* fertility assay of vehicle- and ORN-treated rats (n = 3-10 male rats per group). (M, N) Validation of the decreased CatSper1 protein (M) and sperm motility (N) in the ORN-treated male rats that to mate with the normal female rats. (O) The pregnancy rate of female rats that were mated with the ORN-induced AZS rats. (P-Q) Representative and a summary plot for number of pups per litter. (R) Days to birth of the pup. All data are presented as mean ± SEM.^ *^P < 0.05, ^**^P < 0.01, ^***^P < 0.001. One-way ANOVA with Sidak's *post-hoc* test for (A)-(B), and (I); Unpaired *t* test for (J)-(N) and (Q)-(R). See also Figures S2-S4.

Validation of animals with AZS was performed by CASA, and a reduction of sperm motility including grade A and grade A+B sperm was found in ORN-treated rats compared with naïve and vehicle controls (Figure 3A and Video S3-1). Other parameters of sperm motility including straight-line velocity (VSL), curve-line velocity (VCL), average path velocity (VAP), the amplitude of lateral head displacement (ALH), linearity (LIN) and straightness (STR), as well as sperm viability were consistently decreased in ORN-treated rats, whereas no significant difference in the percentage of sperm with abnormal morphology and the sperm concentration was found among naïve, vehicle, and ORN-treated rats (Figure S2A-J, and Video S3-1). These data indicated that intragastric administration of ornidazole to rats induced a reliable AZS animal model without other abnormal phenotypes of sperm, such as teratozoospermia or oligozoospermia. In addition, using immunofluorescence staining and Western blotting, we observed a substantial decrease in the abundance of CatSper protein (from CatSper1 to CatSper4) in the epididymal sperm of ORN-treated rats (Figure S2K-O, and Figure 3B). Similarly, a consistent decrease in the abundance of both CatSper mRNA and protein (CatSper1-CatSper4) was also observed in the testis tissues of ORN-treated rats (Figure S3).

In line with the decreased abundance of CatSper channels in the epididymal sperm and testis tissues of ORN-treated rats, we indeed found a reduction of NH_4_Cl-evoked Ca^2+^ fluorescent signals in the sperm of ORN-treated rats in contrast to vehicle controls (Figure 3C-I, and Video S3-2).

As a summarization, a consistent decrease in NH_4_Cl-evoked [Ca^2+^]_i_ fluorescent signals was seen in single spermatozoa (G) as well as in all tested sperm (H) and the mean fluorescence intensity of post-NH_4_Cl in all tested sperm (I) from ORN-treated rats. Also, we found that the CatSper inhibitor NNC 55-0396 (NNC) blocked all of the NH_4_Cl-evoked [Ca^2+^]_i_ fluorescent signals in each group (Figure 3E-I, and Video S3-3, S3-4), indicating that these NH_4_Cl-evoked [Ca^2+^]_i_ fluorescent signals were mainly mediated via CatSper channels. Moreover, accompanying with functional decrease of CatSper channels in the epididymal sperm of ORN-treated rats, we found an evident impairment of sperm function characterized by a reduction of protein tyrosine phosphorylation, hyperactivation, and acrosome reaction (staining with fluorescein isothiocyanate-conjugated peanut agglutinin, PNA-FITC) in the epididymal sperm of ORN-treated rats (Figure 3J-L). In addition, using the ORN-induced AZS male rats, which were validated by decreased CatSper1 protein (Figure 3M) and sperm motility (Figure 3N, and Figure S4), to mate with normal female rats, the *in vivo* fertility assay showed that both the pregnancy rate of female rats and the pup numbers per litter were decreased, whereas the days to birth of the pup was prolonged (Figure 3O-R). These data suggest that the decreased abundance of CatSper channels in the sperm results in the reduction of sperm motility, hyperactivation, and acrosome reaction, and subsequently leading to the impaired fertility of AZS male rats.

### Knockdown of CatSper1 in the testis tissues impairs the sperm motility and functional characteristics, and attenuates the fertility in normal male rats

**Figure 4:**
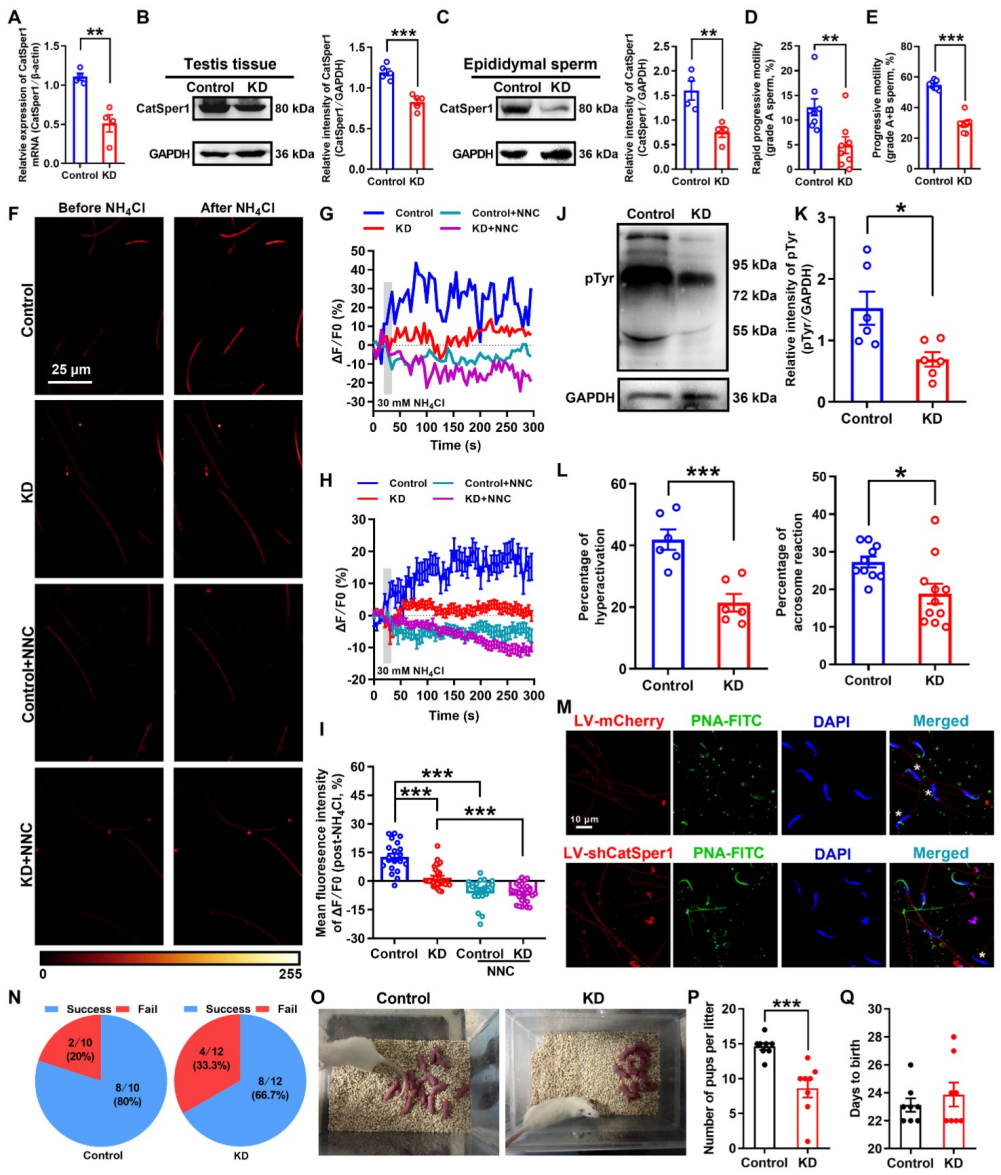
**Reduced sperm motility and functional characteristics, and impaired fertility in CatSper1 knockdown rats.** (A-C) Validation of CatSper1 knockdown (KD) in both testis tissues and epididymal sperm in rats treated with lentivirus-containing CatSper1 shRNA (LV-shCatSper1) by RT-qPCR (A) and Western blotting (B-C) (n = 4-5 rats per group). (D-E) Sperm motility including grade A sperm (D) and grade A+B sperm (E) (n = 8 rats per group). (F) Representative fluorescence images from Fluo-4 loaded sperm before and after 30 mM NH_4_Cl treatment in different groups as indicated. Scale bar = 25 μm. (G) Representative single sperm fluorescence traces. (H) Changes in normalized [Ca^2+^]_i_ fluorescent signals of all tested sperm. (I) Summary plot of normalized [Ca^2+^]_i_ fluorescent signals of all tested sperm in response to NH_4_Cl treatment (n = 21-30 spermatozoa from 4 rats per group). (J-M) Protein tyrosine phosphorylation (pTyr), hyperactivation, and acrosome reaction (AR) in the epididymal sperm of KD and control rats. A summary plot for the percentage of sperm acrosome reaction (L) and representative images of sperm acrosome reaction (M) of KD and control rats are shown (n = 6 rats per group). Asterisk indicates the sperm that has acrosome reaction (acrosome disappeared). Scale bar = 10 μm. (N-Q) *In vivo* fertility assay of KD and control rats (n = 6 male rats per group). (N) Summary plot of the pregnancy rate of female rats that were mated with KD and control rats. (O-P) Representative and a summary plot for number of pups per litter. (Q) Days to birth of the pup. All data are presented as mean ± SEM.^ *^P < 0.05, ^**^P < 0.01, ^***^P < 0.001. Unpaired *t* test for (A)-(E), (K)-(L), and (P)-(Q); one-way ANOVA with Sidak's *post-hoc* test for (I). See also Figures S5-S8.

To further determine our understanding that the reduction of CatSper channels in the sperm underlies the pathogenesis of AZS, we examined the effects of CatSper1 knockdown on the sperm motility and functional characteristics, and the fertility in normal male rats. Knockdown of CatSper1 in the testis tissues was performed by *in situ* injection of lentivirus-containing CatSper1 shRNA coupled to a mCherry or ZsGreen tag (LV-shCatSper1) into the testis tissues of normal male rats. The exact mechanism by which how the virus can pass through the blood-testis barrier and enter into the tubules is unclear. We speculate that the operation of *in situ* injection may promote some of the lentivirus passing through the blood-testis barrier, and gradually spreading into the near seminiferous tubules of injection point, and eventually entered into the whole tubules. Indeed, our results showed that on day 14 after lentivirus injection, approximately 30% seminiferous tubules (44 of 152 observed seminiferous tubules in LV-ZsGreen-infected rats; 47 of 156 observed seminiferous tubules in LV-shCatSper1-infected rats) emerged ZsGreen signals in the testis tissues of both LV-ZsGreen- and LV-shCatSper1-infected rats (Figure S5), and a prominent decrease in the mean fluorescence intensity of CatSper1 immunostaining was found in LV-shCatSper1-infected sperm compared with controls (Figure S6A-D). Also, a reduction of CatSper1 mRNA and protein abundance was found in both LV-shCatSper1-infected testis tissues and epididymal sperm (Figure 4A-C), indicating a significant efficiency of CatSper1 knockdown by *in situ* injection of LV-shCatSper1 into the testis tissues. Furthermore, by using CASA, we found a significant decrease in sperm motility including grade A sperm, grade A+B sperm (Figure 4D-E, and Video S4-1), and other parameters of sperm motility such as VSL, VAP, ALH, LIN, and STR, as well as the reduction of sperm viability in CatSper1 knockdown epididymal sperm (Figure S6E-L, and Video S4-1). Also, the NH_4_Cl-evoked, CatSper-mediated Ca^2+^ influx into sperm was decreased in the epididymal sperm of CatSper1 knockdown rats (Figure 4F-I, Figure S7, and Video S4-2 to S4-4). Consistently, we found a substantial decrease in the functional characteristics of epididymal sperm in CatSper1 knockdown rats, as manifested by a reduction of protein tyrosine phosphorylation, hyperactivation, and acrosome reaction (staining with PNA-FITC) in LV-shCatSper1-infected epididymal sperm (Figure 4J-M). Using the CatSper1 knockdown male rats, which were validated by reduced CatSper1 protein abundance in both testis tissues and epididymal sperm (Figure S8A-B) and decreased sperm motility (Figure S8C-L), to mate with normal female rats, the *in vivo* fertility assay showed that both the pregnancy rate of female rats and the pup numbers per litter were decreased (Figure 4N-Q). These results suggest that knockdown of CatSper1 in the testis tissues is sufficient to impair the sperm motility and functional characteristics, and thereby attenuating the fertility in normal male rats.

### Overexpression of CatSper1 rescues the CatSper1 knockdown-induced impairment of sperm motility and functional characteristics, and the fertility in rats

To further prove the above findings that knockdown of CatSper1 in the testis tissues is efficient to impair the sperm motility and functional characteristics, and the fertility in normal rats, we performed additional experiments to investigate whether overexpression (OE) of CatSper1 in the testis tissues could rescue the impaired sperm motility and functional characteristics, and the fertility in CatSper1-knockdown (KD) rats.

**Figure 5:**
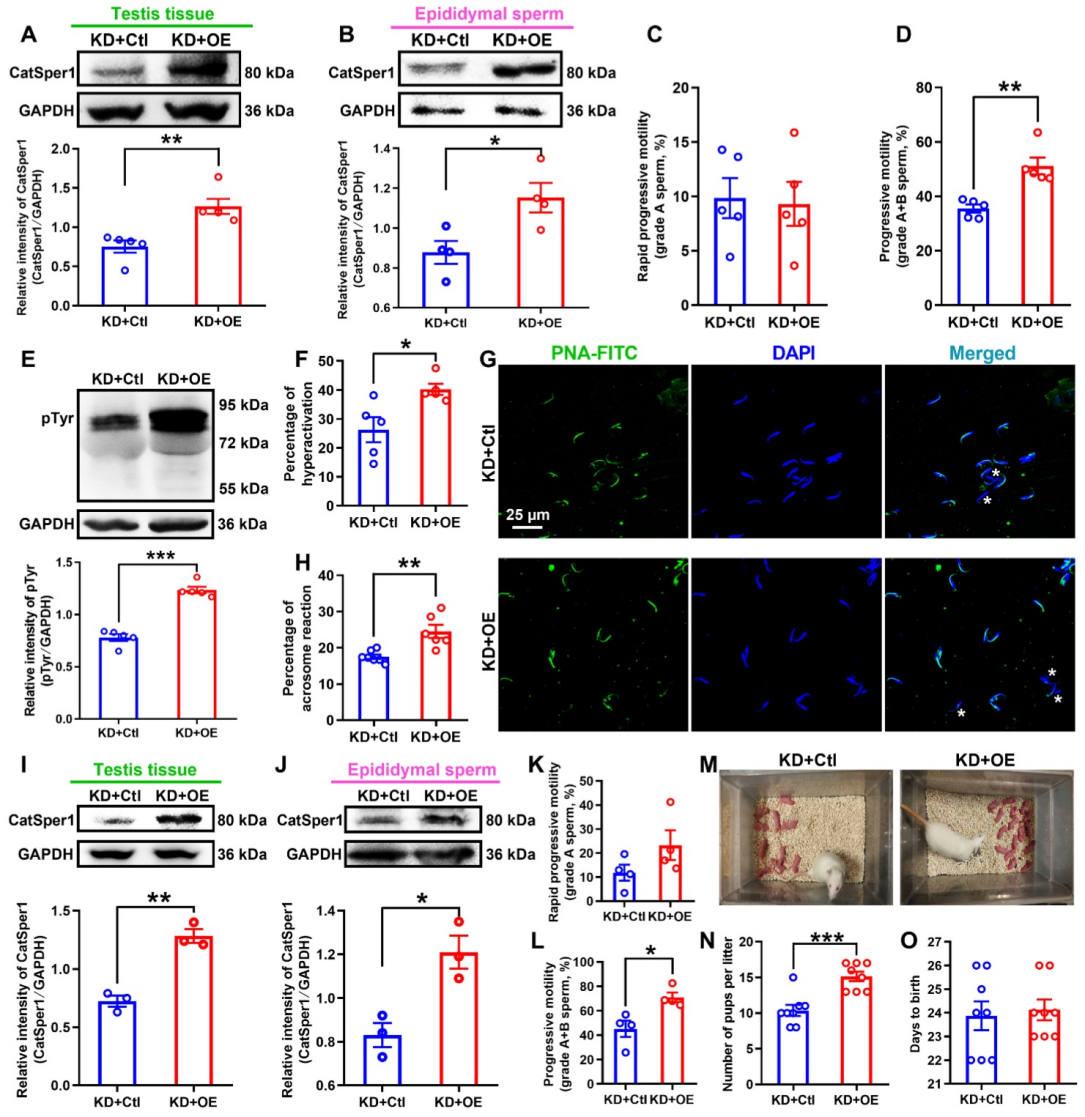
** CatSper1 overexpression rescues the impaired sperm motility and functional characteristics, and the fertility in CatSper1 knockdown rats.** (A-B) CatSper1 protein abundance in both testis tissues (A) and epididymal sperm (B) in LV-shCatSper1-infected (knockdown, KD) male rats with LV-CatSper1 (overexpression, OE) or empty viral vector (control, Ctl) treatment (n = 4-5 rats per group). (C-D) Sperm motility including grade A sperm (C) and grade A+B sperm (D) in rats of KD+OE and KD+Ctl groups (n = 5 rats per group). (E-H) Protein tyrosine phosphorylation (pTyr), hyperactivation, and acrosome reaction (AR) in the epididymal sperm of rats in KD+OE and KD+Ctl groups. Representative images (G) of sperm acrosome reaction and a summary plot for the percentage of sperm acrosome reaction (H) are shown (n = 5-7 rats per group). Asterisk indicates the sperm that has acrosome reaction (acrosome disappeared). Scale bar = 25 μm. (I-O) *In vivo* fertility assay of rats in KD+OE and KD+Ctl groups (n = 3-4 male rats per group). (I-L) Validation of the increased CatSper1 protein (I-J) and sperm motility (K-L) in CatSper1 KD male rats with LV-CatSper1 treatment, which were mated with the normal female rats. (M-N) Representative and a summary plot for number of pups per litter. (O) Days to birth of the pup. All data are presented as mean ± SEM.^ *^P < 0.05, ^**^P < 0.01, ^***^P < 0.001. Unpaired *t* test for (A)-(F), (H)-(L), and (N)-(O). See also Figures S9-S11.

Local overexpression of CatSper1 was performed by *in situ* injection of lentivirus-expressing CatSper1 coupled to ZsGreen tag (LV-CatSper1) into the testis tissues of rats on day 11 after the lentivirus-expressing CatSper1 shRNA coupled to mCherry tag (LV-shCatSper1) injection, and validation of lentiviral transfection efficiency was verified by immunofluorescence staining of CatSper1 with ZsGreen and mCherry, respectively (Figure S9A-C). The results revealed that accompanying with an elevated abundance of CatSper1 protein in both testis tissues and epididymal sperm in CatSper1-knockdown rats with locally overexpressed CatSper1 (Figure 5A-B, and Figure S9A-D), overexpression of CatSper1 in testis tissues rescued all the actions of CatSper1 knockdown-induced impairment of sperm motility and functional characteristics, and the fertility of LV-shCatSper1-treated rats. For example, the decreased sperm motility including grade A+B sperm, VCL, ALH, and LIN were restored (Figure 5C-D, and Figure S9E-L); the reduced protein tyrosine phosphorylation, hyperactivation and acrosome reaction of epididymal sperm were elevated (Figure 5E-H); and also, using the CatSper1 OE-rescued CatSper1-KD male rats, which were validated by increased CatSper1 protein abundance in both testis tissues and epididymal sperm (Figure 5I-J), and improved sperm motility (Figure 5K-L, and Figure S10), to mate with normal female rats, the reduced pup numbers per litter were restored (Figure 5M-O). To our surprise, overexpression of CatSper1 in the testis tissues of AZS rats, which were validated by increased expression of CatSper1 mRNA and protein abundance using immunofluorescence staining, RT-qPCR and Western blotting assay (Figure S11A-F), almost had no influence on sperm motility (*i.e*. grade A sperm, VSL, VCL, VAP, ALH, LIN, and STR) and functional characteristics (*i.e*. protein tyrosine phosphorylation, hyperactivation, and acrosome reaction) of the epididymal sperm to AZS rats (Figure S11G-S), indicating that merely CatSper1 overexpression in the testis tissues was not sufficient to rescue the impaired sperm motility and functional characteristics of AZS rats.

### Improvement of sperm motility and functional up-regulation of CatSper channels in the sperm of AZS rats by 2 Hz-EA treatment

**Figure 6:**
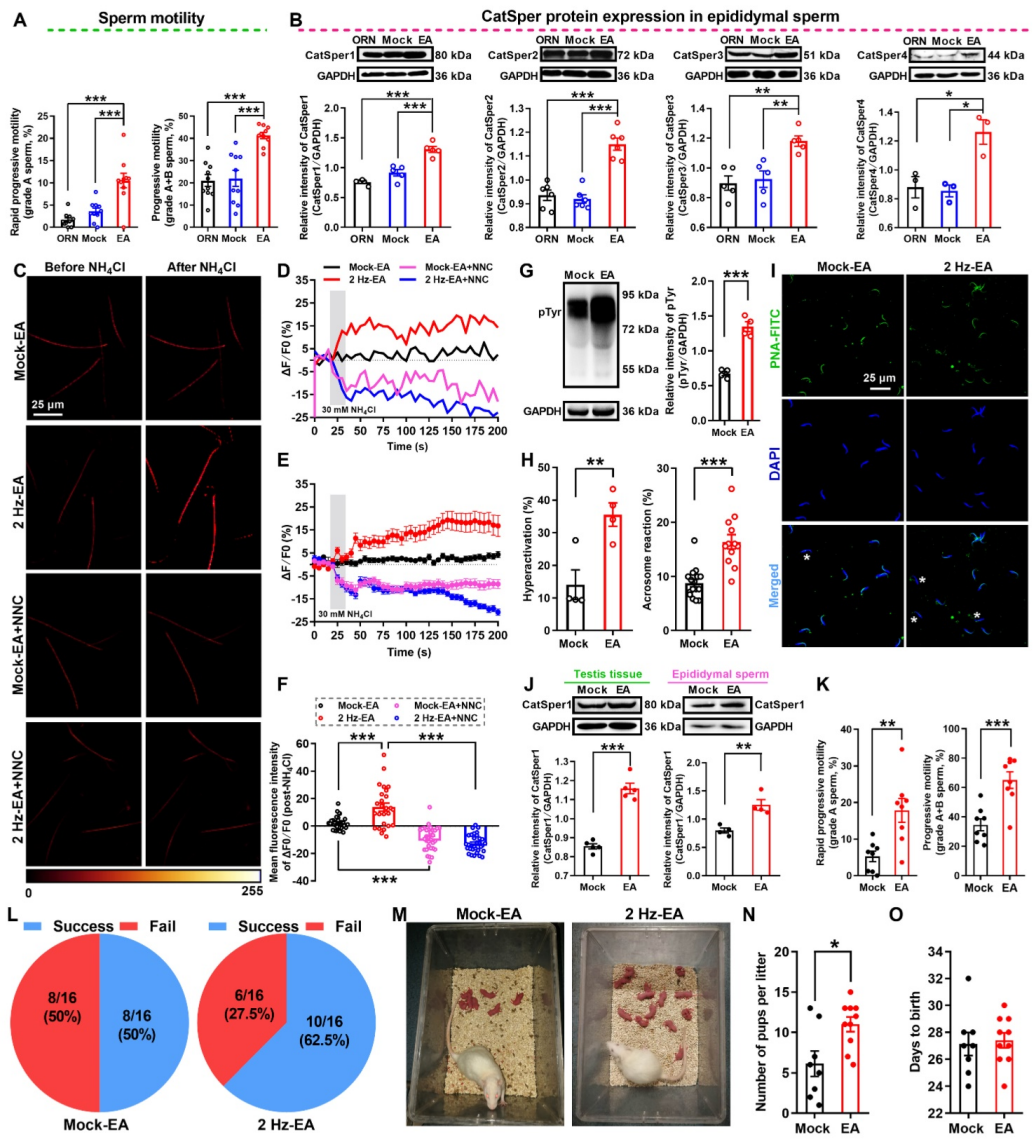
** 2 Hz-EA treatment improves the sperm motility and functional CatSper channels expression in the spermatozoa, and enhances the fertility of asthenozoospermic (AZS) model rats.** (A) Sperm motility including grade A and grade A+B sperm (n = 10 rats per group). (B) CatSper (from CatSper1 to CatSper4) protein abundance (n = 3-6 rats per group). (C) Representative fluorescence images from Fluo-4 loaded sperm before and after administering 30 mM NH_4_Cl in different groups as indicated. Scale bar = 25 μm. (D) Representative single sperm fluorescence traces. (E) Changes in normalized [Ca^2+^]_i_ fluorescent signals of all tested sperm. (F) Summary plot of normalized [Ca^2+^]_i_ fluorescent signals of all tested sperm in response to NH_4_Cl treatment (n = 26-34 spermatozoa from 6-8 rats per group). (G-I) Protein tyrosine phosphorylation (pTyr) (G), hyperactivation (H), and acrosome reaction (AR) (I) in the epididymal sperm. A summary plot for the percentage of sperm acrosome reaction (H) and representative images of sperm acrosome reaction (I) are shown (n = 4 rats per group). Asterisk indicates the sperm that has acrosome reaction (acrosome disappeared). Scale bar = 25 μm. (J-O) *In vivo* fertility assay for 2 Hz EA- and mock EA-treated AZS rats (n = 4-8 male rats per group). (J-K) Validation of the increased CatSper1 protein (J) and sperm motility (K) for the 2 Hz EA-treated male rats that to mate with the normal female rats. (L) The pregnancy rate of female rats that were mated with the 2 Hz EA- and mock EA-treated AZS male rats. (M-N) Representative and a summary plot for number of pups per litter. (O) Days to birth of the pup. All data are presented as mean ± SEM.^ *^P < 0.05, ^**^P < 0.01, ^***^P < 0.001. Unpaired *t* test for (A)-(B), (G)-(H), (J)-(K), and (N)-(O); one-way ANOVA with Sidak's *post-hoc* test for (F). See also Figures S12-S15.

As shown in the abovementioned data (Figure 2), 2 Hz-TEAS treatment could improve the sperm motility of iAZS patients via functional up-regulation of CatSper channels in the sperm. Here, we investigated whether 2 Hz-EA treatment could exert the same action in AZS model rats. Firstly, we determined the effective protocol for 2 Hz-EA treatment on the sperm quality of AZS rats. The results revealed that the protocol of 2 Hz-EA treatment to AZS rats, either by once per day for three or five times, or by once every other day for three times, almost had no significant effect on the sperm quality of AZS rats (Figure S12), while 2 Hz-EA treatment, by the protocol of once every other day for five times (hereafter referred to as 2 Hz-EA treatment), could significantly improve the sperm quality of AZS rats, as manifested by increased sperm motility including grade A sperm, grade A+B sperm, and other parameters of sperm motility such as VSL, VAP, ALH, and LIN, as well as the elevated sperm viability (Figure 6A, Figure S13, and Video S5-1). Secondly, we examined the effects of 2 Hz-EA treatment on functional expression of CatSper channels in the epididymal sperm of AZS rats. The results showed that 2 Hz-EA treatment could abrogate the reduced abundance of CatSper protein (CatSper1-CatSper4) in the epididymal sperm of AZS rats (Figure 6 B). Also, the decreased abundance of both CatSper mRNA and protein (CatSper1-CatSper4) in the testis tissues of AZS rats was effectively restored by 2 Hz-EA compared to mock-EA treatment (Figure S14). In line with these findings, the reduction of NH_4_Cl-evoked, CatSper-mediated [Ca^2+^]_i_ fluorescent signals in the epididymal sperm of AZS rats was rescued by 2 Hz-EA treatment (Figure 6C-F, and Video S5-2 to S5-4), and the impaired sperm function such as the reduction of protein tyrosine phosphorylation, hyperactivation, and acrosome reaction (staining with PNA-FITC) in the epididymal sperm of AZS rats was reversed by 2 Hz-EA compared with mock-EA treatment (Figure 6G-I). Using the 2 Hz EA-treated AZS male rats, which were validated by an elevated abundance of CatSper1 protein in both testis tissues and epididymal sperm (Figure 6J) and improved sperm motility (Figure 6K and Figure S15), to mate with normal female rats, the *in vivo* fertility assay showed that both the pregnancy rate of female rats and the pup numbers per litter were increased (Figure 6L-O). Taken together, these results suggest that 2 Hz-EA treatment to AZS rats could increase the expression and function of CatSper channels in the sperm, subsequently improving the sperm motility and functional characteristics of epididymal sperm and restoring the impaired fertility of AZS rats.

### Knockdown of CatSper1 abolishes the therapeutic effects of 2 Hz-EA treatment on AZS rats

To further determine whether 2 Hz-EA treatment exerts its actions on AZS rats through inducing functional up-regulation of CatSper channels in the sperm, we examined effects of CatSper1 knockdown on the therapeutic effects of 2 Hz-EA treatment to AZS rats.

**Figure 7:**
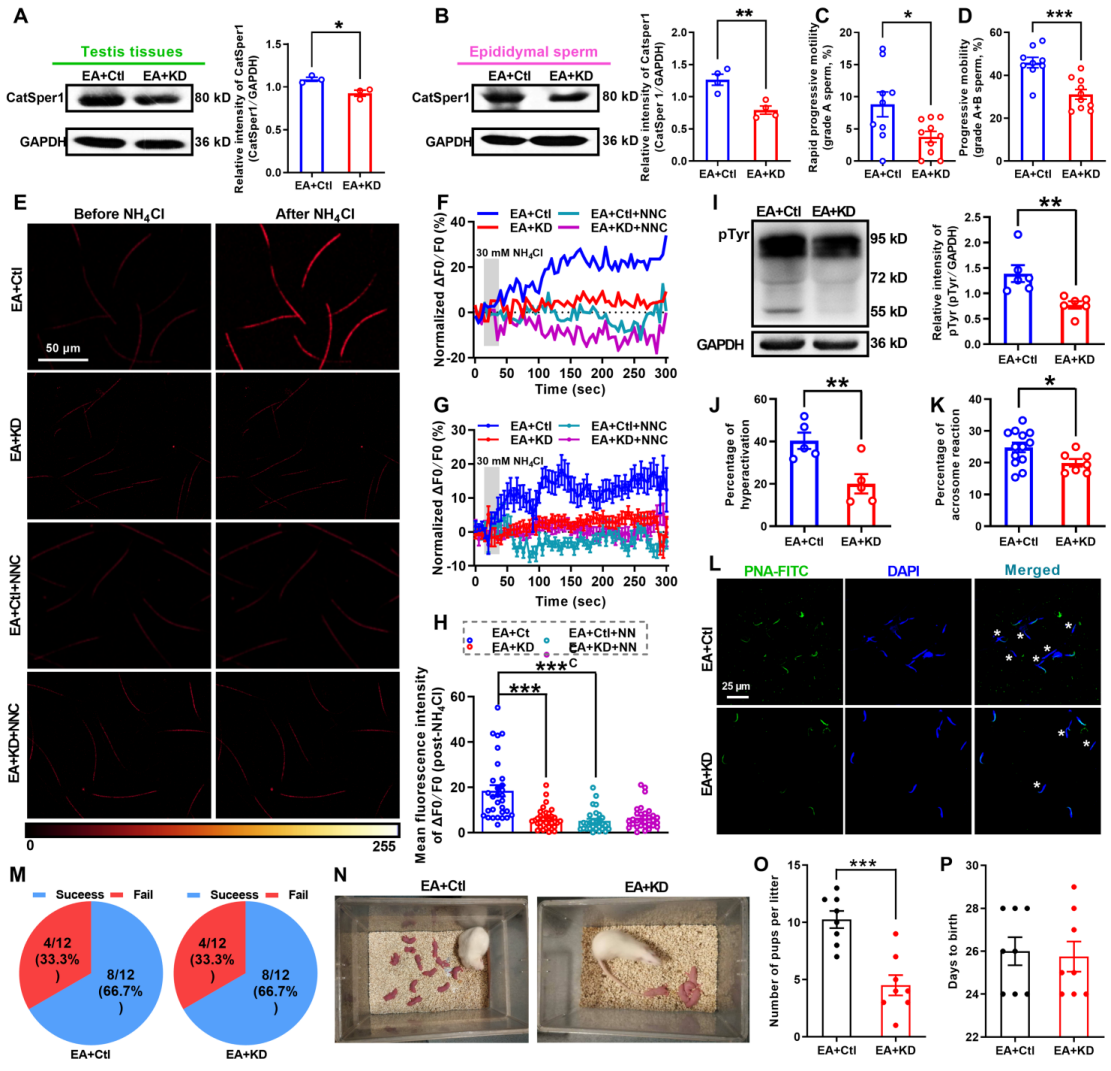
** CatSper1 knockdown abolishes the therapeutic effect of 2 Hz-EA treatment on asthenozoospermic (AZS) model rats.** (A-B) CatSper1 protein abundance in both testis tissues (A) and epididymal sperm (B) in 2 Hz EA-treated AZS rats with locally delivering LV-shCatSper1 (knockdown, KD) or empty viral vector (control, Ctl) (n = 3-4 rats per group). (C-D) Sperm motility including grade A and grade A+B sperm in rats of EA+KD and EA+Ctl groups (n = 9-10 rats per group). (E) Representative fluorescence images from Fluo-4 loaded sperm before and after administering 30 mM NH_4_Cl in different groups as indicated. Scale bar = 50 μm. (F) Representative single sperm fluorescence traces. (G) Changes in normalized [Ca^2+^]_i_ fluorescent signals of all tested sperm. (H) Summary plot of normalized [Ca^2+^]_i_ fluorescent signals of all tested sperm in response to NH_4_Cl treatment (n = 28-33 spermatozoa from 4 rats per group). (I-L) Protein tyrosine phosphorylation (pTyr), hyperactivation, and acrosome reaction (AR) in the epididymal sperm of rats in EA+KD and EA+Ctl groups. A summary plot for the percentage of sperm acrosome reaction (K) and representative images of sperm acrosome reaction (L) are shown (n = 5-6 rats per group). Asterisk indicates the sperm that has acrosome reaction (acrosome disappeared). Scale bar = 25 μm. (M-P) *In vivo* fertility assay of rats in EA+KD and EA+Ctl groups (n = 4-6 male rats per group). (M) Summary plot of the pregnancy rate of female rats that were mated with 2 Hz EA-treated, CatSper1 KD (or vector control) AZS rats. (N-O) Representative and a summary plot for number of pups per litter. (P) Days to birth of the pup. All data are presented as mean ± SEM.^ *^P < 0.05, ^**^P < 0.01, ^***^P < 0.001. Unpaired *t* test for (A)-(D), (I)-(K), and (O)-(P); one-way ANOVA with Sidak's *post-hoc* test for (H). See also Figures S16-S18.

Knockdown of CatSper1 in the testis tissues was performed by *in situ* injection of LV-shCatSper1 into the testis tissues of rats on day 11 after ORN administration, and then the EA treatment was applied to ORN-treated rats as described in the method section. Validation of lentiviral transfection efficiency and CatSper1 knockdown were verified by a decreased mean fluorescence intensity of CatSper1 immunostaining (Figure S16A-D) and a reduction of CatSper1 protein abundance in both testis tissues and epididymal sperm (Figure 7A-B). These results revealed that CatSper1 knockdown almost abolished all the improvement effects of 2 Hz-EA treatment to AZS rats. For instance, the improvement of sperm motility (Figure 7C-D, Figure S16E-L, and Video S6-1) and the elevation of the NH_4_Cl-evoked, CatSper-mediated Ca^2+^ influx into sperm in AZS rats treated with 2 Hz-EA were abrogated by CatSper1 knockdown (Figure 7E-H, Figure S17, and Video S6-2 to S6-4). Also, the 2 Hz EA-induced increases in protein tyrosine phosphorylation, hyperactivation, and acrosome reaction of epididymal sperm in AZS rats were abolished by CatSper1 knockdown (Figure 7I-L). In the *in vivo* fertility assay, when using the 2 Hz EA-treated, CatSper1-knockdown AZS male rats, which were validated by reduced CatSper1 protein abundance in both testis tissues and epididymal sperm and impaired sperm motility (Figure S18), to mate with normal female rats, the 2 Hz EA-induced increase in the pup numbers per litter was abrogated by CatSper1 knockdown in the testis tissues (Figure 7M-O). Taken together, we suggest that the functional down-regulation of CatSper channels in the sperm may be a contributor for a portion of AZS, especially iAZS, while 2 Hz-EA treatment exerts its therapeutic actions to AZS rats through inducing functional up-regulation of CatSper channels in the sperm.

## Discussion

In this study, we present several lines of evidence demonstrating the involvement of decreased CatSper channels in the pathogenesis of some AZS, especially iAZS, while 2 Hz-TEAS or EA treatment has a therapeutic effect on iAZS through inducing the functional up-regulation of CatSper channels in the sperm. This study provides a novel mechanism for the pathogenesis of iAZS and presents a potential therapeutic target of CatSper for iAZS treatment. Acupuncture treatment like TEAS may be used as a promising complementary and alternative medicine (CAM) therapy for male infertility caused by iAZS in clinical practice.

As a sperm-specific calcium channel that allows calcium influx into sperm, CatSper channel plays a vital role in regulating sperm motility, hyperactivity and male fertility 2-4. A reduction of CatSper channels in the sperm may result in the impairment of sperm motility and hyperactivity, and ultimately lead to male infertility 39. In agreement with this understanding, we indeed found a substantial decrease in CatSper protein abundance from CatSper1 to CatSper4 in the sperm of iAZS patients. Also, we observed a significant decrease in abundance of CatSper protein (CatSper1-CatSper4) in the epididymal sperm of ORN-induced AZS model rats, and a reduction of both CatSper mRNA and protein (CatSper1-CatSper4) levels in testis tissues of AZS model rats. Similar decreases in CatSper1, 2, 3, and 4 gene levels as well as in CatSper1 and CatSper2 protein expression were also found in the sperm of infertile men with iAZS 19, 20, 22, 23. Accompanying with the decreased expression of CatSper channels in the sperm, we revealed that the NH_4_Cl-evoked, CatSper-mediated Ca^2+^ influx into sperm was correspondingly reduced in both iAZS patients and ORN-induced AZS rats; and an impaired sperm function characterized by reduced protein tyrosine phosphorylation, hyperactivation, and acrosome reaction emerged in the epididymal sperm of AZS rats, which may explain the impaired fertility of AZS male rats. Likewise, CatSper1 or CatSper2 null mouse spermatozoa exhibits a decreased sperm motility, abnormal flagellar beating, as well as lacked hyperactivity and acrosome reaction, thus leading to the complete infertility of knockout male mice 4, 40-42. In addition, male mice lacking CatSper genes 5-8, as well as men with genetic mutation of CatSper ion channels 9-13, are completely infertile due to the lack of sperm hyperactivation.

The contribution of decreased sperm CatSper channels to the pathogenesis of AZS in rat model was validated by local CatSper1 knockdown (KD) or overexpression (OE) experiments. In our present study, only CatSper1 KD or OE was applied to rats with consideration of the animal's tolerance to lentivirus. As expected, knockdown of CatSper1 in the testis tissues is sufficient to impair sperm motility and functional characteristics, and thereby attenuating the fertility in normal male rats, while overexpression of CatSper1 rescues all the actions of CatSper1 KD to those rats. However, merely CatSper1 overexpression in the testis tissues cannot restore the impaired sperm motility and functional characteristics of AZS rats. We speculated that since all the four pore-forming α CatSper subunits are necessary for sperm motility and functional characteristics 7, therefore gene silence for one of the four pore-forming α subunits (*e.g.* CatSper1 knockdown) is sufficient to impair the sperm motility and functional characteristics, and the fertility in normal male rats. However, because all the four α CatSper subunits (CatSper1-CatSper4) are decreased in ornidazole-induced AZS rats, thus merely overexpressing CatSper1 is not sufficient to rescue the impaired sperm motility and functional characteristics of AZS rats.

In support of this idea, either injection of a CatSper1 DNA vaccine into the muscle of male mice or knockdown of CatSper2 in male rats can impair the sperm motility and hyperactivation, and causes the subfertility of the animals 43, 44. Taken together, we suggest that decrease in one of the four α subunits of CatSper channels (CatSper1-CatSper4) in the sperm is sufficient to impair sperm motility and functions, and ultimately damages the fertility of male patients or animals. Therefore, a reduction of CatSper channels in the sperm may be a suggested contributor for the pathogenesis of AZS, especially iAZS.

In addition to SJS, a traditional Chinese medicine (TCM) recipe that has been proved to improve the sperm motility of AZS rats by enhancing sperm CatSper1 channels expression in our previous study 24, we here demonstrated that both EA and TEAS, which are originated from TCM, also have an evident therapeutic effect on iAZS patients and AZS model rats via the functional up-regulation of CatSper channels in the sperm. Associated with the improvement of sperm motility and functional up-regulation of CatSper channels in the sperm by 2 Hz-TEAS to iAZS patients or 2 Hz-EA to AZS rats, we found that the impaired sperm function including the decreased protein tyrosine phosphorylation, hyperactivation, and acrosome reaction of the epididymal sperm, as well as the impaired fertility of AZS rats were reversed by 2 Hz-EA treatment. The therapeutic effects of 2 Hz-EA to AZS rats through functionally upregulating CatSper channels in the sperm was validated by CatSper1-knockdown experiments which were proved to abolish all the improvement effects of 2 Hz-EA treatment to AZS rats. As mentioned above, because all the four CatSper channel proteins (CatSper1-CatSper4) are required for the functional characteristics of the channel, the sperm motility, and male fertility 7, so knocking down any one of CatSper channel proteins like CatSper1 is sufficient to impair the functional activity of the channel, and subsequently abolish the therapeutic effects of 2 Hz-EA treatment to AZS rats. Of course, although acupuncture like 2 Hz TEAS and EA could upregulate the CatSper (from CatSper1 to CatSper4) mRNA and protein expression in both iAZS patients and AZS model rats, the therapeutic effects of acupuncture on AZS caused by total CatSper1 or other CatSper channels defects (such as gene defect) still may be limited for somewhat. Nevertheless, acupuncture treatment has been shown to improve the sperm quality of male infertility resulted from semen non-liquefaction, azoospermia, male immune infertility or oligoteratoastheno-zoospermia 45-49. Also, acupuncture treatment exerts a general improvement to the ultrastructural integrity of spermatozoa and the sperm motility for patients with oligoasthenozoospermia or asthenoteratozoospermia 26, 50, 51. In addition, application of alginate oligosaccharides, the degradation products of alginate to busulfan-treated male mice can also rescue their impaired sperm motility and concentration 52, and with the treatment of vitamin-E, selenium or panax ginseng to male aging mice significantly improves the sperm quality including sperm motility and hyperactivation via up-regulating CatSper gene expression 53-55.

In this study, we found that 100 Hz-TEAS treatment could also improve the sperm quality of iAZS patients (Figure 2A-D). However, in spite of that the effective rate of 100 Hz-TEAS was even higher than 2 Hz-TEAS to iAZS patients, no significant changes in CatSper1 and CatSper3 protein expression were found in the sperm of 100 Hz TEAS treated-iAZS patients (Figure S1). Similar results were also observed in AZS model rats, in which 100 Hz-EA treatment exerted an improvement effect on the sperm quality of AZS rats (Figure S19), but had no effect on CatSper protein (from CatSper1 to CatSper4) expression in epididymal sperm (Figure S20). We speculated that the potential mechanism of 100 Hz-TEAS (or EA) treatment for the improvement of sperm quality of iAZS patients and AZS model rats is probably different from 2 Hz-TEAS (or EA) treatment, where the former is not dependent upon CatSper channels whereas the latter is dependent. The frequency-dependent characteristics of acupuncture treatment have been documented solidly in previous studies 56, 57, *e.g.* low frequency (2 Hz) and high frequency (100 Hz) of EA selectively induces the release of enkephalins and dynorphins, and correspondingly binding to μ- and κ-opioid receptors, respectively, to play different roles 56. It has been revealed that δ-, κ-, and μ-opioid receptors are expressed in the sperm and testis tissues of both human beings and animals 58-61, and mutation of μ-opioid receptor results in the impairment of sperm motility and fertility to male mice 62. Application of κ-opioid receptor agonist or antagonist can correspondingly increase and decrease the boar sperm motility in the *in vitro* study 63, and 100 Hz-EA stimulation promotes the release of dynorphin and the activation of κ-opioid receptor 56. From the abovementioned understanding, we suggest that low frequency (2 Hz) of TEAS (or EA) probably exerts its improvement effects on sperm motility of iAZS patients and AZS model rats through up-regulating sperm CatSper channels, which may depend on inducing the release of enkephalins and the activation of μ-opioid receptors in the sperm. In contrast, that high frequency (100 Hz) of TEAS (or EA) improves the sperm motility of iAZS patients and AZS rats is independent of CatSper channels but likely relies on the activation of κ-opioid receptor in the sperm. In support of this idea, we indeed found that 100 Hz-EA treatment to AZS rats could induce a significant increase in expression of κ-opioid receptor (KOR) in the sperm (Figure S21), and local administration of KOR antagonist norbinaltorphimine (Nor-BNI) into the testes disrupted the improvement effects of 100 Hz-EA treatment on the sperm motility of AZS rats (Figure S22). Certainly, if the AZS patients have loss of function mutations in CatSper channel genes, 2 Hz-TEAS treatment may improve the sperm motility of AZS patients by activating μ-opioid receptor and the downstream signaling pathways, while 100 Hz-TEAS treatment can also rely on the activation of κ-opioid receptor in the sperm.

Compared to other therapies such as Chinese herbal medicine or intracytoplasmic sperm injection (ICSI), acupuncture treatment like EA or TEAS has more advantages including non-invasive, more cost-efficient, low risks, and with less adverse effects. Moreover, the safety of acupuncture therapy to patients with infertility has been widely ascertained in clinical practices 64-66. Therefore, acupuncture treatment like TEAS may be used as a complementary and alternative medicine (CAM) utilization for male infertility especially iAZS patients. Of course, there are several lines of limitation of acupuncture therapy to AZS. For example, some types of AZS caused by CatSper or other channel defects, or caused by certain genetic factors are probably not suitable for acupuncture treatment. So, acupuncture like TEAS may be mainly suitable for the treatment of idiopathic asthenospermia (iAZS). Also, acupuncture is only a CAM therapy, it may be used in combination with other therapies such as moxibustion therapy, physical therapy, medicines, or Chinese herbal medicine, *etc*. Nevertheless, our current results raised a possibility of acupuncture like TEAS as a promising CAM therapy for male infertility caused by iAZS in clinical practice.

In conclusion, our present data suggest that a reduction of functional CatSper channels in the sperm may result in the impairment of sperm motility, hyperactivation, and acrosome reaction of the epididymal spermatozoa. Therefore, a decreased CatSper channels in the sperm may be a contributor or a downstream indicator for a portion of AZS, especially iAZS. Acupuncture treatment like TEAS or EA at 2 Hz frequency may improve the sperm quality for certain iAZS patients and AZS model rats through inducing the functional up-regulation of CatSper channels in the sperm. This study provides a novel mechanism for some AZS especially iAZS, and presents a potential therapeutic target of CatSper for iAZS treatment. Acupuncture treatment like TEAS may be used as a promising CAM therapy for male infertility caused by iAZS in clinical practice.

## Materials and Methods

### Chemicals, reagents, and antibodies

Ornidazole (Meilun Biotechnology, Dalian, China) was dissolved in 0.2% carboxymethylcellulose sodium (CMC-Na) solution immediately before administration. NNC 55-0396 (Sigma-Aldrich) was dissolved in ddH_2_O to make a stock solution and stored at -20 °C. Percoll solution was purchased from Sigma-Aldrich and was stored at 4 °C. Fura-2 AM (Molecular Probes) were dissolved in dimethyl sulfoxide (DMSO; Sigma-Aldrich) to make a stock solution and stored at -20 °C, and 0.05% pluronic F-127 (Molecular Probes) was stored at room temperature. Fluo-4 AM (Molecular Probes) and arachis hypogaea (peanut) agglutinin (fluorescein isothiocyanate-conjugated peanut agglutinin (PNA-FITC), Sigma-Aldrich) were dissolved in dimethyl sulfoxide (DMSO; Sigma-Aldrich) to make a stock solution and stored at -20 °C. The stock solution was subsequently diluted with sterile normal saline to make desired final concentrations immediately before administration. The final concentration of DMSO was less than 0.5%. Lentivirus-containing CatSper1 shRNA coupled to a mCherry or ZsGreen tag (LV-shCatSper1, 5×10^8^ TU/mL) and lentivirus delivering a construct that contained CatSper1 plasmid coupled to a ZsGreen tag (LV-CatSper1, 5×10^8^ TU/mL) were purchased from Likeli Technologies (Beijing, China). Nor-Binaltorphimine (Abcam) was dissolved in normal saline to make a stock solution and stored at -20 °C. The stock solution was subsequently diluted with sterile normal saline to make desired final concentrations immediately before administration. All other chemicals or reagents were obtained from Sigma-Aldrich except as mentioned in the text. All chemicals, reagents, antibodies, and lentivirus for experiments in this study are listed in Supplementary table S1 (Key resources table).

### Participants

Potential cases were screened from men who sought for couple infertility treatment in the Reproductive Medicine Center and Department of Urology of the Third Hospital, Peking University between June 2014 and October 2020. A total of 112 male patients with idiopathic asthenozoospermia (iAZS) infertility and 35 healthy fertile men who had normal sperm quality and had a successful reproductive history during the most recent 2 years were recruited for research. Before inclusion in the study, participants underwent a standardized clinical and laboratory evaluation including measurements of the size and volume of the testes, assessment for hydrocele, varicocele, secondary sexual characteristics and routine semen analysis (*e.g.* semen volume, sperm concentration, sperm motility, sperm viability, and sperm morphology). The details of the participants' lifestyle, habits and family history were also recorded.

Diagnostic criteria of idiopathic AZS (iAZS) infertility were based on the WHO laboratory manual for the examination and processing of human semen, 5th ed, in 2010 67. To be specific, the following five criteria should all be met: (1) the inability of a sexually active, non-contracepting couple to achieve pregnancy after 12 months, due to the reason of the man; (2) two or more semen examinations (abstinence time for 3-7 days each time) of male suggested asthenozoospermia: progressive motility (PR) <32%, or PR + non-progressive motility (NP) <40%; (3) sperm concentration >15 × 10^6^ sperm/mL; (4) proportion of normal sperm ≥ 4%; and (5) no obvious causative factors were found.

The inclusion criteria are as follows: (1) males, aged between 22 and 45 years; (2) confirmed the diagnosis of male infertility and idiopathic AZS; (3) with normal sexual function, regular sexual life; and (4) willing to join this research and sign an informed consent form.

The exclusion criteria are as follows: (1) with serious congenital testicular and genital dysplasia or deformity, or clear factors induced spermatogenesis dysfunction; (2) with clear oligozoospermia, azoospermia, or teratozoospermia such as multiple morphological abnormalities of the flagella (MMAF); (3) with abnormal sex hormone, seminal plasma biochemistry, or seminal plasma elastase; (4) with reproductive system infection, such as chlamydia trachomatis or mycoplasma infection; (5) with mixed antiglobulin reaction test for anti-sperm antibodies (+); (6) with a genealogy that had reported fertility problems, or a history of previous infertility treatment; (7) use drugs affecting the experimental study within 3 months; (8) with mental diseases, malignant tumor, or serious organic diseases; (9) participated in other clinical trials in the past 3 months, or termination of the test early by participant himself.

For determining alterations of CatSper channels' expression and function in the sperm of iAZS patients, 35 iAZS patients and 35 healthy subjects (HS) were recruited and examined (Figure 1), while for investigating the effects of TEAS treatment on asthenozoospermia, 77 iAZS patients were randomly assigned to three groups using a computerized randomization method (Figure 2): mock TEAS group (n = 20), 2 Hz-TEAS group (n = 41) and 100 Hz-TEAS group (n = 16).

### Ethics approval

The present study was approved by the Institutional Review Board (IRB) of Peking University (Permit number: IRB00001052-13004) and the study protocol was prospectively registered on Chinese Clinical Trial Registry (Identifier: ChiCTR-INR-16008604). All participants were voluntary and have read and signed informed consent forms before data collection or treatment.

### TEAS treatment for iAZS patients

Patients in TEAS group received a-2 Hz or 100 Hz-TEAS treatment. Traditional acupuncture points of BL23 (Shenshu), ST36 (Zusanli), CV1 (Huiyin) and CV4 (Guanyuan) were applied for their improvement of sperm parameters 26, 68-70. The acupoints were connected to electrical stimulation generator (HANS, 200A, Hua Yun An Te Co., Ltd, Beijing, China) through an electrode patch placed on the skin surface. Continuous waveform mode was selected, and the stimulation with 2 Hz or 100 Hz frequency was generated at an intensity of 7 to 15 mA (adjusted every 10 min to keep the patients comfortable) for 30 min once a day for 60 days. Patients in mock-TEAS group were given lifestyle advice without TEAS treatment. The study was designed as single-blind, in which only the trial designer known the random codes and treatment measures, researchers were unaware of the randomization scheme.

### Purification, cryopreservation and thawing of human semen

Purification of human sperm was prepared by a discontinuous density gradient procedure 71. Briefly, 2 mL of semen was loaded on top of a 40-80% Percoll suspension, diluted with Earle's balanced salt solution (10×). After density gradient centrifugation (300 g, 20 min), the deposit was re-suspended in human tubal fluid (HTF) medium containing (in mM): 97.8 NaCl, 4.69 KCl, 4 NaHCO_3_, 0.37 KH_2_PO_4_, 2.04 CaCl_2_, 0.2 MgCl_2_, 21.4 lactic acid, 21 HEPES, 2.78 glucose, and 0.33 Na-pyruvate, pH 7.3 (adjusted with NaOH). Cryopreservation and thawing of human semen were prepared as described in elsewhere 72, 73, samples were mixed with equal proportions of test-yolk buffer (glycerol 14%; egg yolk 30%; glucose 1.98% and sodium citrate 1.72%) and gradient cryopreserved. For thawing of human semen, the samples were thawed at room temperature, immersed in water bath at 37 °C for 30 s, washed three times with PBS (without Ca^2+^ or Mg^2+^) at room temperature (centrifuged by 600 g for 5 min), then the deposit was re-suspended in HTF solution.

### Semen analysis for patients

Semen analysis was performed by a computer-assisted semen analysis (CASA) system (WLJY-9000, Beijing Weili new century science and technology development Co., Ltd, China) according to laboratory manual of WHO for semen volume, sperm concentration, and sperm motility 67. The following parameters were evaluated: rapid progressive motility (grade A) (%), progressive motility (grade A+B) (%), straight-line velocity (VSL, μm/s), curve-line velocity (VCL, μm/s), average path velocity (VAP, μm/s), amplitude of lateral head displacement (ALH, μm), linearity (LIN, %), straightness (STR) and wobble (WOB, %). Sperm concentration, expressed as ×10^6^/mL, was determined by hemocytometer method on two separate preparations of the semen sample.

Sperm viability was visualized by eosin and nigrosin staining 24. Briefly, one drop of sperm suspension was mixed with two drops of 1% eosin Y for 30 s, then three drops of 10% nigrosin was added and mixed them well. A drop of the mixture was added on a clean glass slide to make a smear, and the slides were air-dried. Pink-stained dead spermatozoa and unstained live spermatozoa were counted under a light microscope and the percentage of viable spermatozoa was the viability of spermatozoa. A minimum of 200 sperm were counted for each assay.

### Animals

Sexually mature male Sprague-Dawley rats weighing 230-250 g at the beginning of the experiment were provided by the Department of Experimental Animal Sciences, Peking University Health Science Center. The rats were housed in separated cages with free access to food and water. The room temperature was kept at 24 ± 1 °C under a 12-h light/12-h dark cycle. All animal experimental procedures were approved by the Animal Care and Use Committee of Peking University.

### Animal model of asthenozoospermia

A rat model of asthenozoospermia (AZS) was developed by intragastric administration of ornidazole (ORN) as described elsewhere 34. Briefly, ORN at a dose of 400 mg/kg body weight was intragastrically administered into adult male rats once per day for 14 consecutive days. The control rats received a 0.2% carboxymethylcellulose sodium (CMC-Na) solution (vehicle of ORN) throughout the experiment. At the end of day 14 after the last drug administration, the rats were euthanized with 1% pentobarbital sodium, and the epididymis were quickly removed for further examination. Development of the rat model of AZS was determined by assessment of the epididymal sperm motility and count in rats.

### Electroacupuncture treatment for animals

Electroacupuncture (EA) treatment was applied to rats according to the method previously described 74. Briefly, rats were restrained in rodent holders with their hind legs and tails protruding. Sterile acupuncture needles (0.2 mm in diameter, 5 mm in length; Beijing Zhongyan Taihe Medical Instrument Co., Ltd) were inserted perpendicularly into the acupuncture points of "Shenshu" (BL 23, bilateral) and "Zusanli" (ST 36, bilateral). Electrical stimulation of a constant current was generated from Han's Acupoint Nerve Stimulator (HANS, LH202H, Beijing Astronautics and Aeronautics Aviation University, Beijing, China). The stimulation with 2 Hz or 100 Hz frequency was generated at an intensity of 1-2-3 mA (increasing 1 mA per 10 min) for 30 min once a day or once every other day for 3 times or 5 times. Mock EA groups were treated with a similar procedure except that the output leads of the stimulator were disconnected. AZS model rats were intragastric administration of ornidazole (ORN, 400 mg/kg/d) once per day after the EA treatment till the end of treatment. Effects of EA treatment on AZS also were determined by assessment of the epididymal sperm motility and count in rats.

### Sperm morphology, motility and count

Cauda epididymal sperm of rats were collected and prepared as described elsewhere 24. In brief, two caudal epididymis were placed in modified HEPES medium containing 120 mM NaCl, 2 mM KCl, 1.2 mM MgSO_4_·7H_2_O, 0.36 mM NaH_2_PO_4_, 25 mM NaHCO_3_, 10 mM HEPES, 5.6 mM glucose, 1.1 mM sodium pyruvate as well as penicillin (100 IU/mL) and streptomycin (100 μg/mL), adjusted to pH 7.4 with NaOH. Then, the cauda of epididymis was slightly cut into three pieces, incubated at 37 °C for 10 min in a 5% CO_2_ incubator and the sperm was gently filtered through nylon gauze, centrifuged, and resuspended in 1 mL fresh M199 medium (Gibco). A drop of the sperm suspension was used for assessment of sperm motility and count by CASA. Sperm viability was visualized by eosin and nigrosin staining 24 as aforementioned described in the method section. For examining sperm morphology, a 20-μL sperm suspension was added on a clean glass slide to make a smear, and the slides were air-dried. Sperm morphology was visualized by the Diff-Quick staining according to laboratory manual of WHO for semen morphology 67. A minimum of 100 sperm were counted for each assay.

### RNA extraction and RT-qPCR

Total RNA was extracted from the purified human sperm or rat testis tissues with TRIzol reagent (Life Technologies). Reverse transcription and polymerase chain reaction (PCR) was performed with oligo deoxythymidine (oligo-dT) primers and moloney murine leukemia virus reverse transcriptase (Promega) according to the manufacturer's protocol. PCR primer sequences are listed in table S2. Quantitative real-time PCR (RT-qPCR) assay was performed with GoTaq qPCR Master Mix (Promega) and an ABI 7500 Fast Real-Time PCR Detection System (Applied Biosystems). Briefly, a-20 μL PCR reaction that included 1 μL of complementary DNA, 10 μL of GoTaq qPCR Master Mix, and 0.2 μM of each primer was used and adjusted to the final volume with double distilled H_2_O (ddH_2_O). β-actin in parallel for each run was used as an internal control. The reactions were set up on the basis of the manufacturer's protocol. PCR conditions were incubation at 95 °C for 3 min followed by 40 cycles of thermal cycling (10 s at 95 °C, 20 s at 58 °C, and 10 s at 72 °C). The relative expression ratio of mRNA was quantified via the 2^(ΔΔCt)^ method 75, 76.

### Western blotting

The deposit of human or rat sperm suspension, or a piece of testis tissues of rats were immediately homogenized in ice-cold lysis buffer containing 50 mM Tris (pH 8.0), 150 mM NaCl, 1% NP40, 0.5% sodium deoxycholate, 0.1% SDS, 1 mM phenylmethanesulfonyl fluoride (PMSF). The homogenates were centrifuged at 12, 000 g for 10 min at 4 °C to yield the total protein extract in the supernatant, and then analyzed by Western blotting according to the methods as described elsewhere 77. The concentration of protein was measured with a bicinchoninic acid (BCA) assay kit (Pierce/Thermo Scientific), and an equal amount of protein samples (60 μg) was denatured and then separated through SDS-PAGE using 10% separating gels and transferred to a PVDF membrane (Bio-Rad, Hercules, CA). The membranes were blocked with 5% nonfat milk (or 5% BSA) in TBST (20 mM tris-HCl (pH 7.5), 150 mM NaCl, and 0.05% Tween 20) for 60 min at room temperature and then incubated with the following primary antibodies at 4 °C overnight: rabbit anti-human CatSper1 (1:300, Sigma-Aldrich, Cat# SAB1302217), rabbit anti-human CatSper2 (1:300, Santa Cruz, Cat# sc-98539), rabbit anti-human CatSper3 (1:300, Santa Cruz, Cat# sc-98702), and rabbit anti-human CatSper4 (1:300, Abcam, Cat# ab101892) for human sperm, respectively; rabbit anti-rat CatSper1 (1:100, Santa Cruz, Cat# sc-33153), rabbit anti-rat CatSper2 (1:100, Santa Cruz, Cat# sc-98539), rabbit anti-rat CatSper3 (1:100, Santa Cruz, Cat# sc-98818), and goat anti-rat CatSper4 (1:100, Santa Cruz, Cat# sc-83126) for rats' sperm and testis tissues, respectively, as well as rabbit anti-rat phosphotyrosine (1:500, Abcam, Cat# ab179530), rabbit anti-rat kappa opioid receptor (1:500, Abcam, Cat# ab183825), mouse anti-GAPDH (1:2000, Santa Cruz, Cat# sc-32233), and anti-α-tubulin (1:2000, Cell Signaling Technology, Cat# 3873S). The blots were washed in TBST and then were incubated in horseradish peroxidase-conjugated secondary antibody including goat anti-rabbit IgG-HRP (1:2000, Santa Cruz, Cat# sc-2004), goat anti-mouse IgG-HRP (1:2000, Santa Cruz, Cat# sc-2005), and rabbit anti-goat IgG-HRP (1:2000, Santa Cruz, Cat# sc-2768), respectively. Protein bands were visualized using an enhanced chemiluminescence detection kit (Pierce) followed by autoradiography using Hyperfilm MP (Santa Cruz Biotechnology). The bands were quantified with a computer-assisted imaging analysis system (Image J, NIH).

### Immunofluorescence staining

To prepare epididymal sperm for the immunofluorescence analysis, 20 μL of the sperm suspension was mixed with 20 μL 4% paraformaldehyde (in 0.1 M PB, pH 7.4), then a drop of the mixture was added on a clean glass slide to make a smear, and the slides were air-dried. To prepare testis tissues for the immunofluorescence staining, deeply anesthetized rats were intracardiac perfused with 300 mL of 0.1 M phosphate buffer (PB) followed by 300 mL of 4% paraformaldehyde. The removed testis tissues were post-fixed in 4% paraformaldehyde (in 0.1 M PB, pH 7.4) at 4 °C for 6 h, and were then cryoprotected in 30% sucrose (in 0.1 M PB) at 4 °C. Several days later, the tissues were cut on a cryostat (30 μm thickness) and thaw mounted on gelatin-coated slides for immunostaining processing.

For immunostaining, tissues or sperm were washed three times in PB for 5 min each and blocked in 10% goat or rabbit serum (in 0.1 M PBST) with 0.3% Triton X-100 for 1 h at room temperature, and subsequently were incubated with the respective primary antibody (see table S1) in PBST at 4 °C overnight, which includes rabbit anti-rat CatSper (1:100, Santa Cruz, Cat# sc-33153), rabbit anti-rat CatSper2 (1:100, Santa Cruz, Cat# sc-98539), rabbit anti-rat CatSper3 (1:100, Santa Cruz, Cat# sc-98818), and goat anti-rat CatSper4 (1:100, Santa Cruz, Cat# sc-83126), respectively. Then, after three washes in PBS, tissues or sperm were incubated with the following appropriate secondary antibodies at room temperature for 1 h: fluorescein-5-isothiocyanate (FITC)-labeled goat anti-rabbit IgG (1:200, ZSGB-BIO, Cat# ZF-0511), FITC-labeled rabbit anti-goat IgG (1:200, ZSGB-BIO, Cat# ZF-0514) and Alexa Fluor 647 goat anti-rabbit IgG (1:200, YEASEN, Cat# 33113ES60). The tissues or sperm were counterstained with the nuclear marker DAPI (100 ng/mL) carrying blue fluorescence for 10 min at room temperature. The slides were mounted in Gel-Mount medium. Visualization of fluorescence signal was performed by confocal microscopy at excitation wavelengths of 488 nm (green), 543 nm (red), and 405 and 647 nm (blue), respectively. At least four fields per slide were analyzed to establish reproducibility.

### Calcium imaging analysis

Sperm calcium imaging was performed as previously described 78, 79. Briefly, human sperm were purified by the discontinuous density gradient procedure as aforementioned described in the method section, washed in HTF medium containing (in mM): 97.8 NaCl, 4.69 KCl, 4 NaHCO_3_, 0.37 KH_2_PO_4_, 2.04 CaCl_2_, 0.2 MgCl_2_, 21.4 lactic acid, 21 HEPES, 2.78 glucose, and 0.33 Na-pyruvate, pH 7.3 (adjusted with NaOH), and re-suspended in HTF containing 3 mg/mL human serum albumin (HTF^+^ medium). Rat sperm were isolated from the cauda epididymis by swim-out in HS solution containing (in mM): 135 NaCl, 5 KCl, 1 MgSO_4_, 2 CaCl_2_, 20 HEPES, 5 glucose, 10 lactic acid, and 1 Na-pyruvate, pH 7.4 (adjusted with NaOH), washed and re-suspended in HS fortified with 5 mg/mL bovine serum albumin (HS^+^ solution). Then, human sperm were loaded with 5 μM Fura-2 AM (Molecular Probes, USA) in the presence of 0.05% pluronic F-127 (Molecular Probes, USA), while rat sperm were loaded with 10 μM Fluo-4 AM (Molecular Probes, USA) and 0.05% pluronic F-127 (Molecular Probes, USA), at 37 °C for 30 min in a 5% CO_2_ incubator in the dark, followed by washing in HTF or HS medium. The washed sperm were loaded on Cell-Take (BD™ Biosciences, USA) coated coverslips of glass bottom cell culture dishes (diameter 1.5 cm, Nest Biotechnology Co., Ltd.), and were allowed to attach for 20 min.

For human sperm imaging, a monochromator (Polychrome V, TILL Photonics GmbH) was used to generate an excitation at 340 nm for Fura-2. A 100× objective on an inverted microscope (IX-71, Olympus) was used for imaging. Emissions (515-565 nm) were bandpass filtered (HQ540/50, Chroma) and collected with cooled CCD camera (CoolSNAP HQ, Roper Scientific) that recorded 100 ms every 5 s. For rat sperm imaging, a monochromator was used to generate an excitation at 480 nm for Fluo-4. A 40× objective on an inverted microscope (Leica TCS SP8) was used for imaging and the images were recorded 100 ms every 5 s.

Fluorescence was monitored before and after application of NH_4_Cl (30 mM). Changes in Fura-2 fluorescence are presented as the ratio of F340/F380 after background subtraction 80. Normalized F340/F380 = F_treatment_/F_baseline_ or F_before_/F_baseline_, in which F_baseline_ indicates the mean basal fluorescence intensity of sperm before NH_4_Cl treatment, F_before_ represents the fluorescence intensity of sperm before NH_4_Cl treatment, and F_treatment_ indicates the fluorescence of sperm after NH_4_Cl treatment. Changes in Fluo-4 fluorescence are depicted as ΔF/F0 (%), that is, the change in fluorescence (ΔF) relative to the mean basal fluorescence (F0) before NH_4_Cl treatment, where ΔF = F-F0, F0 indicates the mean basal fluorescence intensity of sperm before NH_4_Cl treatment, and F represents the fluorescence intensity of sperm before or after NH_4_Cl treatment. The images were analyzed using commercial software (MetaFluor v7, Molecular Devices for human sperm or Leica LAS X 3.0 for rat sperm).

### Assessment of hyperactivation and acrosome reaction

For assessing the protein tyrosine phosphorylation, hyperactivation, and NH_4_Cl induced-acrosome reaction of rat sperm, the sperm were first capacitated in HS^++^ solution (HS^+^ solution added with 15 mM NaHCO_3_ and 30 mM NH_4_Cl) at 37 °C for 90 min in a 5% CO_2_ incubator, then CASA was applied to evaluate the sperm motility. Hyperactivation of sperm was defined as follows 81: VCL > 100 μm/s; ALH ≥ 2.0 μm; LIN ≤ 38.0%, and WOB ≥ 16%.

Acrosome reaction of sperm was assessed by the method described elsewhere 82. Briefly, after capacitation, the sperm were then pelleted and washed twice, spread onto slides, air dried, and fixed with 4% formaldehyde. The acrosomes were stained using 1 μM of arachis hypogaea (peanut) agglutinin (fluorescein isothiocyanate-conjugated peanut agglutinin (PNA-FITC); Sigma), and the DNA was counterstained with 4ʹ,6-diamidino-2-phenylindole (DAPI; Sigma) as previously described 82. Acrosome reaction is manifested as very weak or absent fluorescence, which is characteristic of acrosome-reacted sperm. The stained sperm were examined using a confocal laser scanning microscope (Leica TCS SP8). A minimal of 200 spermatozoa were counted to assess hyperactivation and acrosome reaction of sperm.

### *In vivo* fertility assay

Male rats were mated with 6-week-old female rats at a 1:2 ratio,* i.e.*, one male rat was caged with two female rats overnight, and vaginal smears of the females were taken at the following morning and examined under a microscope. Female rats whose smears were positive for spermatozoa were considered to have successfully mated, and these female rats were then housed individually 82. After birth, the pup numbers per litter and the pregnancy rate of female rats were recorded and analyzed.

### Plasmid construction, lentivirus infection, and drug administration

Construction and production of both recombinant lentivirus-expressing shRNA targeting rat CatSper1 coupled to ZsGreen or mCherry tag (LV-shCatSper1) or lentivirus-expressing CatSper1 linked with ZsGreen tag (LV-CatSper1) were completed by Likeli Technologies (Beijing, China) using either pLVX-mCMV-ZsGreen or pLVX-mCMV-mCherry vector. Three nucleotide sequences of CatSper1 shRNA are listed in table S3. The preliminary experiments showed that the shRNA-3 targeting rat CatSper1 exerted a significant effect for silencing CatSper1 gene in the testis tissues. Hence, the shRNA-3 was chosen for the present study.

For the knockdown of CatSper1 in the testis tissues, the LV-shCatSper1 lentivirus (or vector control) was in situ injected into the rat testis tissues at a final titer of 5 × 10^8^ transducing units/mL (in a 5-μL volume of solution per point, 5 points for each side, total volume of 50 μL in both left and right side of the testis tissues). 14 days after viral infection, a transfection efficiency of lentivirus was routinely achieved as observed under fluorescence microscopy. Knockdown of CatSper1 in the testis tissues was performed either in normal rats or in 2 Hz EA-treated AZS rats. In EA-treated AZS rats, the LV-shCatSper1 lentivirus was delivered to rats on day 11 after ORN administration, and then the EA treatment was carried out to rats as aforementioned described in the method section.

For the overexpression of CatSper1 in the testis tissues, the LV-CatSper1 lentivirus (or vector control) was *in situ* injected into the rat testis tissues in the same dose and manner as the LV-shCatSper1 lentivirus injection. Likewise, a transfection efficiency of lentivirus was routinely achieved as observed under fluorescence microscopy at 14 days after viral infection. Overexpression of CatSper1 in the testis tissues was performed either in AZS rats or in CatSper1 knockdown rats. In AZS rats, the LV-CatSper1 lentivirus was delivered to rats on day 11 after ORN administration, while in CatSper1 knockdown rats, the LV-CatSper1 lentivirus was delivered to rats on day 11 after the LV-shCatSper1 lentivirus injection.

For the inhibition of* kappa* opioid receptor activity in the testis tissues of 100 Hz EA-treated AZS rats. Normal saline or *kappa* opioid receptor antagonist (nor-Binaltorphimine, 1 mM) was in situ injected into the rat testis tissues (in a 5-μL volume of solution per point, 5 points for each side, total volume of 50 μL in both left and right side of the testis tissues) once per day from day 11 to day 23 after ORN administration (i.e. before and during EA treatment), and then 100 Hz-EA or mock-EA treatment was carried out to ORN-induced AZS rats as aforementioned described in the method section (Electroacupuncture treatment for animals).

### Statistical analysis

Statistical analyses were performed with GraphPad Prism 8.0 for Windows (GraphPad Software, La Jolla, CA). All quantitative biochemical data and immunofluorescence staining were representative of at least three independent experiments. For statistical comparisons, all data were first subjected to a Gaussian distribution test, and only the data were normally distributed and variances were similar between groups to be compared were subjected to parametric statistical tests. Two-tailed unpaired Student's *t* test was used for the comparison of the mean values between two groups. One-way ANOVA with Sidak's *post-hoc* test was used for multiple comparisons. All data were expressed as means ± SEM, and differences with P < 0.05 were considered statistically significant. The significant differences between groups were represented as ^*^P < 0.05, ^**^P < 0.01, and ^***^P < 0.001. All statistical data are presented in Supplementary table S4.

## Supplementary Material

Supplementary figures, tables 1-3, and video legends.

Supplementary table 4.

Supplementary video 1.

Supplementary video 2.

Supplementary video 3.

Supplementary video 4.

Supplementary video 5.

Supplementary video 6.

## Abbreviations

AZS: asthenozoospermic
AR: acrosome reaction
ALH: amplitude of lateral head displacement
CatSper: cation channel of sperm
CMC-Na: carboxymethylcellulose sodium
CASA: computer-assisted semen analysis
DAPI: 4ʹ,6-diamidino-2-phenylindole
EA: Electroacupuncture
HTF: human tubal fluid
I_CatSper_: CatSper current
LIN: linearity
NP: non-progressive motility
Nor-bin: Nor-Binaltorphimine
ORN: ornidazole
PNA-FITC: fluorescein isothiocyanate-conjugated peanut agglutinin
PR: progressive motility
pTyr: protein tyrosine phosphorylation
RT-qPCR: quantitative real-time polymerase chain reaction
STR: straightness
SJS: Sheng-Jing-San
TEAS: transcutaneous electrical acupoint stimulation
TCM: traditional Chinese medicine
VSL: straight-line velocity
VCL: curve-line velocity
VAP: average path velocity
WOB: wobble

## References

[1] Bracke A, Peeters K, Punjabi U, Hoogewijs D, Dewilde S (2018). A search for molecular mechanisms underlying male idiopathic infertility. Reprod Biomed Online.

[2] Brown SG, Publicover SJ, Barratt CLR, Martins da Silva SJ (2019). Human sperm ion channel (dys)function: implications for fertilization. Hum Reprod Update.

[3] Lishko PV, Mannowetz N (2018). CatSper: A Unique Calcium Channel of the Sperm Flagellum. Curr Opin Physiol.

[4] Ren D, Navarro B, Perez G, Jackson AC, Hsu S, Shi Q (2001). A sperm ion channel required for sperm motility and male fertility. Nature.

[5] Carlson AE, Westenbroek RE, Quill T, Ren D, Clapham DE, Hille B (2003). CatSper1 required for evoked Ca2+ entry and control of flagellar function in sperm. Proc Natl Acad Sci U S A.

[6] Jin J, Jin N, Zheng H, Ro S, Tafolla D, Sanders KM (2007). Catsper3 and Catsper4 are essential for sperm hyperactivated motility and male fertility in the mouse. Biol Reprod.

[7] Qi H, Moran MM, Navarro B, Chong JA, Krapivinsky G, Krapivinsky L (2007). All four CatSper ion channel proteins are required for male fertility and sperm cell hyperactivated motility. Proc Natl Acad Sci U S A.

[8] Quill TA, Sugden SA, Rossi KL, Doolittle LK, Hammer RE, Garbers DL (2003). Hyperactivated sperm motility driven by CatSper2 is required for fertilization. Proc Natl Acad Sci U S A.

[9] Luo T, Chen HY, Zou QX, Wang T, Cheng YM, Wang HF (2019). A novel copy number variation in CATSPER2 causes idiopathic male infertility with normal semen parameters. Hum Reprod.

[10] Avenarius MR, Hildebrand MS, Zhang Y, Meyer NC, Smith LL, Kahrizi K (2009). Human male infertility caused by mutations in the CATSPER1 channel protein. Am J Hum Genet.

[11] Brown SG, Miller MR, Lishko PV, Lester DH, Publicover SJ, Barratt CLR (2018). Homozygous in-frame deletion in CATSPERE in a man producing spermatozoa with loss of CatSper function and compromised fertilizing capacity. Hum Reprod.

[12] Hildebrand MS, Avenarius MR, Fellous M, Zhang Y, Meyer NC, Auer J (2010). Genetic male infertility and mutation of CATSPER ion channels. Eur J Hum Genet.

[13] Smith JF, Syritsyna O, Fellous M, Serres C, Mannowetz N, Kirichok Y (2013). Disruption of the principal, progesterone-activated sperm Ca2+ channel in a CatSper2-deficient infertile patient. Proc Natl Acad Sci U S A.

[14] Hwang JY, Mannowetz N, Zhang Y, Everley RA, Gygi SP, Bewersdorf J (2019). Dual Sensing of Physiologic pH and Calcium by EFCAB9 Regulates Sperm Motility. Cell.

[15] Carlson AE, Quill TA, Westenbroek RE, Schuh SM, Hille B, Babcock DF (2005). Identical phenotypes of CatSper1 and CatSper2 null sperm. J Biol Chem.

[16] Marquez B, Ignotz G, Suarez SS (2007). Contributions of extracellular and intracellular Ca2+ to regulation of sperm motility: Release of intracellular stores can hyperactivate CatSper1 and CatSper2 null sperm. Dev Biol.

[17] Lobley A, Pierron V, Reynolds L, Allen L, Michalovich D (2003). Identification of human and mouse CatSper3 and CatSper4 genes: characterisation of a common interaction domain and evidence for expression in testis. Reprod Biol Endocrinol.

[18] Jin JL, O'Doherty AM, Wang S, Zheng H, Sanders KM, Yan W (2005). Catsper3 and catsper4 encode two cation channel-like proteins exclusively expressed in the testis. Biol Reprod.

[19] Nikpoor P, Mowla SJ, Movahedin M, Ziaee SA, Tiraihi T (2004). CatSper gene expression in postnatal development of mouse testis and in subfertile men with deficient sperm motility. Hum Reprod.

[20] Carkci S, Etem EO, Ozaydin S, Karakeci A, Tektemur A, Ozan T (2017). Ion channel gene expressions in infertile men: A case-control study. Int J Reprod Biomed (Yazd).

[21] Li HG, Ding XF, Liao AH, Kong XB, Xiong CL (2007). Expression of CatSper family transcripts in the mouse testis during post-natal development and human ejaculated spermatozoa: relationship to sperm motility. Mol Hum Reprod.

[22] Bhilawadikar R, Zaveri K, Mukadam L, Naik S, Kamble K, Modi D (2013). Levels of Tektin 2 and CatSper 2 in normozoospermic and oligoasthenozoospermic men and its association with motility, fertilization rate, embryo quality and pregnancy rate. J Assist Reprod Genet.

[23] Tamburrino L, Marchiani S, Minetti F, Forti G, Muratori M, Baldi E (2014). The CatSper calcium channel in human sperm: relation with motility and involvement in progesterone-induced acrosome reaction. Hum Reprod.

[24] Wang YN, Wang B, Liang M, Han CY, Zhang B, Cai J (2013). Down-regulation of CatSper1 channel in epididymal spermatozoa contributes to the pathogenesis of asthenozoospermia, whereas up-regulation of the channel by Sheng-Jing-San treatment improves the sperm motility of asthenozoospermia in rats. Fertil Steril.

[25] Shahrokhi SZ, Salehi P, Alyasin A, Taghiyar S, Deemeh MR (2020). Asthenozoospermia: Cellular and molecular contributing factors and treatment strategies. Andrologia.

[26] Dieterle S, Li C, Greb R, Bartzsch F, Hatzmann W, Huang D (2009). A prospective randomized placebo-controlled study of the effect of acupuncture in infertile patients with severe oligoasthenozoospermia. Fertil Steril.

[27] Djaali W, Abdurrohim K, Helianthi DR (2019). Management of Acupuncture as Adjuvant Therapy for *In Vitro* Fertilization. Med Acupunct.

[28] You F, Ruan L, Zeng L, Zhang Y (2020). Efficacy and safety of acupuncture for the treatment of oligoasthenozoospermia: A systematic review. Andrologia.

[29] Siterman S, Eltes F, Wolfson V, Zabludovsky N, Bartoov B (1997). Effect of acupuncture on sperm parameters of males suffering from subfertility related to low sperm quality. Arch Androl.

[30] Yu Y, Sha SB, Zhang B, Guan Q, Liang M, Zhao LG (2019). Effects and mechanism of action of transcutaneous electrical acupuncture point stimulation in patients with abnormal semen parameters. Acupunct Med.

[31] Zhu J, Arsovska B, Kozovska K (2018). Acupuncture Treatment for Fertility. Open Access Maced J Med Sci.

[32] Jin ZR, Liu BH, Tang WH, Jiang H, Zhang R, Han JS (2017). Transcutaneous electrical acupoint stimulation for asthenozoospermia. Zhonghua Nan Ke Xue.

[33] Jin ZR, Liu BH, Cai J, Jing XH, Zhu B, Xing GG (2017). Experimental Study for the Treatment of Asthenozoospermia by Electroacupuncture in Rats. Zhen Ci Yan Jiu.

[34] Bone W, Jones AR, Cooper TG (2002). The effect of (R,S)-ornidazole on the fertility of male mice and the excretion and metabolism of 36Cl-(R,S)-ornidazole and 36Cl-(R,S)-alpha-chlorohydrin in male mice and rats. Int J Androl.

[35] Oberlander G, Yeung CH, Cooper TG (1994). Induction of reversible infertility in male rats by oral ornidazole and its effects on sperm motility and epididymal secretions. J Reprod Fertil.

[36] Oberländer G, Yeung CH, Cooper TG (1996). Influence of oral administration of ornidazole on capacitation and the activity of some glycolytic enzymes of rat spermatozoa. J Reprod Fertil.

[37] Du Y, Liu H, Zhang M, Zhang S, Hu J, Wu G (2019). Taurine Increases Spermatozoa Quality and Function in Asthenospermia Rats Impaired by Ornidazole. Adv Exp Med Biol.

[38] Sun Y, Sun X, Zhao L, Zhang Z, Wang Y, Dai Z (2020). DJ-1 deficiency causes metabolic abnormality in ornidazole-induced asthenozoospermia. Reproduction.

[39] Sun XH, Zhu YY, Wang L, Liu HL, Ling Y, Li ZL (2017). The Catsper channel and its roles in male fertility: a systematic review. Reprod Biol Endocrinol.

[40] Ho K, Wolff CA, Suarez SS (2009). CatSper-null mutant spermatozoa are unable to ascend beyond the oviductal reservoir. Reprod Fertil Dev.

[41] Wang H, Liu J, Cho KH, Ren D (2009). A novel, single, transmembrane protein CATSPERG is associated with CATSPER1 channel protein. Biol Reprod.

[42] Singh AP, Rajender S (2015). CatSper channel, sperm function and male fertility. Reprod Biomed Online.

[43] Zhang Z, Wang GL, Li HX, Li L, Cui QW, Wei CB (2012). Regulation of fertilization in male rats by CatSper2 knockdown. Asian J Androl.

[44] Yu Q, Mei XQ, Ding XF, Dong TT, Dong WW, Li HG (2015). Construction of a catsper1 DNA vaccine and its antifertility effect on male mice. PLoS One.

[45] Fu B, Lun X, Gong Y (2005). Effects of the combined therapy of acupuncture with herbal drugs on male immune infertility-a clinical report of 50 cases. J Tradit Chin Med.

[46] Bidouee F, Shamsa A, Jalali M (2011). Effect of acupuncture on azoospermic male. Saudi J Kidney Dis Transpl.

[47] Peng S, Zheng Y, Zheng K, Lin K, Wu J, Zheng W (2014). Effect of a comprehensive therapy plus gushenyutai plaster administered at guanyuan (CV 4) on male infertility associated with semen non-liquefaction. J Tradit Chin Med.

[48] Fischl F, Riegler R, Bieglmayer C, Nasr F, Neumark J (1984). Modification of semen quality by acupuncture in subfertile males. Geburtshilfe Frauenheilkd.

[49] Siterman S, Eltes F, Wolfson V, Lederman H, Bartoov B (2000). Does acupuncture treatment affect sperm density in males with very low sperm count? A pilot study. Andrologia.

[50] Pei J, Strehler E, Noss U, Abt M, Piomboni P, Baccetti B (2005). Quantitative evaluation of spermatozoa ultrastructure after acupuncture treatment for idiopathic male infertility. Fertil Steril.

[51] Jo J, Kang MJ (2016). Successful Treatment of Oligoasthenozoospermia Using Traditional Korean Medicine Resulting in Spontaneous Pregnancy: Two Case Reports. Explore (NY).

[52] Zhao Y, Zhang P, Ge W, Feng Y, Li L, Sun Z (2020). Alginate oligosaccharides improve germ cell development and testicular microenvironment to rescue busulfan disrupted spermatogenesis. Theranostics.

[53] Mohammadi S, Movahedin M, Mowla SJ (2009). Up-regulation of CatSper genes family by selenium. Reprod Biol Endocrinol.

[54] Mohammadi S, Jalali M, Nikravesh MR, Fazel A, Ebrahimzadeh A, Gholamin M (2013). Effects of Vitamin-E treatment on CatSper genes expression and sperm quality in the testis of the aging mouse. Iran J Reprod Med.

[55] Park EH, Kim do R, Kim HY, Park SK, Chang MS (2014). Panax ginseng induces the expression of CatSper genes and sperm hyperactivation. Asian J Androl.

[56] Han JS (2003). Acupuncture: neuropeptide release produced by electrical stimulation of different frequencies. Trends Neurosci.

[57] Huo R, Han SP, Liu FY, Shou XJ, Liu LY, Song TJ (2020). Responses of Primary Afferent Fibers to Acupuncture-Like Peripheral Stimulation at Different Frequencies: Characterization by Single-Unit Recording in Rats. Neurosci Bull.

[58] Agirregoitia E, Valdivia A, Carracedo A, Casis L, Gil J, Subiran N (2006). Expression and localization of delta-, kappa-, and mu-opioid receptors in human spermatozoa and implications for sperm motility. J Clin Endocrinol Metab.

[59] Albrizio M, Guaricci AC, Calamita G, Zarrilli A, Minoia P (2006). Expression and immunolocalization of the mu-opioid receptor in human sperm cells. Fertil Steril.

[60] Kilpatrick DL, Borland K, Jin DF (1987). Differential expression of opioid peptide genes by testicular germ cells and somatic cells. Proc Natl Acad Sci U S A.

[61] Estomba H, Munoa-Hoyos I, Gianzo M, Urizar-Arenaza I, Casis L, Irazusta J (2016). Expression and Localization of Opioid Receptors in Male Germ Cells and the Implication for Mouse Spermatogenesis. PLoS One.

[62] Tian M, Broxmeyer HE, Fan Y, Lai Z, Zhang S, Aronica S (1997). Altered hematopoiesis, behavior, and sexual function in mu opioid receptor-deficient mice. J Exp Med.

[63] Vicente-Carrillo A, Alvarez-Rodriguez M, Rodriguez-Martinez H (2016). The mu (mu) and delta (delta) opioid receptors modulate boar sperm motility. Mol Reprod Dev.

[64] Jerng UM, Jo JY, Lee S, Lee JM, Kwon O (2014). The effectiveness and safety of acupuncture for poor semen quality in infertile males: a systematic review and meta-analysis. Asian J Androl.

[65] Zhu X, Yang L, Li Z, Pan Z, Huang S, Xiong Y (2020). Safety and effectiveness of acupuncture for POSEIDON patients in IVF/ICSI: A protocol for systematic review and meta-analysis. Medicine (Baltimore).

[66] Wang J, Han L, Bao B, Yu X, Zhang K, Dai H (2019). The safety and efficacy of acupuncture for epididymitis protocol for a systematic review. Medicine (Baltimore).

[67] World Health Organization (2010). WHO laboratory manual for the examination and processing of human semen, 5th edn. Geneva: World Health Organization.

[68] Qu F, Li R, Sun W, Lin G, Zhang R, Yang J (2017). Use of electroacupuncture and transcutaneous electrical acupoint stimulation in reproductive medicine: a group consensus. J Zhejiang Univ Sci B.

[69] Siterman S, Eltes F, Schechter L, Maimon Y, Lederman H, Bartoov B (2009). Success of acupuncture treatment in patients with initially low sperm output is associated with a decrease in scrotal skin temperature. Asian J Androl.

[70] Gao J, Zuo Y, So KH, Yeung WS, Ng EH, Lee KF (2012). Electroacupuncture enhances spermatogenesis in rats after scrotal heat treatment. Spermatogenesis.

[71] Kelly MC, Brown SG, Costello SM, Ramalingam M, Drew E, Publicover SJ (2018). Single-cell analysis of [Ca2+]i signalling in sub-fertile men: characteristics and relation to fertilization outcome. Hum Reprod.

[72] Ribas-Maynou J, Fernandez-Encinas A, Garcia-Peiro A, Prada E, Abad C, Amengual MJ (2014). Human semen cryopreservation: a sperm DNA fragmentation study with alkaline and neutral Comet assay. Andrology.

[73] Garcia-Peiro A, Oliver-Bonet M, Navarro J, Abad C, Amengual MJ, Lopez-Fernandez C (2012). Differential clustering of sperm subpopulations in infertile males with clinical varicocele and carriers of rearranged genomes. J Androl.

[74] Zhao H-Y, Liu L-Y, Cai J, Cui Y-J, Xing G-G (2018). Electroacupuncture Treatment Alleviates the Remifentanil-Induced Hyperalgesia by Regulating the Activities of the Ventral Posterior Lateral Nucleus of the Thalamus Neurons in Rats. Neural plasticity.

[75] Fang D, Kong LY, Cai J, Li S, Liu XD, Han JS (2015). Interleukin-6-mediated functional upregulation of TRPV1 receptors in dorsal root ganglion neurons through the activation of JAK/PI3K signaling pathway: roles in the development of bone cancer pain in a rat model. Pain.

[76] Xu S, Xu Y, Yin M, Zhang S, Liu P, Koroleva M (2018). Flow-dependent epigenetic regulation of IGFBP5 expression by H3K27me3 contributes to endothelial anti-inflammatory effects. Theranostics.

[77] Lin SC, Lee HC, Hsu CT, Huang YH, Li WN, Hsu PL (2019). Targeting Anthrax Toxin Receptor 2 Ameliorates Endometriosis Progression. Theranostics.

[78] Luo T, Zou QX, He YQ, Wang HF, Li N, Zeng XH (2015). Matrine inhibits mouse sperm function by reducing sperm [Ca2+]i and phospho-ERK1/2. Cell Physiol Biochem.

[79] Rennhack A, Schiffer C, Brenker C, Fridman D, Nitao ET, Cheng YM (2018). A novel cross-species inhibitor to study the function of CatSper Ca(2+) channels in sperm. Br J Pharmacol.

[80] Yin C, Liu B, Li Y, Li X, Wang J, Chen R (2020). IL-33/ST2 induces neutrophil-dependent reactive oxygen species production and mediates gout pain. Theranostics.

[81] Aguirre-Arias MV, Velarde V, Moreno RD (2017). Effects of ascorbic acid on spermatogenesis and sperm parameters in diabetic rats. Cell Tissue Res.

[82] Wang T, Yin Q, Ma X, Tong MH, Zhou Y (2018). Ccdc87 is critical for sperm function and male fertility. Biol Reprod.

