# Supplementary material for: Roles of CatSper channels in the pathogenesis of asthenozoospermia and the therapeutic effects of acupuncture-like treatment on asthenozoospermia
Source: Theranostics. 2021 Jan 1;11(6):2822–44. doi: 10.7150/thno.51869 (PMC7806476; doi:10.7150/thno.51869)
Supplement: Supplementary file 1 — Supplementary figures, tables 1-3, and video legends. [file thnov11p2822s1.pdf]

Supplementary File for

Roles of CatSper channels in the pathogenesis of asthenozoospermia and the therapeutic effects of acupuncture-like treatment on asthenozoospermia

Zi-Run Jin, Dong Fang, Bo-Heng Liu, Jie Cai, Wen-Hao Tang, Hui Jiang, and Guo-Gang Xing

Inventory of Supplementary Information

Figures S1-S22 and Figure Legends

Figure S1. The effective rate of TEAS treatment to idiopathic asthenozoospermic (iAZS) patients at frequencies of 2 Hz and 100 Hz and the effects of 100 Hz-TEAS treatment on CatSper1 and CatSper3 protein expression in the sperm of iAZS patients. Related to Figure 2.

Figure S2. Reduction of the sperm motility, sperm viability and the mean fluorescence intensity of CatSper1 to CatSper4 in the epididymal sperm of asthenozoospermic (AZS) model rats. Related to Figure 3.

Figure S3. Reduction of CatSper (from CatSper1 to CatSper4) mRNA and protein abundance in the testis tissues of asthenozoospermic (AZS) model rats. Related to Figure 3.

Figure S4. Validation of the decreased sperm motility of the ORN-induced asthenozoospermic (AZS) male rats that were used to mate with the normal female rats in the *in vivo* fertility assay experiments. Related to Figure 3.

Figure S5. Validation of the lentivirus infection efficiency by immunofluorescence staining. Related to Figure 4.

Figure S6. Reduction of CatSper1 immunofluorescence staining and impairment of sperm motility in the epididymal sperm of CatSper1 knockdown (KD) rats. Related to Figure 4.

Figure S7. Immunofluorescence staining with fluo-4 and mCherry in the epididymal sperm of rats received LV-shCatSper1 or LV-mCherry injection. Related to Figure 4.

Figure S8. Validation of the decreased sperm motility of the CatSper1 knockdown (KD) rats that were used to mate with the normal female rats in the *in vivo* fertility assay experiments. Related to Figure 4.

Figure S9. CatSper1 overexpression (OE) rescues the impaired sperm motility of CatSper1 knockdown (KD) rats. Related to Figure 5.

Figure S10. Validation of the improved sperm motility of the CatSper1 knockdown (KD) rats with CatSper1 overexpression (OE) in the *in vivo* fertility assay experiments. Related to Figure 5.

Figure S11. CatSper1 overexpression cannot rescue the impaired sperm motility and functional characteristics of asthenozoospermic (AZS) model rats. Related to Figure 5.

Figure S12. Determination of the protocols for 2 Hz-EA treatment on the sperm motility of asthenozoospermic (AZS) model rats. Related to Figure 6.

Figure S13. Improved sperm motility and viability of asthenozoospermic (AZS) rats with 2 Hz-EA treatment, by the protocol of once every other day for five times. Related to Figure 6.

Figure S14. Enhancement of CatSper mRNA and protein (from CatSper1 to CatSper4) abundance in the testis tissues of asthenozoospermic (AZS) rats with 2 Hz-EA treatment, by the protocol of once every other day for five times. Related to Figure 6.

Figure S15. Validation of improved sperm motility of 2 Hz EA-treated asthenozoospermic (AZS) male rats that were used to mate with the normal female rats in the *in vivo* fertility assay experiments. Related to Figure 6.

Figure S16. CatSper1 knockdown abolishes the 2 Hz EA-induced improvement of sperm motility to asthenozoospermic (AZS) rats. Related to Figure 7.

Figure S17. Immunofluorescence staining with fluo-4 and mCherry in the epididymal sperm of 2 Hz EA-treated asthenozoospermic (AZS) rats infected with LV-shCatSper1 or LV-mCherry. Related to Figure 7.

Figure S18. Validation of impaired sperm motility of 2 Hz EA-treated asthenozoospermic male rats by CatSper1 knockdown (KD) in the *in vivo* fertility assay experiments. Related to Figure 7.

Figure S19. Effects of 100 Hz-EA treatment (by the protocol of once every other day for five times) on the sperm motility and viability of asthenozoospermic (AZS) model rats. Related to Discussion.

Figure S20. Effects of 100 Hz-EA treatment (by the protocol of once every other day for five times) on CatSper protein (from CatSper1 to CatSper4) expression in the sperm of asthenozoospermic (AZS) model rats. Related to Discussion.

Figure S21. Effects of 100 Hz-EA treatment (by the protocol of once every other day for five times) on kappa opioid receptor protein expression in the sperm of asthenozoospermic (AZS) model rats. Related to Discussion.

Figure S22. Effects of kappa opioid receptor (KOR) antagonist norbinaltorphimine (Nor-BNI) on the improved sperm motility of asthenozoospermic (AZS) model rats with 100 Hz EA treatment. Related to Discussion.

Supplementary Tables S1-S3 and Legends

Table S1. Key resources table. Related to Material and Methods.

Table S2. PCR primer sequences. Related to Material and Methods.

Table S3. CatSper1 shRNA nucleotide sequences. Related to Material and Methods.

Supplementary Video S1-Video S6 and Legends

Supplementary Video S1. Calcium imaging recorded from the sperm of healthy subjects and idiopathic asthenozoospermic (iAZS) patients, and in the presence or absence of CatSper inhibitor NNC. Related to Fig. 1H.

Supplementary Video S2. Calcium imaging recorded from the sperm of idiopathic asthenozoospermic (iAZS) patients with mock-TEAS or 2 Hz-TEAS treatment. Related to Fig. 2I-2J.

Supplementary Video S3. Computer-assisted semen analysis (CASA) and calcium imaging for the epididymal sperm of asthenozoospermic (AZS) model rats. Related to Fig. 3 and Figure S2E-2J.

Supplementary Video S4. Computer-assisted semen analysis (CASA) and calcium imaging for the epididymal sperm of CatSper1 knockdown (KD) rats. Related to Fig. 4 and Figure S6E-6J.

Supplementary Video S5. Computer-assisted semen analysis (CASA) and calcium imaging for the epididymal sperm of asthenozoospermic (AZS) rats with 2 Hz-EA or mock-EA treatment. Related to Fig. 6 and Figure S13.

Supplementary Video S6. Computer-assisted semen analysis (CASA) and calcium imaging for the epididymal sperm of 2 Hz EA-treated AZS rats with CatSper1 knockdown (KD). Related to Fig. 7 and Figure S16E-J.

Figures S1-S22 and Figure Legends

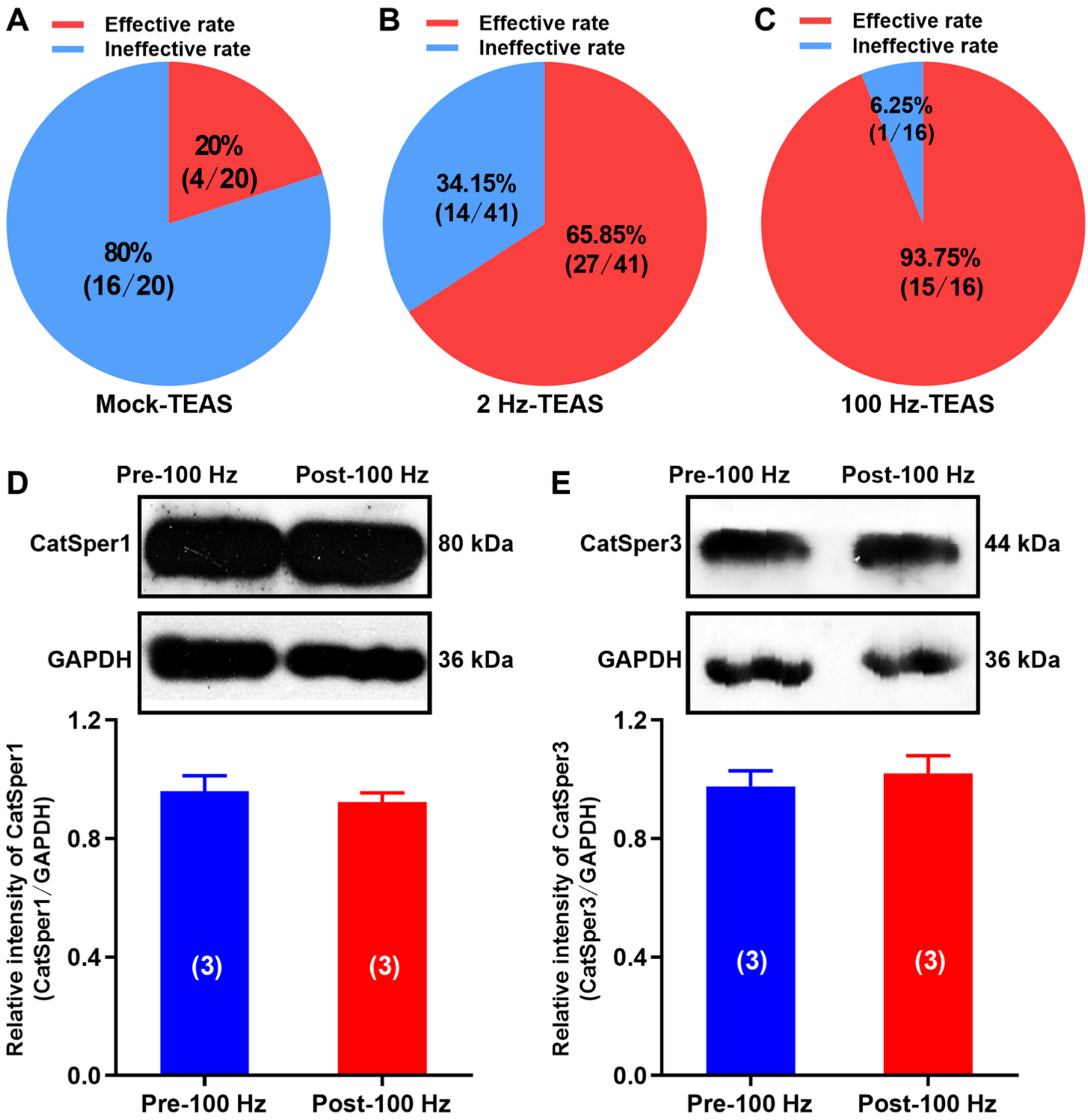

Figure S1. The effective rate of TEAS treatment to idiopathic asthenozoospermic (iAZS) patients at frequencies of 2 Hz and 100 Hz and the effects of 100 Hz-TEAS treatment on CatSper1 and CatSper3 protein expression in the sperm of iAZS patients. Related to Fig. 2. (A-C) The effective rate of mock-TEAS (A), 2 Hz-TEAS (B) and 100 Hz-TEAS (C) treatment to iAZS patients. n = 16-41 iAZS patients per group. (D-E) Effects of 100 Hz-TEAS treatment on expression of CatSper1 and CatSper3 protein in the sperm of iAZS patients (n = 3 tested subjects per group). All data are presented as mean ± SEM. $t_{(4)} = 0.61$, P = 0.5750 for D, and $t_{(4)} = 0.54$, P = 0.6180 for E, Unpaired *t* test.

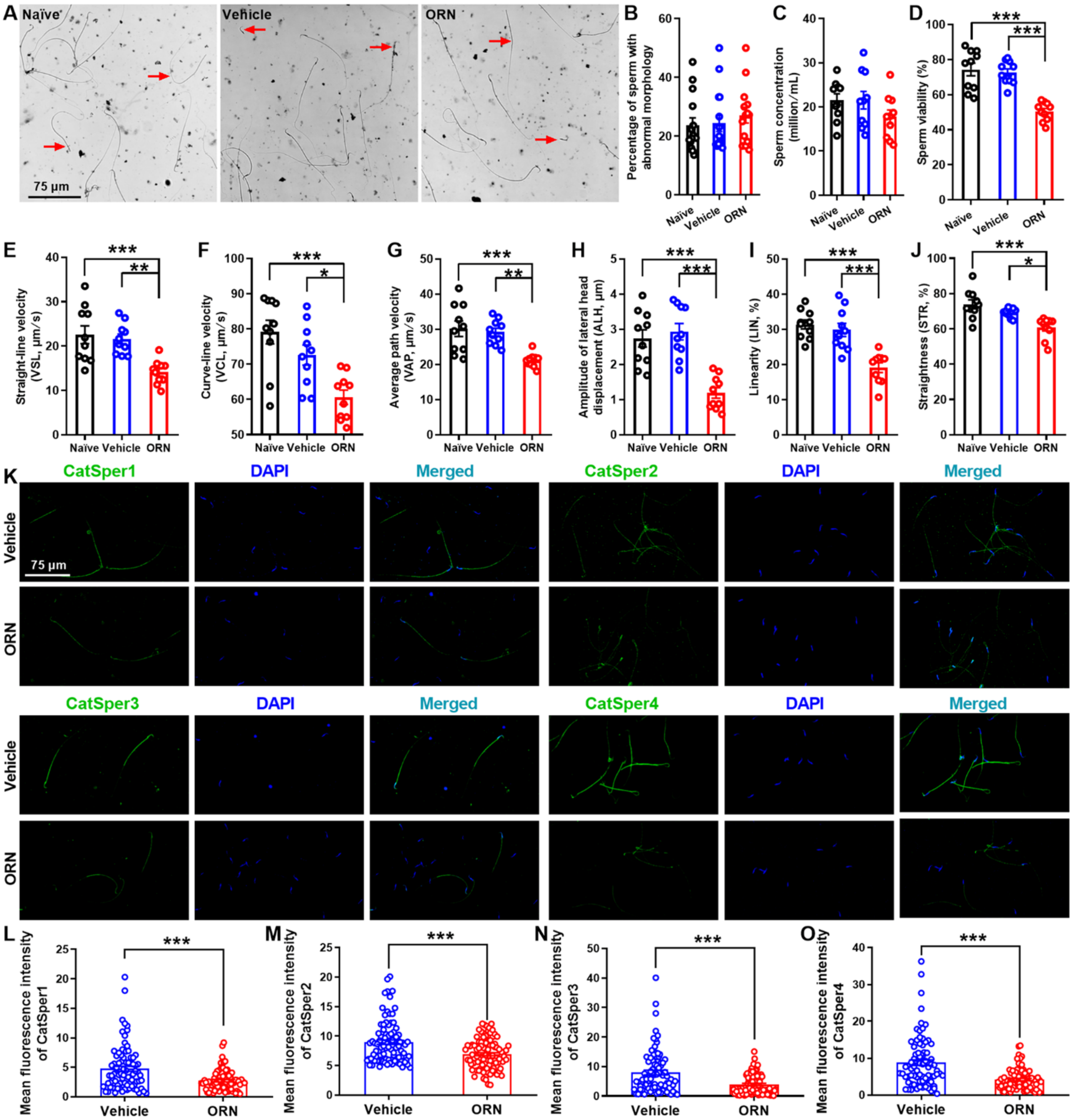

Figure S2. Reduction of the sperm motility, sperm viability and the mean fluorescence intensity of CatSper1 to CatSper4 in the epididymal sperm of asthenozoospermic (AZS) model rats. Related to Fig. 3. (A-B) Representative images and a summary for the percentage of sperm with abnormal morphology (n = 188-222 sperm from 3 rats per group). Arrows indicate the sperm with abnormal morphology. (C) Sperm concentration, (D) Sperm viability, (E-J) Sperm motility including straight-line velocity (VSL) (E), curve-line velocity (VCL) (F), average path velocity (VAP) (G), amplitude of lateral head displacement (ALH) (H), linearity (LIN) (I), and straightness (STR) (J). n = 10 rats per group. (K-O) Immunofluorescence staining of CatSper (from CatSper1 to CatSper4) in the epididymal sperm of AZS rats. Shown are representative images for the immunofluorescence staining of CatSper1-CatSper4 (green) with the nucleus marker DAPI (blue) (K), and a summary for the mean fluorescence intensity of

CatSper1-CatSper4 immunostaining (L-O) (n = 80-94 sperm from 3 rats per group). Scale bar = 75 μm. All data are presented as mean ± SEM. $^{*}P < 0.05$, $^{**}P < 0.01$, $^{***}P < 0.001$. One-way ANOVA with Sidaks *post-hoc* test for (B)-(J); Unpaired *t* test for (L)-(O).

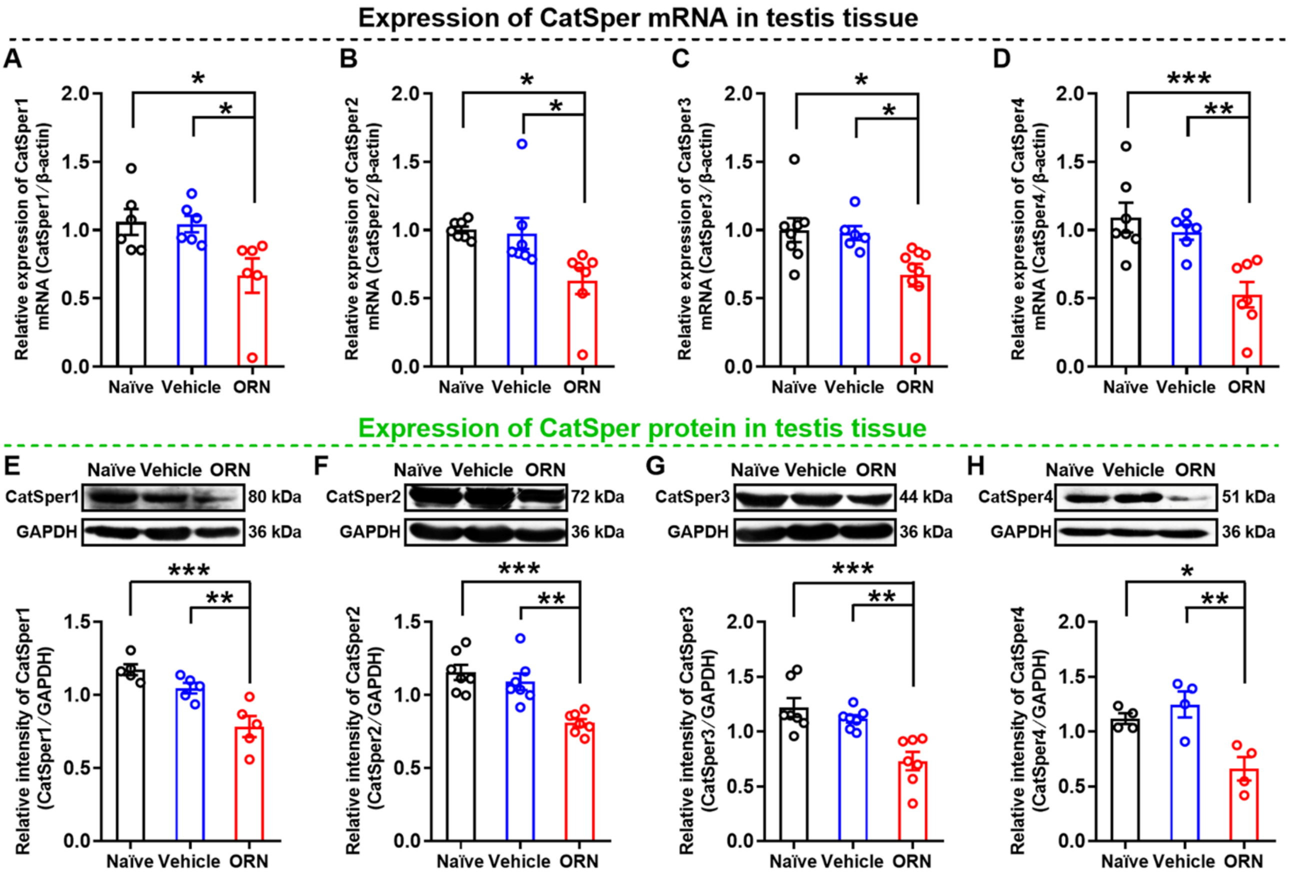

Figure S3. Reduction of CatSper (from CatSper1 to CatSper4) mRNA and protein abundance in the testis tissues of asthenozoospermic (AZS) model rats. Related to Fig. 3. (A-D) Quantitative real-time PCR assay of CatSper1 (A), CatSper2 (B), CatSper3 (C), and CatSper4 (D) mRNA abundance in the testis tissues of naïve rats, vehicle-treated rats, and ornidazole (ORN)-induced AZS rats (n = 6-9 rats per group). (E-H) Western blot of CatSper1 (E), CatSper2 (F), CatSper3 (G), and CatSper4 (H) protein abundance in the testis tissues of naïve rats, vehicle-treated rats, and ornidazole (ORN)-induced AZS rats (n = 4-7 rats per group). One-way ANOVA followed by Sidaks multiple comparisons test. All data are presented as mean ± SEM. $^{*}P < 0.05$, $^{**}P < 0.01$, $^{***}P < 0.001$. One-way ANOVA with Sidaks *post-hoc* test for (A)-(H).

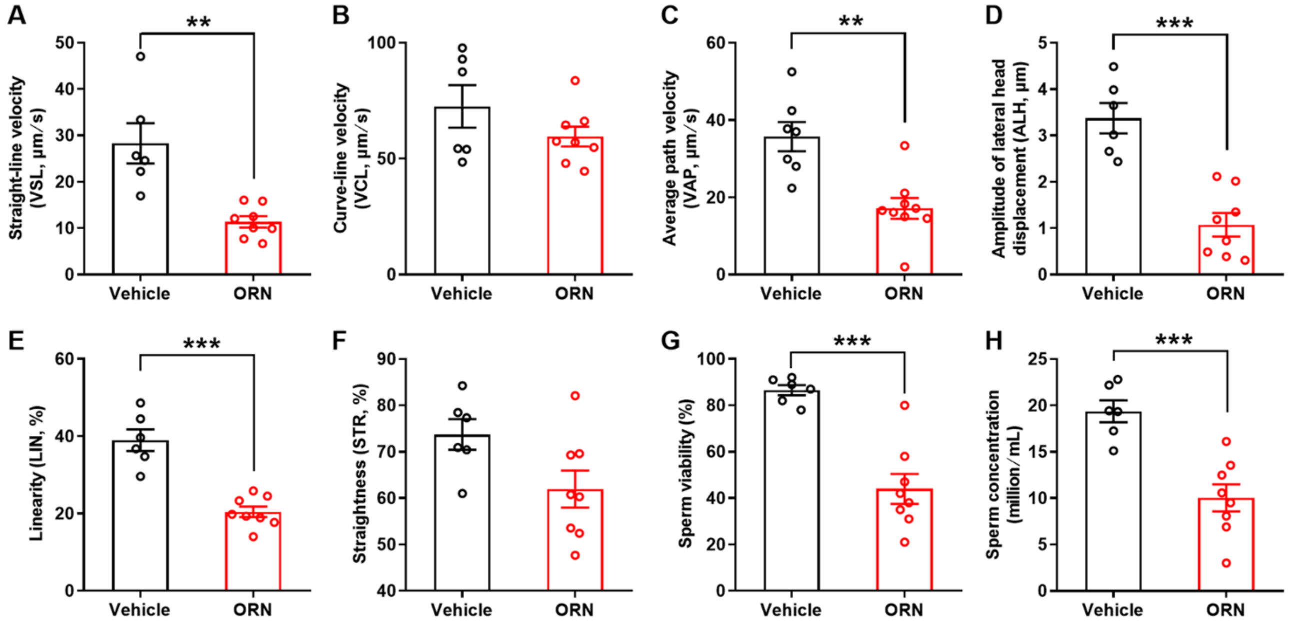

Figure S4. Validation of the decreased sperm motility of the ORN-induced asthenozoospermic (AZS) male rats that were used to mate with the normal female rats in the *in vivo* fertility assay experiments. Related to Fig. 3. (A-F) Sperm motility including straight-line velocity (VSL) (A), curve-line velocity (VCL) (B), average path velocity (VAP) (C), amplitude of lateral head displacement (ALH) (D), linearity (LIN) (E), and straightness (STR) (F). (G) Sperm viability. (H) Sperm concentration. n = 6-8 rats per group. All data are presented as mean ± SEM. $^{**}P < 0.01$, $^{***}P < 0.001$. Unpaired *t* test for (A)-(H).

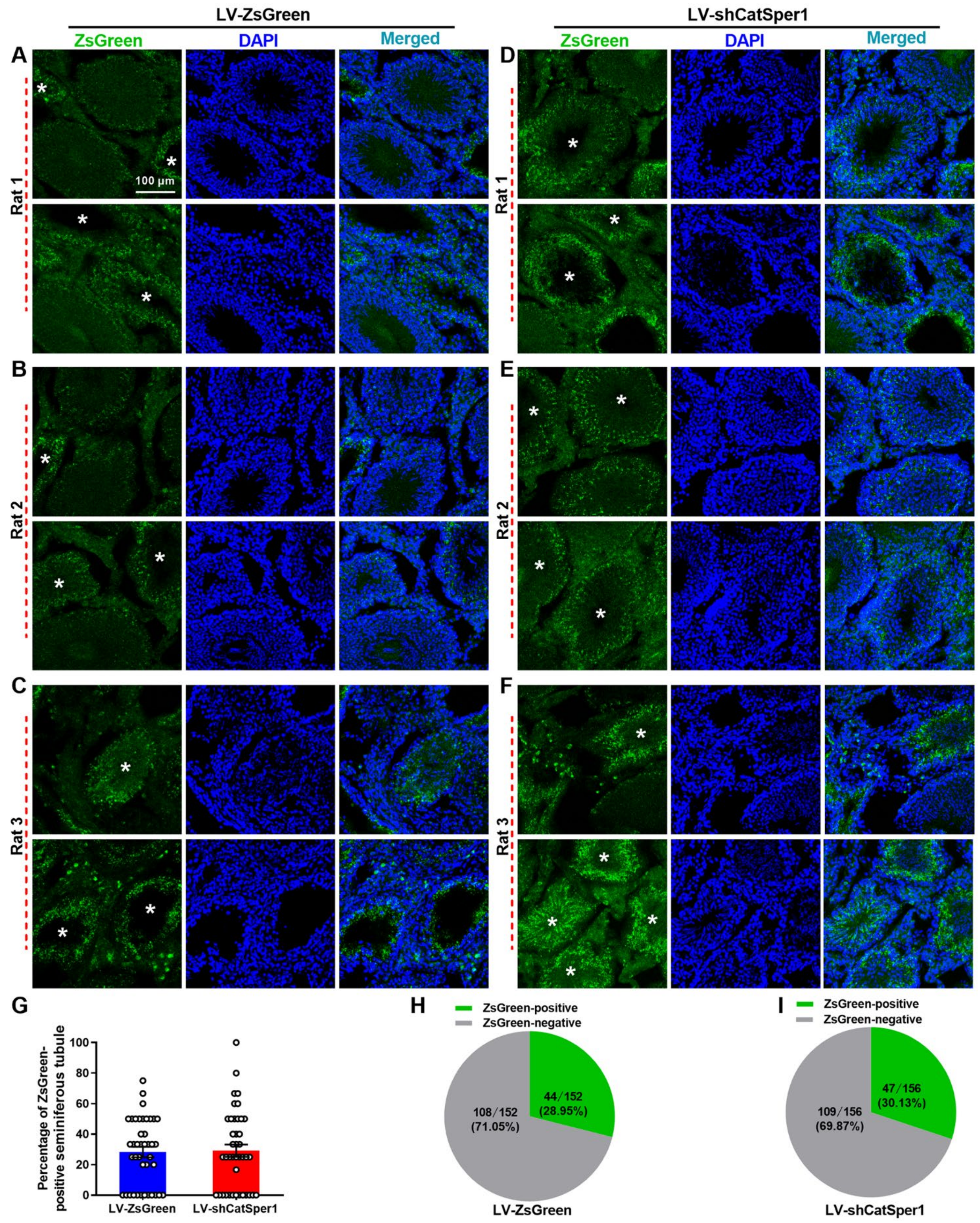

Figure S5. Validation of the lentivirus infection efficiency by immunofluorescence staining. Related to Fig. 4. Shown are representative images for the immunofluorescence staining with ZsGreen (green) and the nuclear marker DAPI (blue) in the testis tissues of rats on day 14 after in situ injection of lentivirus-expressing ZsGreen (LV-ZsGreen) (A-C) and lentivirus-expressing CatSper1 shRNA coupled

to a ZsGreen tag (LV-shCatSper1) (D-F), respectively. Asterisk indicates the seminiferous tubule with LV-ZsGreen or LV-shCatSper1 infection. Scale bar = 100 μm. (G-I) A summary for the percentage of ZsGreen positive seminiferous tubule in all visual seminiferous tubule of LV-ZsGreen or LV-shCatSper1 infected-rats (G), and the percentage of ZsGreen-positive or ZsGreen-negative seminiferous tubule in LV-ZsGreen (H) and LV-shCatSper1 (I) infected-rats, respectively. n = 152-156 seminiferous tubule from 3 rats.

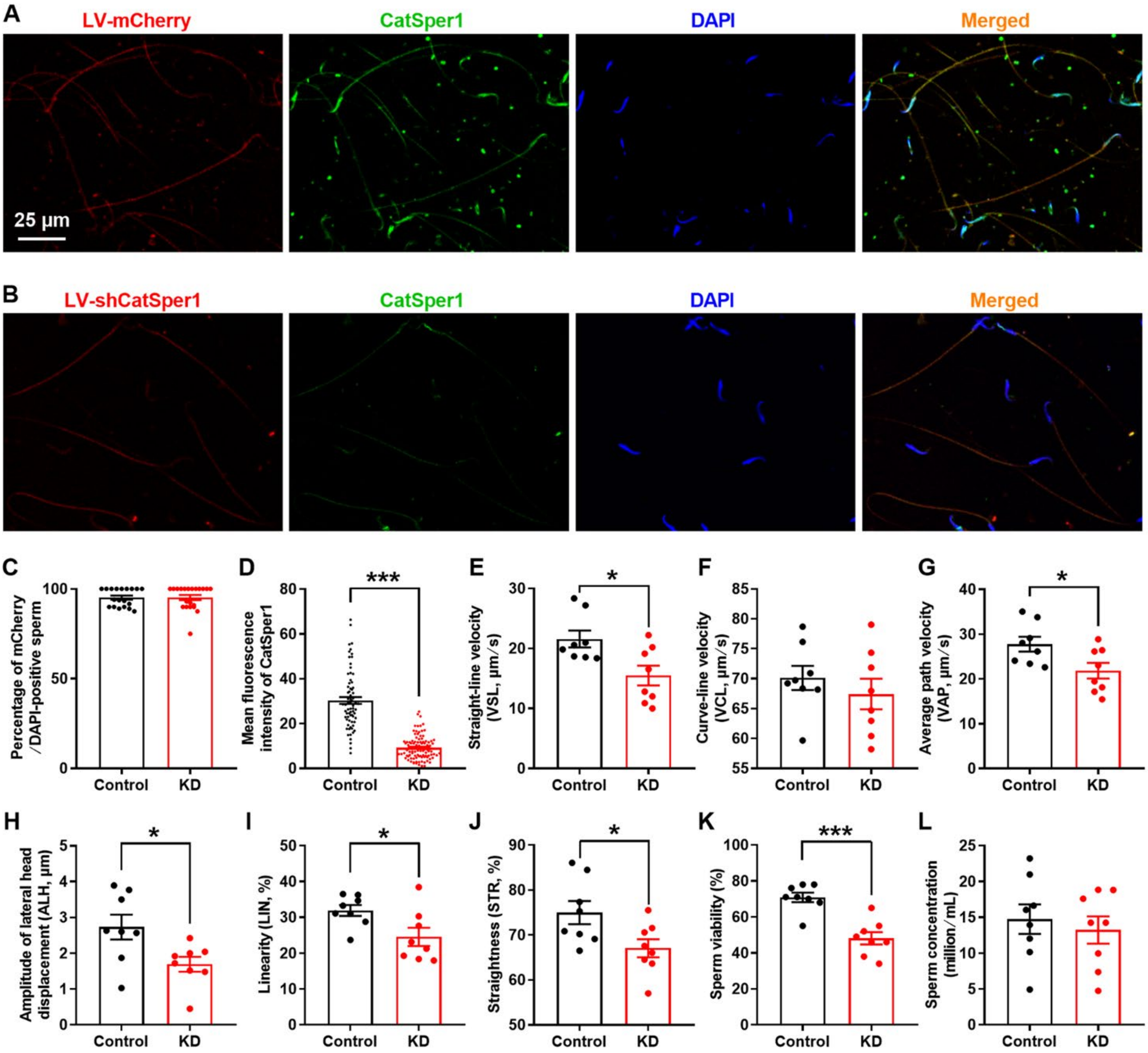

Figure S6. Reduction of CatSper1 immunofluorescence staining and impairment of sperm motility in the epididymal sperm of CatSper1 knockdown (KD) rats. Related to Fig. 4. (A-D) Immunofluorescence staining. Shown are representative images for the immunofluorescence staining of CatSper1 (green) with mCherry (red) and the nuclear marker DAPI (blue) (A, B), and a summary for the percentage of mCherry/DAPI-positive sperm (C) and the mean fluorescence intensity of CatSper1 immunostaining (D) (n = 72-102 sperm from 3 rats per group). (E-J) Sperm motility including straight-line velocity (VSL) (E), curve-line velocity (VCL) (F), average path velocity (VAP) (G), amplitude of lateral head displacement (ALH) (H), linearity (LIN) (I), and straightness (STR) (J). (K) Sperm viability. (L) Sperm concentration. n = 8 rats per group. All data are presented as mean ± SEM. $^{*}P < 0.05$, $^{***}P < 0.001$. Unpaired *t* test for (C)-(L).

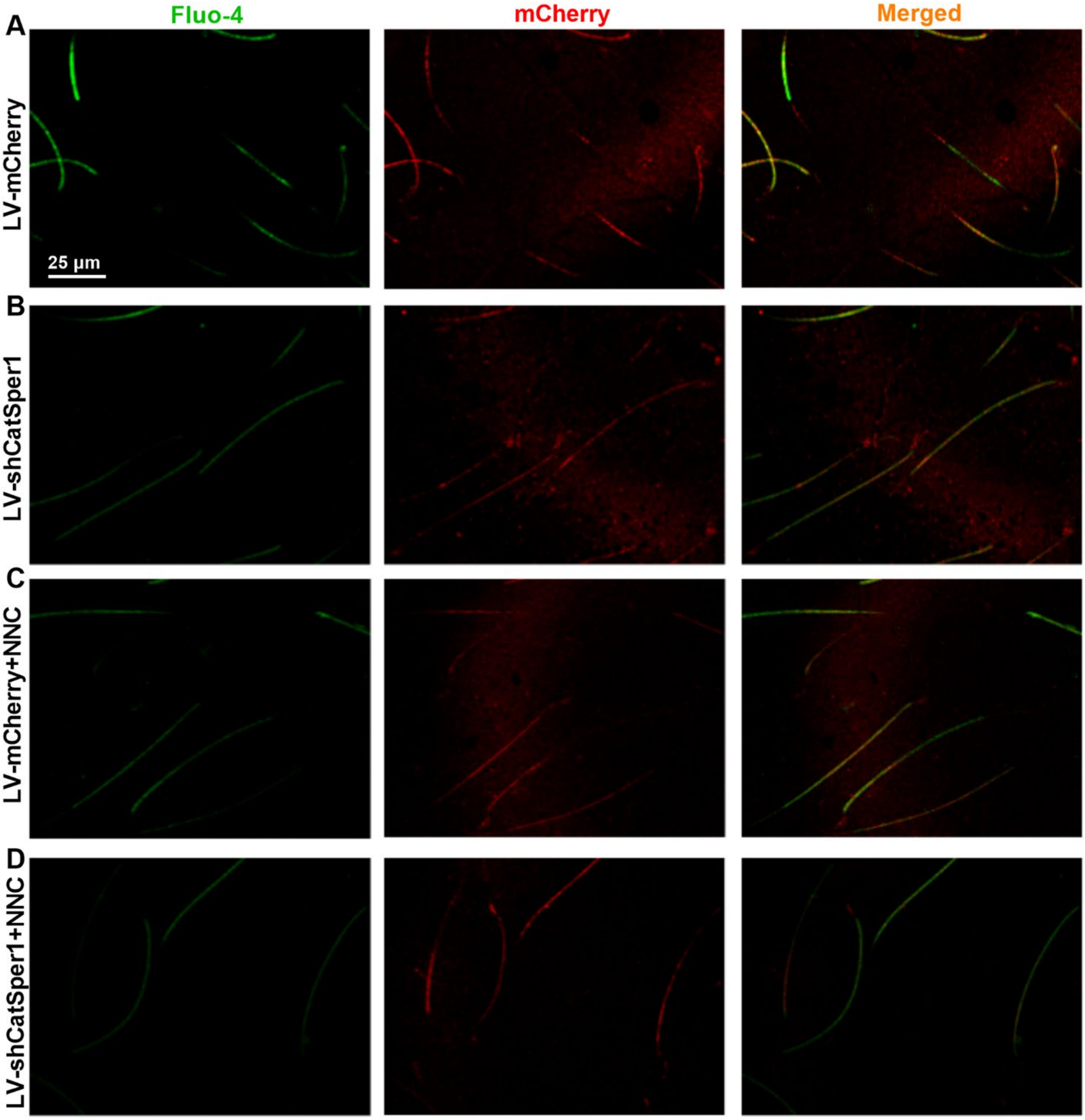

Figure S7. Immunofluorescence staining with fluo-4 and mCherry in the epididymal sperm of rats received LV-shCatSper1 or LV-mCherry injection. Related to Fig. 4. Shown are representative images for the immunofluorescence staining with fluo-4 (green) and mCherry (red) in the epididymal sperm of rats in different groups as indicated (n = 4-5 rats per group). Scale bar = 25 μm.

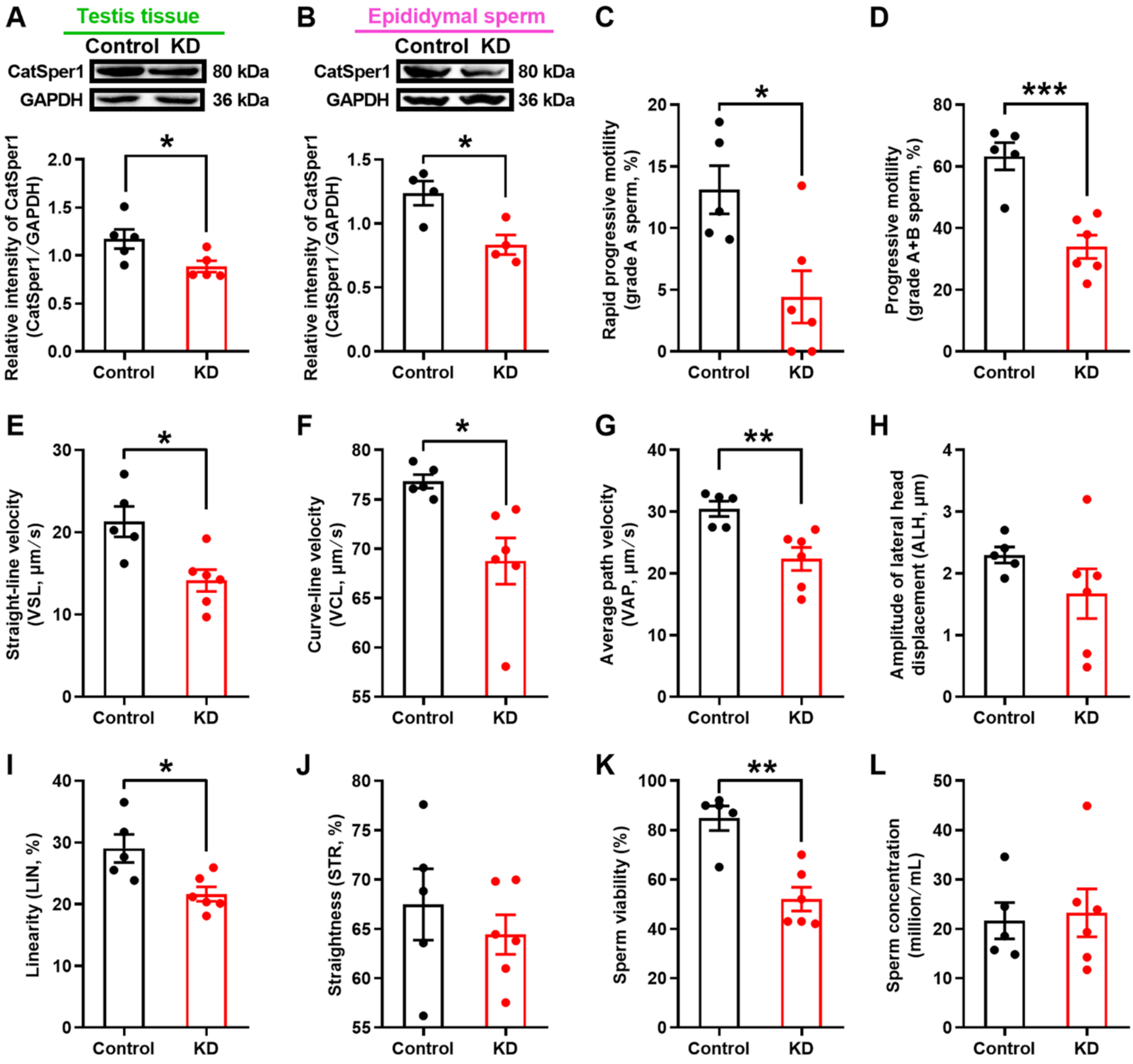

Figure S8. Validation of the decreased sperm motility of the CatSper1 knockdown (KD) rats that were used to mate with the normal female rats in the *in vivo* fertility assay experiments. Related to Fig. 4. (A-B) CatSper1 protein abundance in the testis tissues (A) and epididymal sperm (B) (n = 4-5 rats per group). (C-J) Sperm motility including rapid progressive motility (grade A sperm) (C), progressive motility (grade A+B sperm) (D), straight-line velocity (VSL) (E), curve-line velocity (VCL) (F), average path velocity (VAP) (G), amplitude of lateral head displacement (ALH) (H), linearity (LIN) (I), and straightness (STR) (J). (K) Sperm viability. (L) Sperm concentration. n = 5-6 rats per group. All data are presented as mean ± SEM. $^{*}P < 0.05$, $^{**}P < 0.01$, $^{***}P < 0.001$. Unpaired *t* test for (A)-(L).

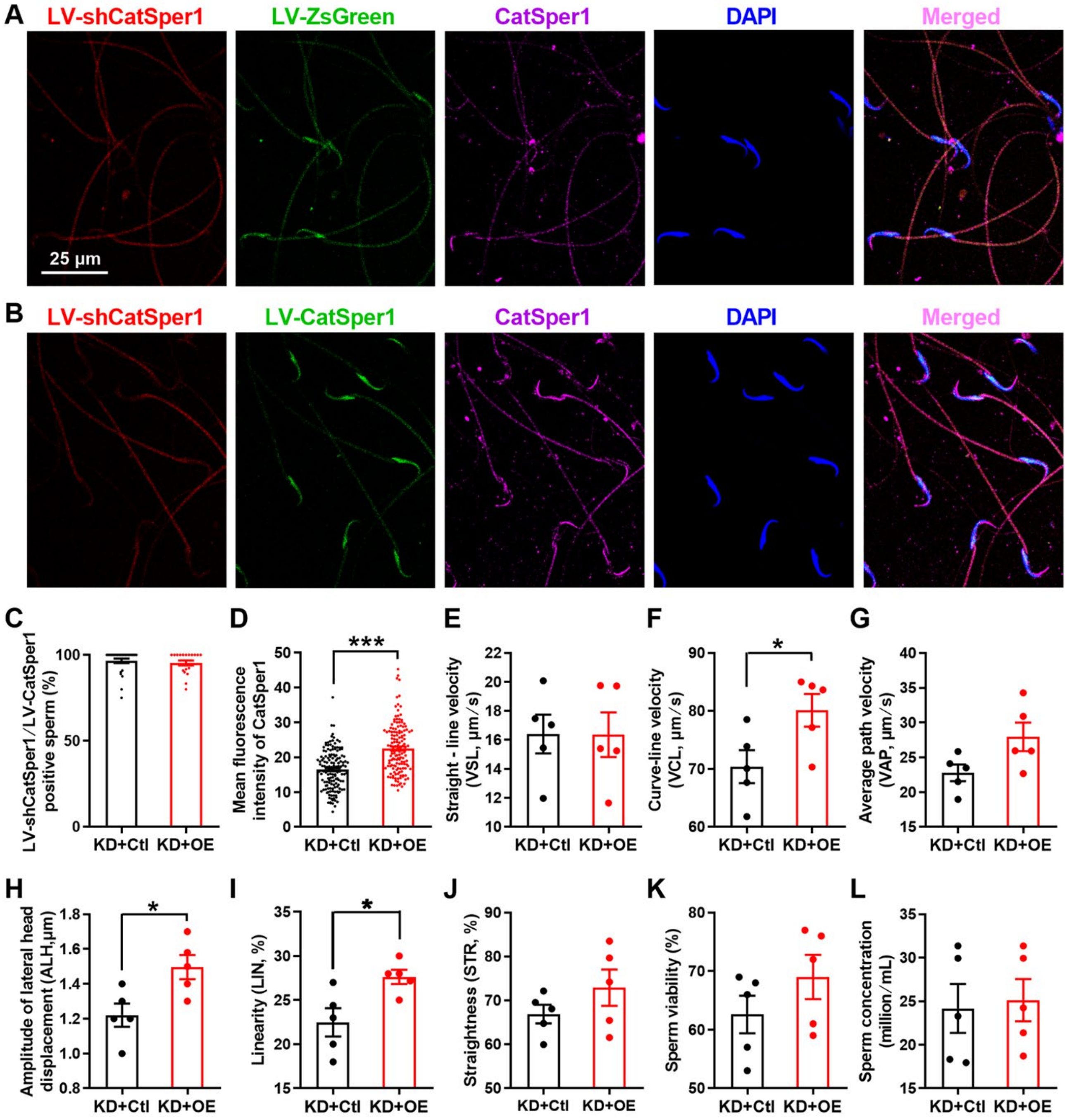

Figure S9. CatSper1 overexpression (OE) rescues the impaired sperm motility of CatSper1 knockdown (KD) rats. Related to Fig. 5. Overexpression of CatSper1 was performed by in situ injection of lentivirus-expressing CatSper1 coupled to ZsGreen tag (LV-CatSper1) into the testis tissues of rats on day 11 after the lentivirus-expressing CatSper1 shRNA coupled to mCherry tag (LV-shCatSper1) injection. (A-D) Validation of CatSper1 overexpression by immunofluorescence staining of CatSper1 with ZsGreen, mCherry, and the nucleus marker DAPI. Shown are representative images for the immunofluorescence staining of CatSper1 (purple) with mCherry (red), ZsGreen (green), and the nuclear marker DAPI (blue) (A-B), and a summary for the percentage of LV-shCatSper1/LV-CatSper1-positive sperm (C) and the mean fluorescence intensity of CatSper1 immunostaining (D) (n = 147-149 sperm from 3 to 4 rats per group). Scale bar = 25 μm. (E-J) Sperm motility including straight-line velocity (VSL) (E), curve-line

velocity (VCL) (F), average path velocity (VAP) (G), amplitude of lateral head displacement (ALH) (H), linearity (LIN) (I), and straightness (STR) (J). (K) Sperm viability. (L) Sperm concentration. n = 5 rats per group. All data are presented as mean ± SEM. $^{*}P < 0.05$, $^{***}P < 0.001$. Unpaired *t* test for (C)-(L).

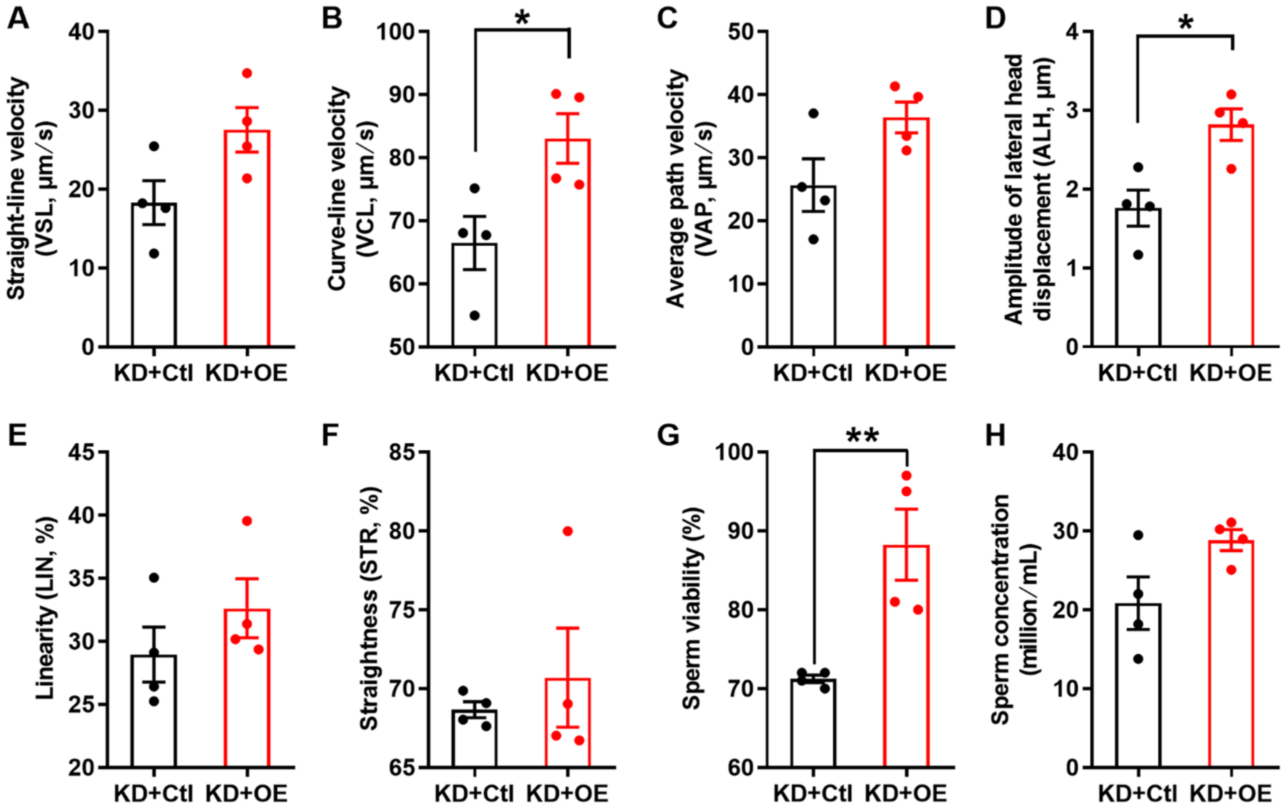

Figure S10. Validation of the improved sperm motility of the CatSper1 knockdown (KD) rats with CatSper1 overexpression (OE) in the *in vivo* fertility assay experiments. Related to Fig. 5. (A-F) Sperm motility including straight-line velocity (VSL) (A), curve-line velocity (VCL) (B), average path velocity (VAP) (C), amplitude of lateral head displacement (ALH) (D), linearity (LIN) (E), and Straightness (STR) (F). (G) Sperm viability. (H) Sperm concentration. n = 4 rats per group. All data are presented as mean ± SEM. $^{*}P < 0.05$, $^{**}P < 0.01$. Unpaired *t* test for (A)-(H).

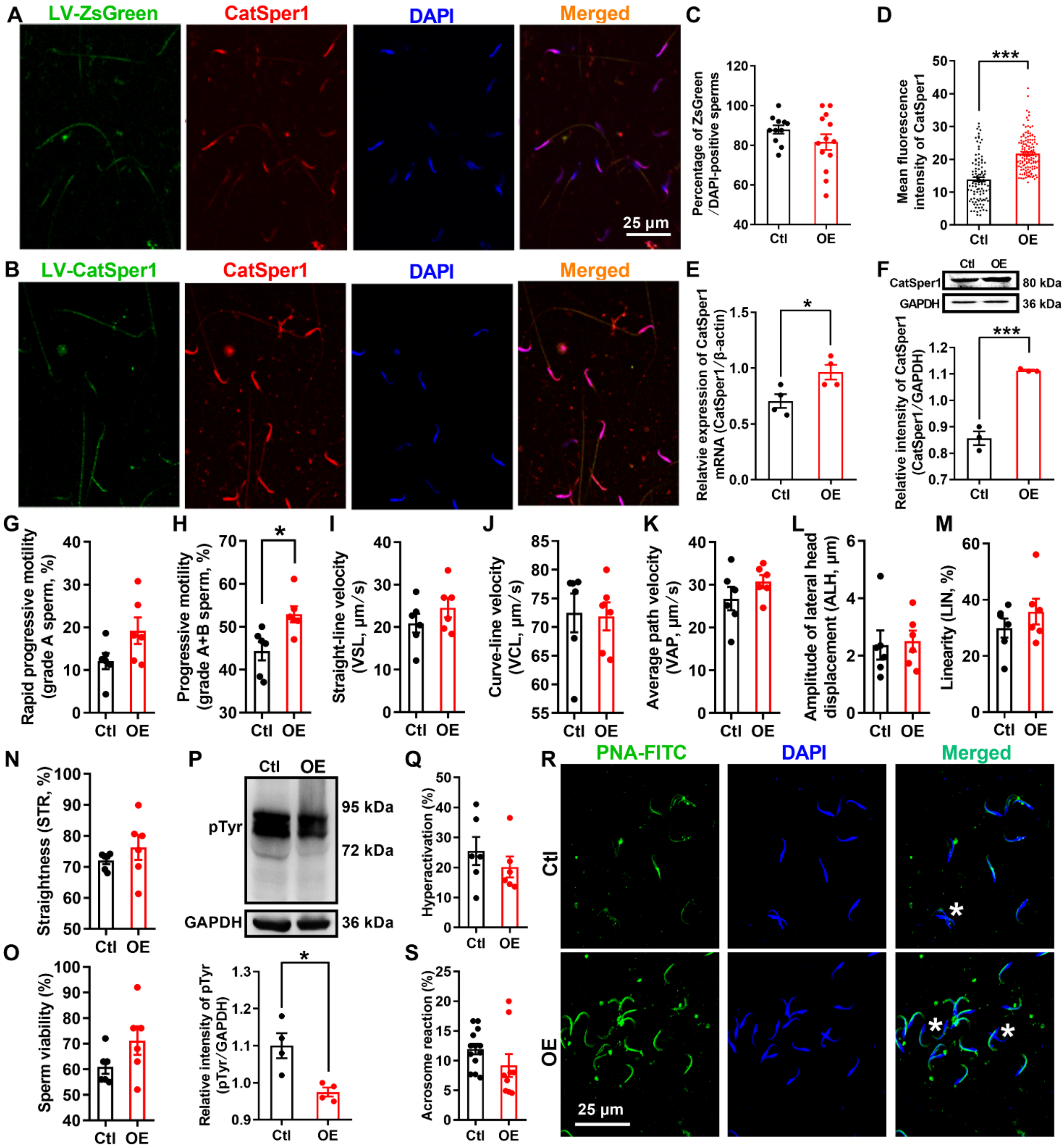

Figure S11. CatSper1 overexpression cannot rescue the impaired sperm motility and functional characteristics of asthenozoospermic (AZS) model rats. Related to Fig. 5. (A-F) Validation of CatSper1 overexpression (OE). (A-D) Immunofluorescence staining. Shown are representative images for the immunofluorescence staining of CatSper1 (red) with ZsGreen (green) and the nuclear marker DAPI (blue) (A-B), and a summary for the percentage of ZsGreen/DAPI-positive sperm (C) and the mean fluorescence intensity of CatSper1 immunostaining (D) (n = 103-132 sperm from 3 to 4 rats per group). Scale bar = 25 μm. (E-F) CatSper1 mRNA (E) and protein (F) abundance in the epididymal sperm of CatSper1 overexpression (OE) and control (Ctl) rats, respectively (n = 3-4 rats per group). (G-O) Sperm motility including grade A sperm (G), grade A+B sperm (H), straight-line velocity (VSL) (I), curve-line

velocity (VCL) (J), average path velocity (VAP) (K), amplitude of lateral head displacement (ALH) (L), linearity (LIN) (M), straightness (STR) (N), and sperm viability (O) in AZS rats with CatSper1 overexpression. n = 6 rats per group. (P-S) Protein tyrosine phosphorylation (pTyr) (P), hyperactivation (Q), and acrosome reaction (AR) (R-S) in the epididymal sperm of AZS rats with CatSper1 overexpression. Representative images of sperm acrosome reaction (R) and a summary plot for the percentage of sperm acrosome reaction (S) are shown (n = 4-6 rats per group). Asterisk indicates the sperm that has acrosome reaction (acrosome disappeared). Scale bar = 25 μm. All data are presented as mean ± SEM. $^{*}P < 0.05$, $^{***}P < 0.001$. Unpaired *t* test for (C)-(Q), and (S).

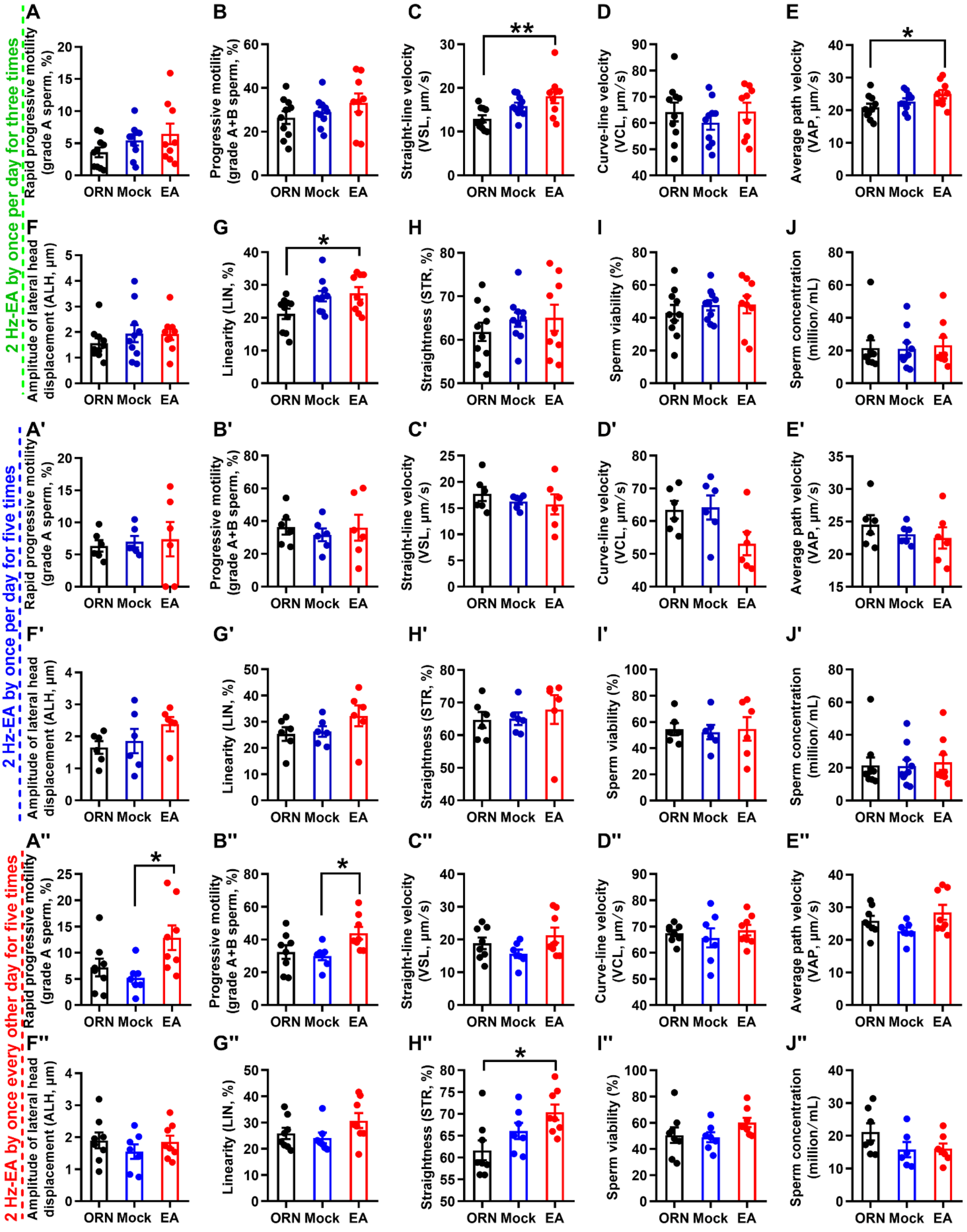

Figure S12. Determination of the protocols for 2 Hz-EA treatment on the sperm motility of asthenozoospermic (AZS) model rats. Related to Fig. 6. (A-J) 2 Hz-EA treatment by once per day for three times. (A′-J′) 2 Hz-EA treatment by once per day for five times. (A″-J″) 2 Hz-EA treatment by once every other day for three times. (A, A′, and A″) rapid progressive motility (grade A) sperm. (B, B′, and

B″) progressive motility (grade A+B) sperm. (C, C′, and C″) Straight-line velocity (VSL). (D, D′, and D″) Curve-line velocity (VCL). (E, E′, and E″) Average path velocity (VAP). (F, F′, and F″) Amplitude of lateral head displacement (ALH). (G, G′, and G″) linearity (LIN). (H, H′, and H″) Straightness (STR). (I, I′, and I″) Sperm viability. (J, J′, and J″) Sperm concentration. n = 6-10 rats per group. All data are presented as mean ± SEM. $^{*}P < 0.05$, $^{**}P < 0.01$. One-way ANOVA with Sidaks *post-hoc* test for (A)-(J″).

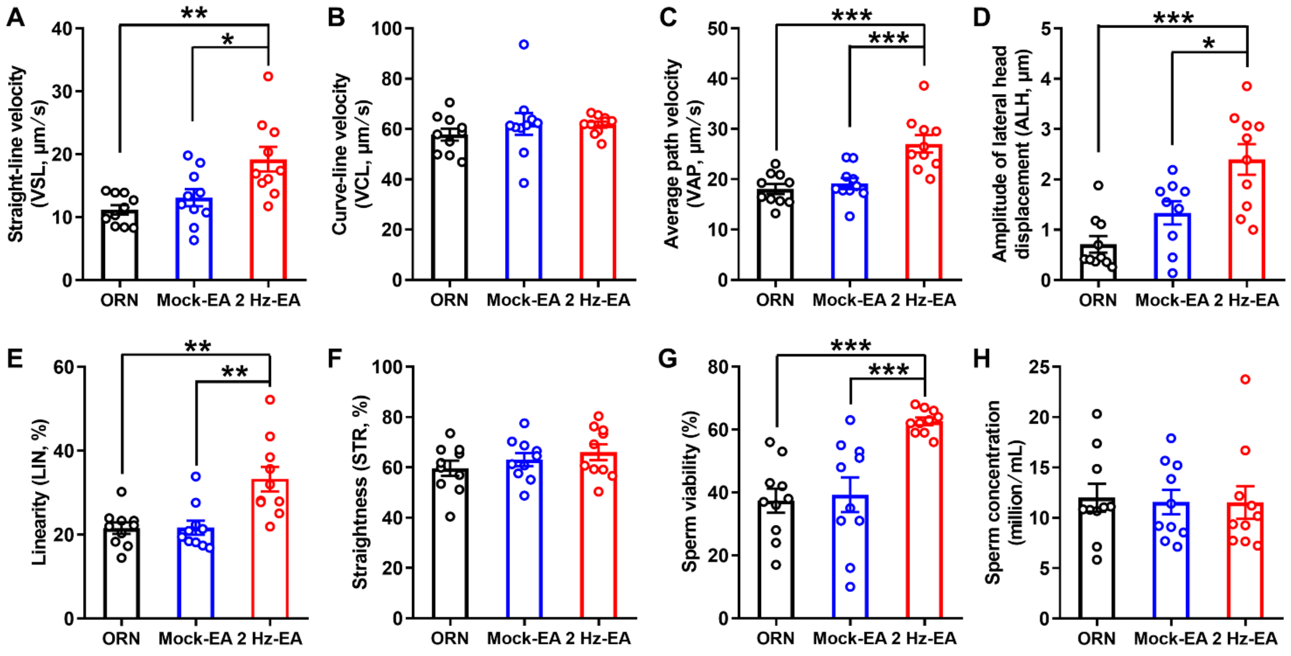

Figure S13. Improved sperm motility and viability of asthenozoospermic (AZS) rats with 2 Hz-EA treatment, by the protocol of once every other day for five times. Related to Fig. 6. (A-F) Sperm motility including straight-line velocity (VSL) (A), curve-line velocity (VCL) (B), average path velocity (VAP) (C), amplitude of lateral head displacement (ALH) (D), linearity (LIN) (E), and straightness (STR) (F). (G) Sperm viability. (H) Sperm concentration. n = 10 rats per group. All data are presented as mean ± SEM. $^{*}P < 0.05$, $^{**}P < 0.01$, $^{***}P < 0.001$. One-way ANOVA with Sidaks *post-hoc* test for (A)-(H).

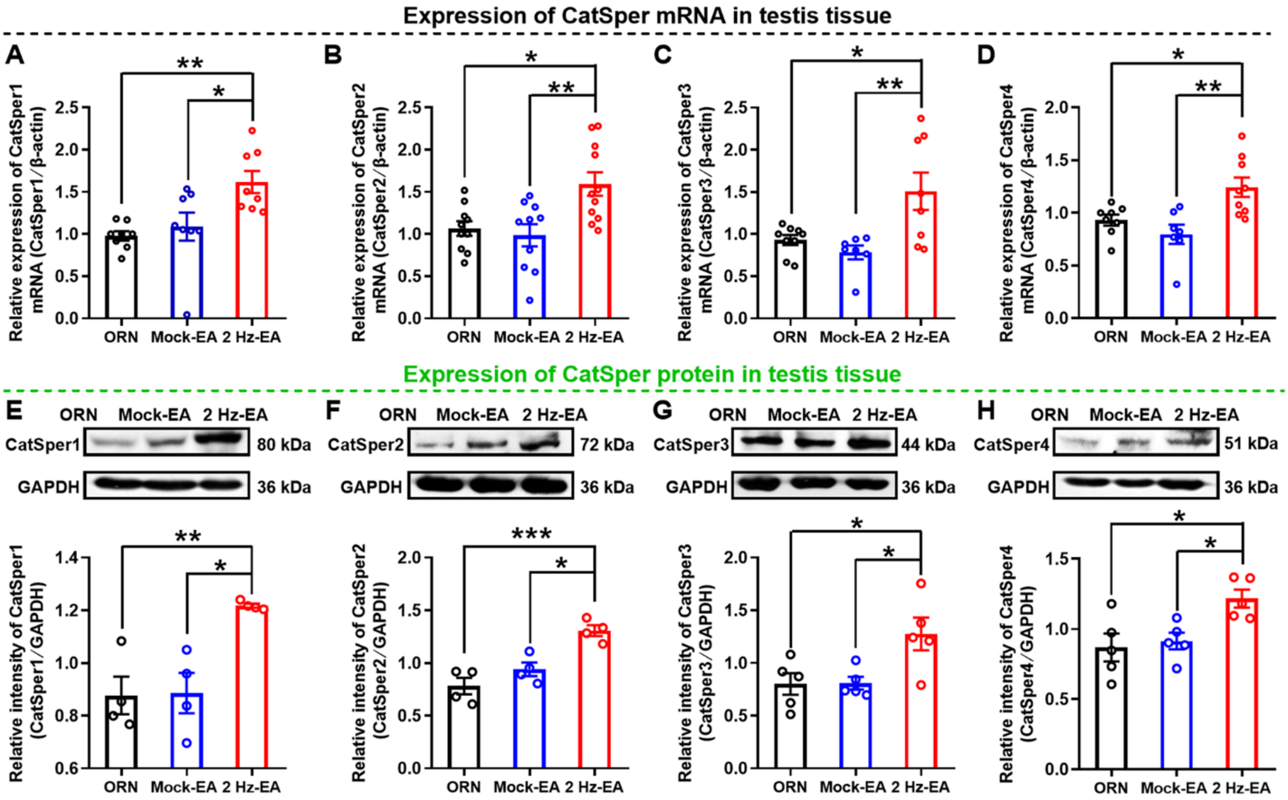

Figure S14. Enhancement of CatSper mRNA and protein (from CatSper1 to CatSper4) abundance in the testis tissues of asthenozoospermic (AZS) rats with 2 Hz-EA treatment, by the protocol of once every other day for five times. Related to Fig. 6. (A-D) CatSper (CatSper1-CatSper4) mRNA levels. (E-H) CatSper (CatSper1-CatSper4) protein abundance. n = 4-11 rats per group. All data are presented as mean ± SEM. $^{*}P < 0.05$, $^{**}P < 0.01$, $^{***}P < 0.001$. One-way ANOVA with Sidaks *post-hoc* test for (A)-(H).

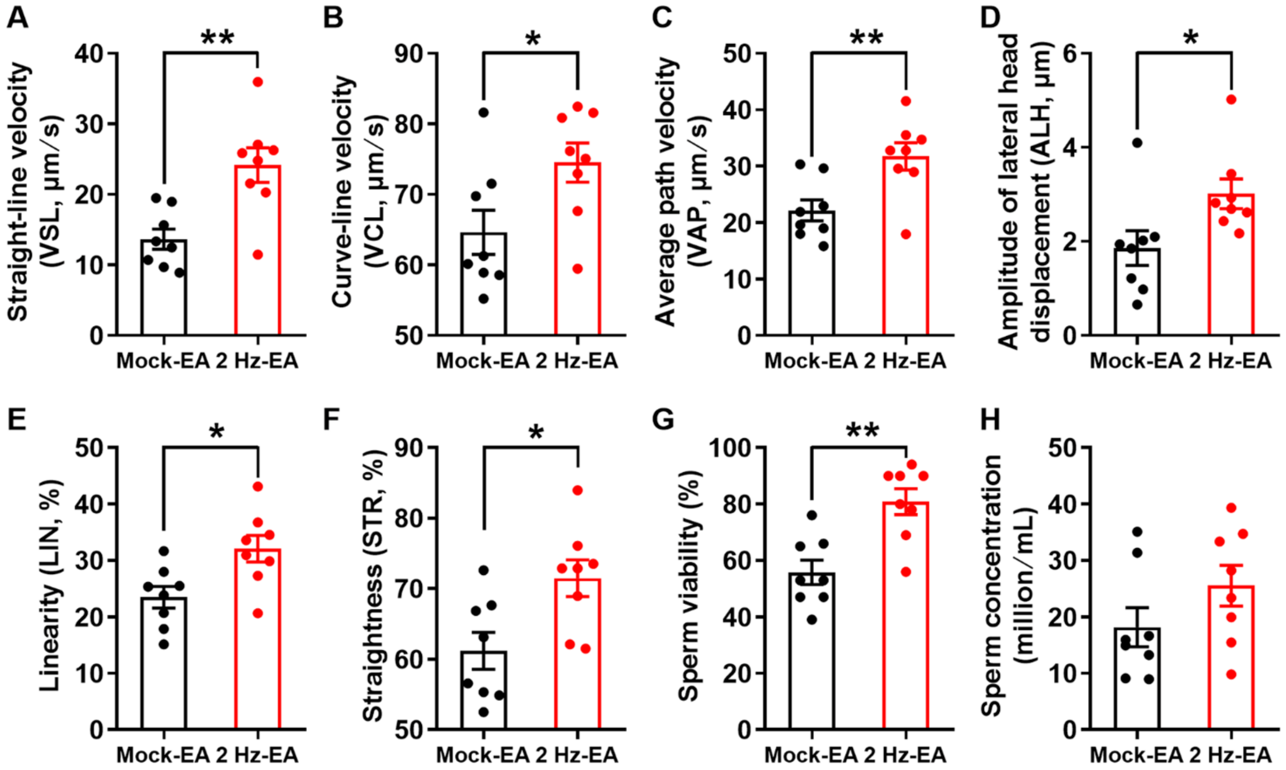

Figure S15. Validation of improved sperm motility of 2 Hz EA-treated asthenozoospermic (AZS) male rats that were used to mate with the normal female rats in the *in vivo* fertility assay experiments. Related to Fig. 6. (A-F) Sperm motility including straight-line velocity (VSL) (A), curve-line velocity (VCL) (B), average path velocity (VAP) (C), amplitude of lateral head displacement (ALH) (D), linearity (LIN) (E), and straightness (STR) (F). (G) Sperm viability. (H) Sperm concentration. n = 8 rats per group. All data are presented as mean ± SEM. $^{*}P < 0.05$, $^{**}P < 0.01$. Unpaired *t* test for (A)-(H).

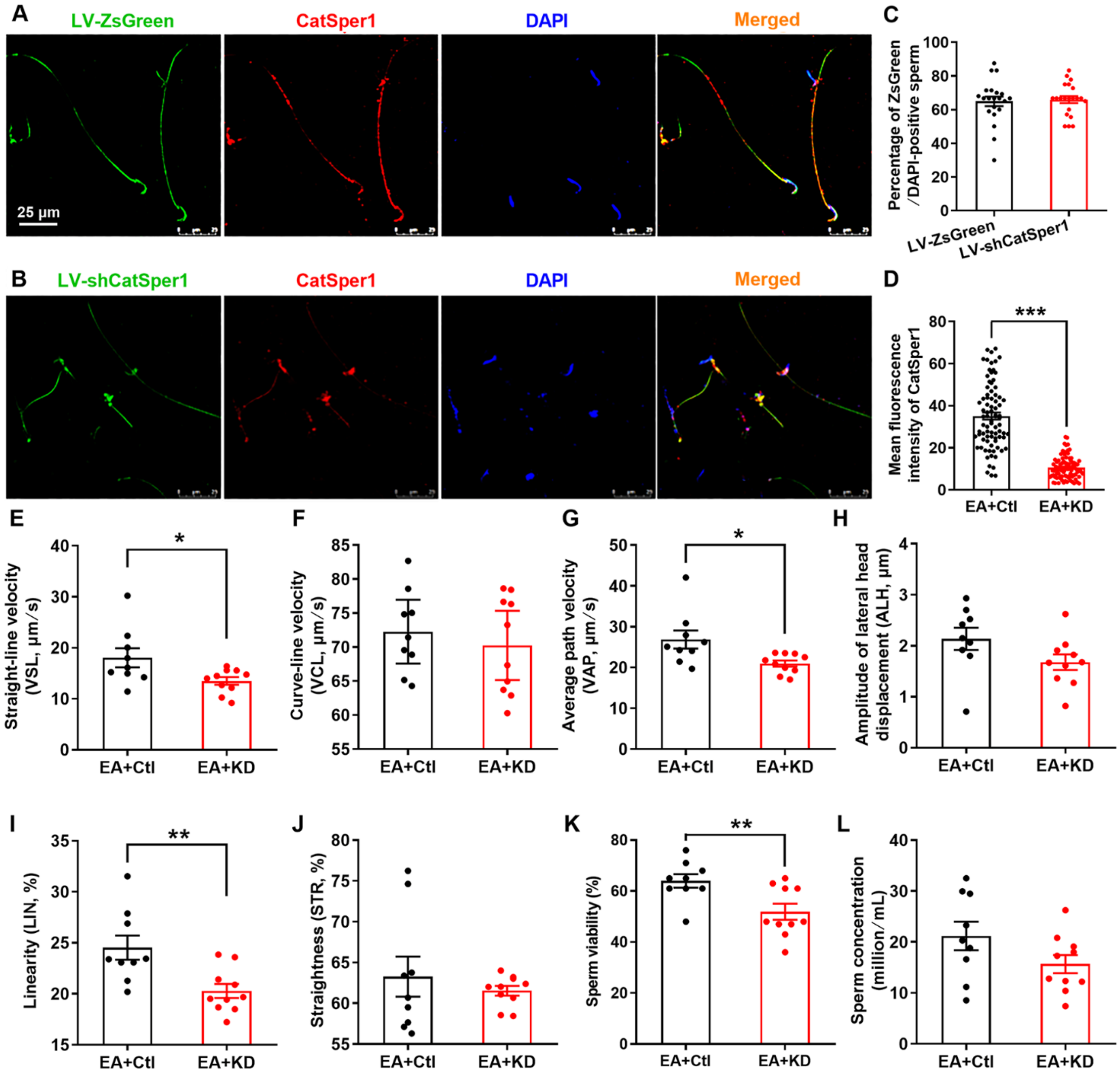

Figure S16. CatSper1 knockdown abolishes the 2 Hz EA-induced improvement of sperm motility to asthenozoospermic (AZS) rats. Related to Fig. 7. (A-D) Validation of CatSper1 knockdown in the epididymal sperm of rats that received in situ injection of LV-shCatSper1. Shown are representative images for the immunofluorescence staining of CatSper1 (red) with ZsGreen (green) and the nuclear marker DAPI (blue) (A-B), and a summary for the percentage of ZsGreen/DAPI-positive sperm (C) and the mean fluorescence intensity of CatSper1 immunostaining (D) (n = 83-86 sperm from 3 rats per group). (E-J) Sperm motility including straight-line velocity (VSL) (E), curve-line velocity (VCL) (F), average path velocity (VAP) (G), amplitude of lateral head displacement (ALH) (H), linearity (LIN) (I), and straightness (STR) (J). (K) Sperm viability. (L) Sperm concentration. n = 9-10 rats per group. All data are presented as mean ± SEM. *P < 0.05, **P < 0.01, ***P<0.001. Unpaired *t* test for (C)-(L).

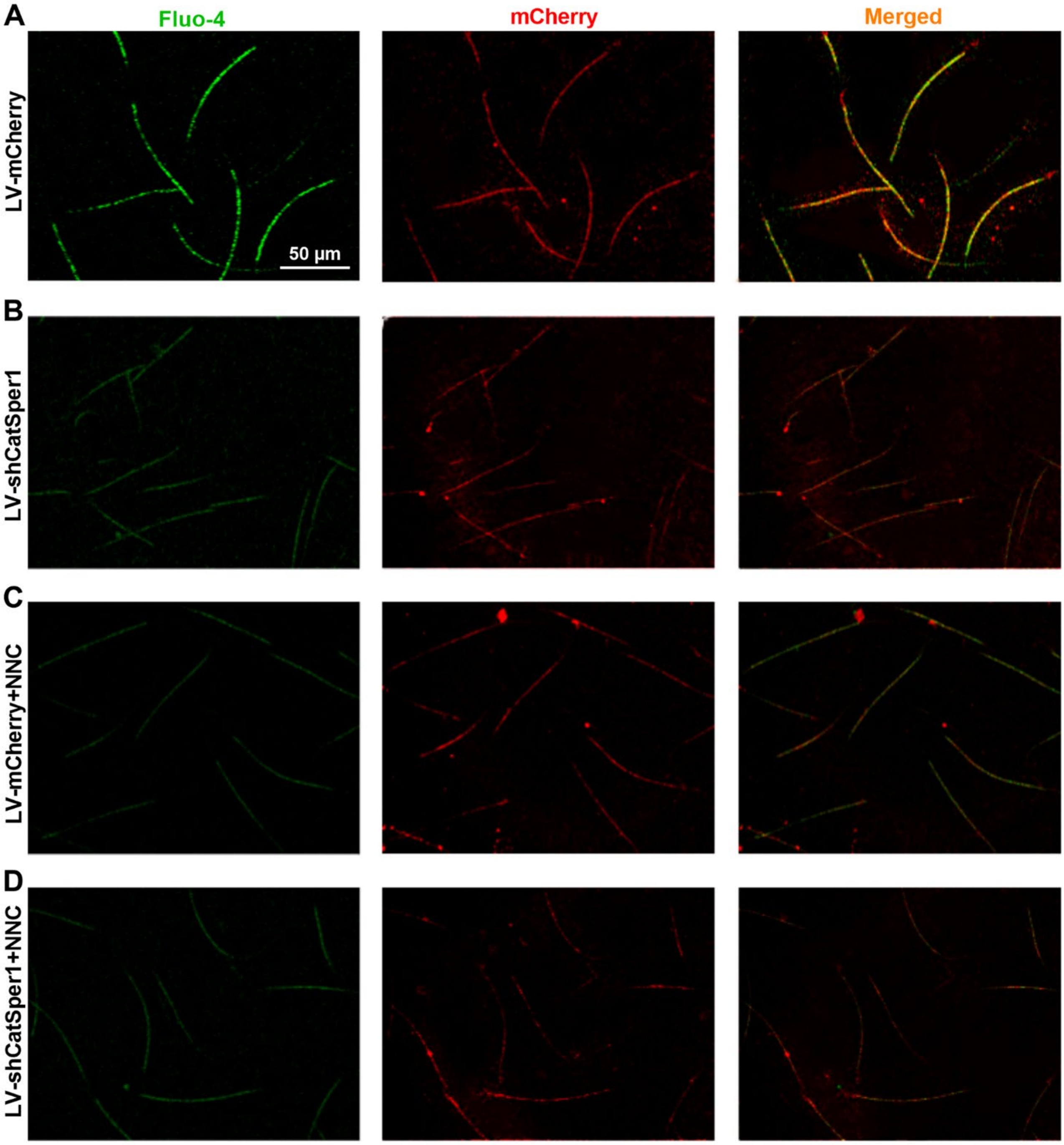

Figure S17. Immunofluorescence staining with fluo-4 and mCherry in the epididymal sperm of 2 Hz EA-treated asthenozoospermic (AZS) rats infected with LV-shCatSper1 or LV-mCherry. Related to Fig. 7. Shown are representative images for the immunofluorescence staining with fluo-4 (green) and mCherry (red) in the epididymal sperm of rats in different groups as indicated. Scale bar = 50 µm.

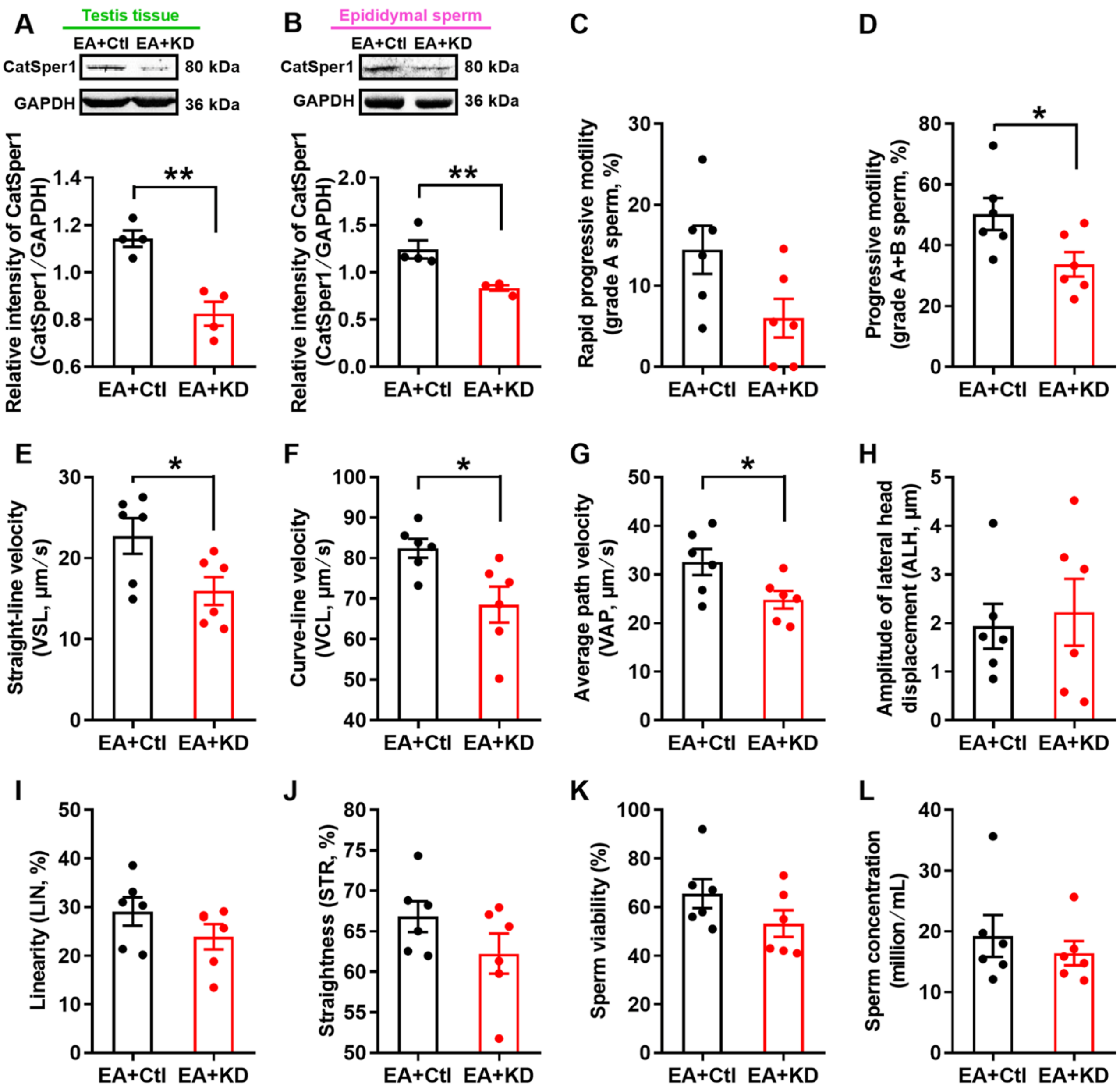

Figure S18. Validation of impaired sperm motility of 2 Hz EA-treated asthenozoospermic male rats by CatSper1 knockdown (KD) in the *in vivo* fertility assay experiments. Related to Fig. 7. (A-B) CatSper1 protein abundance in the testis tissues (A) and epididymal sperm (B). n = 4 rats per group. (C-J) Sperm motility including grade A sperm (C), grade A+B sperm (D), straight-line velocity (VSL) (E), curve-line velocity (VCL) (F), average path velocity (VAP) (G), amplitude of lateral head displacement (ALH) (H), linearity (LIN) (I), straightness (STR) (J). (K) Sperm viability. (L) Sperm concentration. n = 6 rats per group. All data are presented as mean ± SEM. $^{*}P < 0.05$, $^{**}P < 0.01$. Unpaired *t* test for (A)-(L).

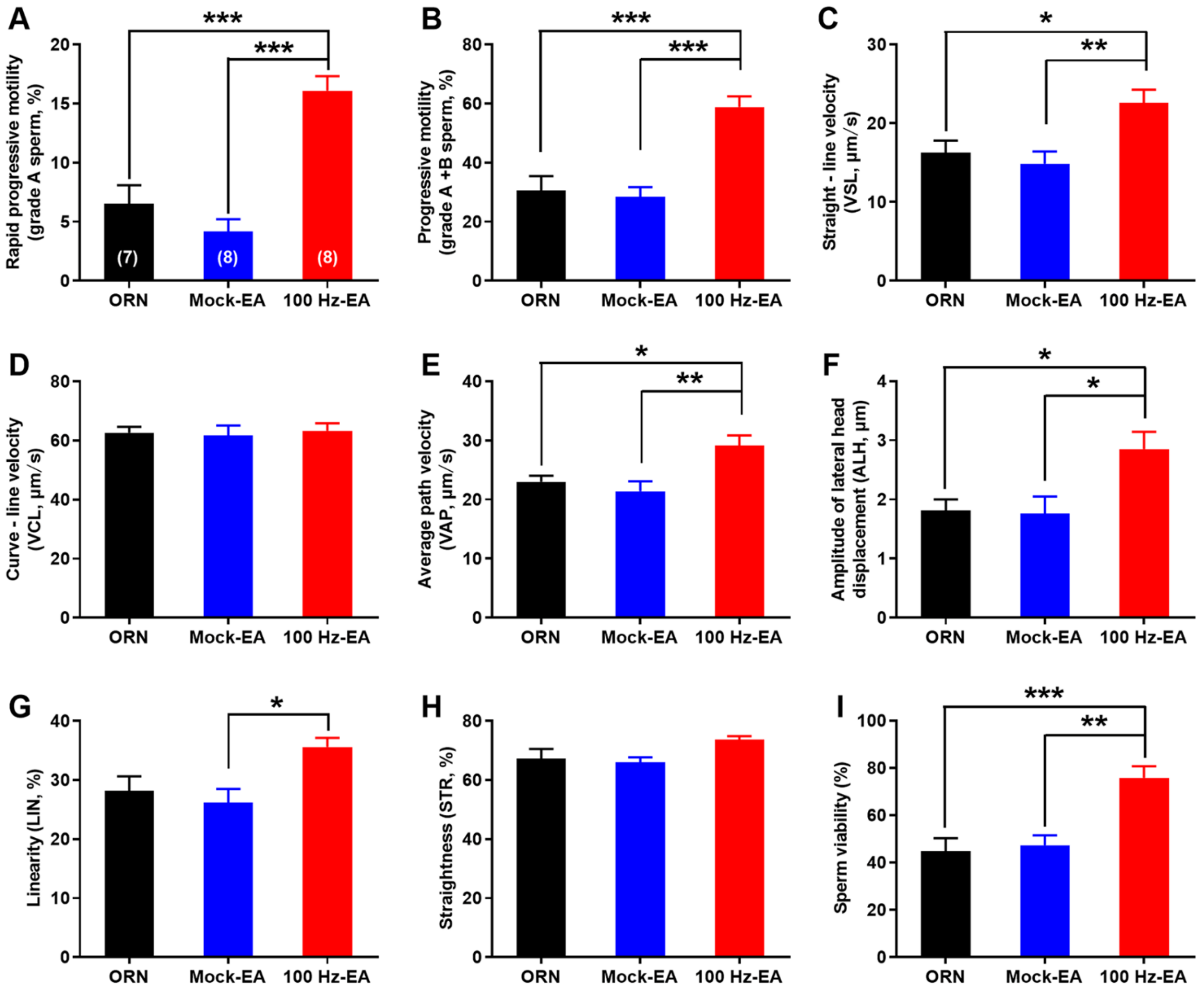

Figure S19. Effects of 100 Hz-EA treatment (by the protocol of once every other day for five times) on the sperm motility and viability of asthenozoospermic (AZS) model rats. Related to Discussion. (A-H) Sperm motility including grade A sperm (A), grade A+B sperm (B), straight-line velocity (VSL) (C), curve-line velocity (VCL) (D), average path velocity (VAP) (E), amplitude of lateral head displacement (ALH) (F), linearity (LIN) (G), and straightness (STR) (H). (I) Sperm viability. Note that the sperm motility and viability are improved by 100 Hz-EA treatment compared to mock-EA and ORN-induced AZS model rats. n = 7-8 rats per group. All data are presented as mean ± SEM. $^{*}P < 0.05$, $^{**}P < 0.01$, $^{***}P < 0.001$. One-way ANOVA with Sidak's *post-hoc* test for (A)-(I).

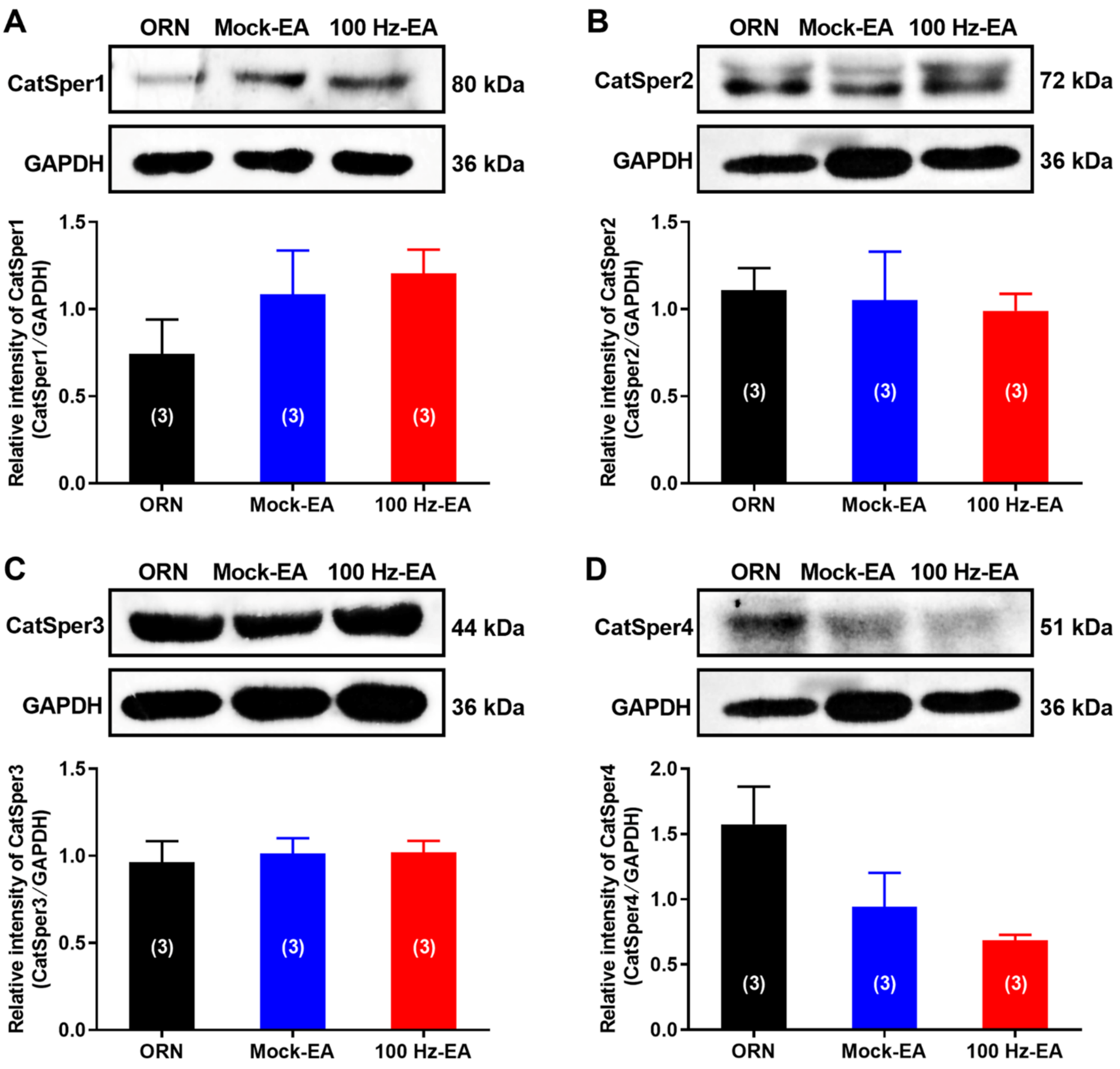

Figure S20. Effects of 100 Hz-EA treatment (by the protocol of once every other day for five times) on CatSper protein (from CatSper1 to CatSper4) expression in the sperm of asthenozoospermic (AZS) model rats. Related to Discussion. (A) Western blot of CatSper1 protein. (B) Western blot of CatSper2 protein. (C) Western blot of CatSper3 protein. (D) Western blot of CatSper4 protein. Note that no significant change in CatSper protein (from CatSper1 to CatSper4) abundance is found in the sperm of AZS rats with 100 Hz-EA treatment compared to mock-EA and ORN-induced AZS rats. n = 3 rats per group. All data are presented as mean ± SEM. P > 0.05, 100 Hz-EA *vs*. mock-EA and ORN, $F_{(2,6)} = 1.43$, $F_{(2,6)} = 0.10$, $F_{(2,6)} = 0.11$, and $F_{(2,6)} = 4.10$ for (A)-(D). One-way ANOVA with Sidak's *post-hoc* test for (A)-(D).

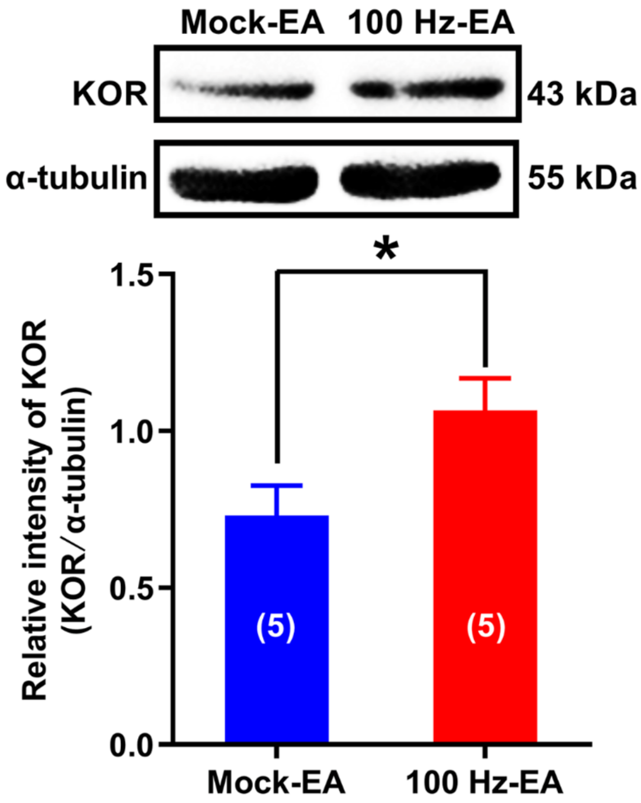

Figure S21. Effects of 100 Hz-EA treatment (by the protocol of once every other day for five times) on *kappa* opioid receptor protein expression in the sperm of asthenozoospermic (AZS) model rats. Related to Discussion. Note that the abundance of *kappa* opioid receptor (KOR) protein is significantly increased in the sperm of AZS rats with 100 Hz-EA treatment compared to mock-EA treatment. n = 5 rats per group. All data are presented as mean ± SEM. $^{*}P < 0.05$. Unpaired *t* test.

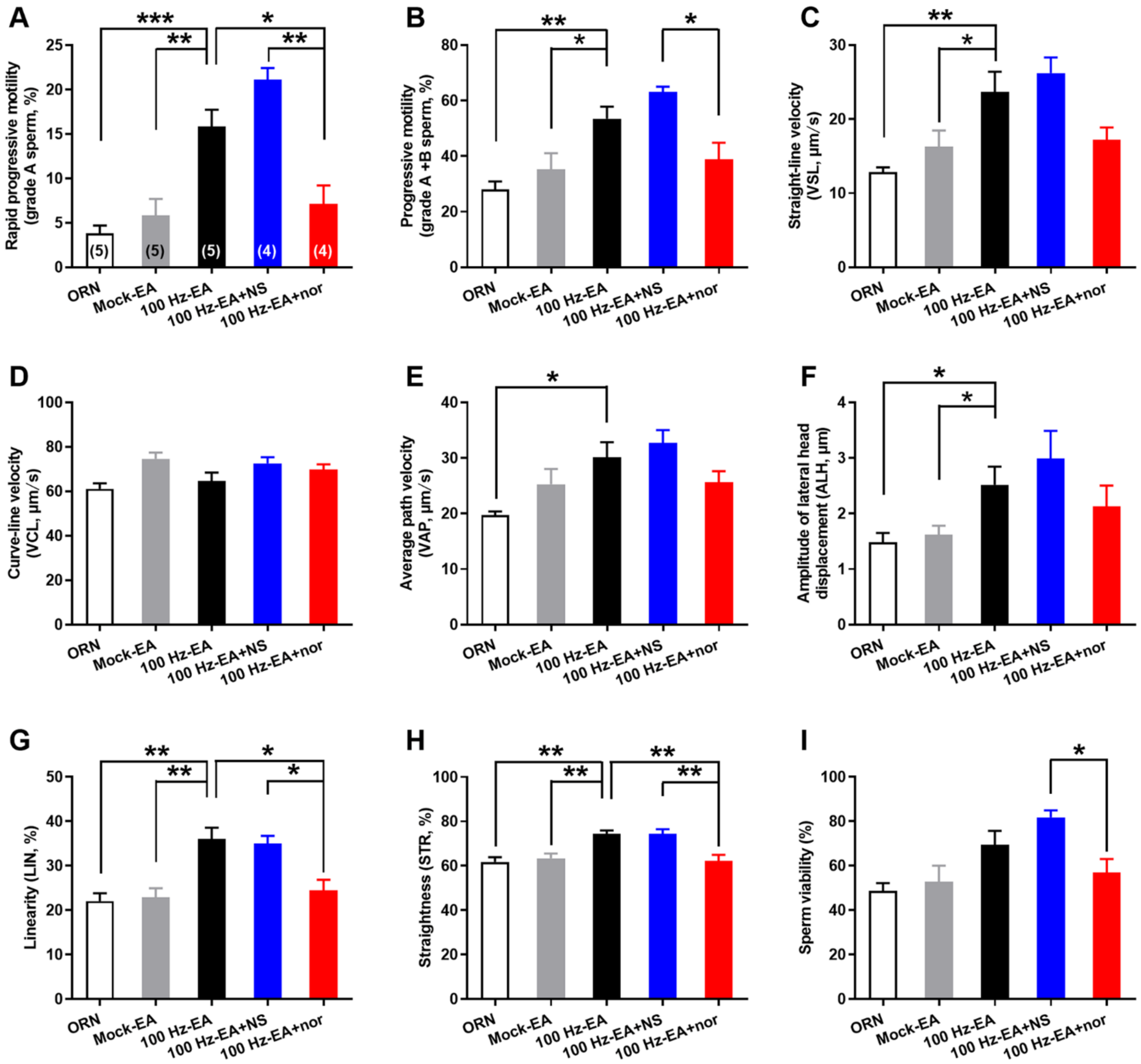

Figure S22. Effects of *kappa* opioid receptor (KOR) antagonist norbinaltorphimine (Nor-BNI) on the improved sperm motility of asthenozoospermic (AZS) model rats with 100 Hz EA treatment. Related to Discussion. (A-H) Sperm motility including grade A sperm (A), grade A+B sperm (B), straight-line velocity (VSL) (C), curve-line velocity (VCL) (D), average path velocity (VAP) (E), amplitude of lateral head displacement (ALH) (F), linearity (LIN) (G), and straightness (STR) (H). (I) Sperm viability. Note that local administration of KOR antagonist Nor-BNI into the testis abolishes the improvement effects of 100 Hz-EA treatment on the sperm motility of AZS rats. ORN: ornidazole, NS: normal saline, nor: nor-BIN. All data are presented as mean ± SEM. n = 4-5 rats per group. $^{*}P < 0.05$, $^{**}P < 0.01$, $^{***}P < 0.001$. One-way ANOVA with Sidak's *post-hoc* test for (A)-(I).

Tables S1-S3 and Legends

Table S1. Key resources table. Related to Material and Methods.

REAGENT or RESOURCE	SOURCE	IDENTIFIER
Antibodies		
Rabbit anti-human CatSper1	Sigma-Aldrich	Cat# SAB1302217
Rabbit anti human/rat CatSper2	Santa Cruz	Cat# sc-98539
Rabbit anti-human CatSper3	Santa Cruz	Cat# sc-98702
Rabbit anti-human CatSper4	Abcam	Cat# ab101892
Rabbit anti-rat CatSper1	Santa Cruz	Cat# sc-33153
Rabbit anti-rat CatSper3	Santa Cruz	Cat# sc-98818
Goat anti-rat CatSper4	Santa Cruz	Cat# sc-83126
Rabbit anti-rat phosphotyrosine	Abcam	Cat# ab179530
Rabbit anti-rat *Kappa* opioid receptor	Abcam	Cat# ab183825
Mouse anti-GAPDH	Santa Cruz	Cat# sc-32233
Mouse anti-α-tubulin	Cell Signaling Technology	Cat# 3873S
Goat anti-rabbit IgG-HRP	Santa Cruz	Cat# sc-2004
Rabbit anti-goat IgG-HRP	Santa Cruz	Cat# sc-2768
Goat anti-mouse IgG-HRP	Santa Cruz	Cat# sc-2005
FITC-labeled goat anti-rabbit IgG	ZSGB-BIO	Cat# ZF-0511
FITC-labeled rabbit anti-goat IgG	ZSGB-BIO	Cat# ZF-0514
Alexa Fluor 647 goat anti-rabbit IgG	YEASEN	Cat# 33113ES60
TRITC-labeled goat anti-rabbit IgG	ZSGB-BIO	Cat# ZF-0316
Bacterial and Virus Strains		
LV-shCatSper1-mCherry	Likeli Technologies	N/A
LV-shCatSper1-ZsGreen	Likeli Technologies	N/A
LV-CatSper1-ZsGreen	Likeli Technologies	N/A
Biological Samples		
Semen sample of male adults	Third Hospital, Peking University, Beijing, China	IRB00001052-13004
Chemicals, Peptides, and Recombinant Proteins		
Ornidazole	Meilun Biotechnology	Cat# MB1173; CAS:16773-42-5
NNC 55-0396	Sigma-Aldrich	Cat# N0287
Percoll	Sigma-Aldrich	Cat# P1644
DMSO	Sigma-Aldrich	Cat# 276855; CAS: 67-68-5
Fura-2 AM	Molecular Probes	Cat# F1201; CAS: 108964-32-5
Pluronic F-127	Molecular Probes	Cat# P3000MP
Fluo-4 AM	Molecular Probes	Cat# F14201; CAS: 273221-67-3
Lectin from Arachis hypogaea FITC conjugate	Sigma-Aldrich	Cat# L7381

nor-Binaltorphimine	Abcam	Cat# ab120078
Critical Commercial Assays		
BCA Protein Assay Kit	Thermo Scientific	Cat# 23227
Fast-staining solution of sperm morphology	Solarbio	Cat# G2572
Experimental Models: Organisms/Strains		
Rat: Sprague-Dawley	Charles River	N/A
Oligonucleotides		
Primers for CatSper1 (human and rat), see Table S2	This paper	N/A
Primers for CatSper2 (human and rat), see Table S2	This paper	N/A
Primers for CatSper3 (human), see Table S2	This paper	N/A
Primers for CatSper4 (human), see Table S2	This paper	N/A
Primers for CatSper3 (rat), see Table S2	This paper	N/A
Primers for CatSper4 (rat), see Table S2	This paper	N/A
Primers for GAPDH (human), see Table S2	This paper	N/A
Primers for β-actin (rat), see Table S2	This paper	N/A
shRNA targeting sequence: CatSper1, see Table S3	This paper	N/A
Recombinant DNA		
Plasmid: rat CatSper1	This paper	N/A
Software and Algorithms		
MetaFluor v7	Molecular Devices	https://www.moleculardevices.com/products/cellular-imaging-systems/acquisition-and-analysis-software/metamorph-microscopy
Leica LAS X 3.0	Leica	https://www.leica-microsystems.com/products/microscope-software
GraphPad Prism 8.0	GraphPad Software	https://www.graphpad.com/scientific-software/

Table S2. PCR primer sequences. Related to Material and Methods.

Genes	Primer	Sequence
CatSper1 (human and rat)	Forward	5'-TTTACCTGTCTCTTCCTCTTCT-3'
	Reverse	5'-ACCAGGTTGAGGAAGATGAAGT-3'
CatSper2 (human and rat)	Forward	5'-GGGTGCTGAGGTCTCTCAAAC-3'
	Reverse	5'-ACCAATGATCCAAGGTGAAGA-3'
CatSper3 (human)	Forward	5'-CCACGGTTGATGGCTGGAC-3'
	Reverse	5'-GTGCATGATCATCACACCCAC-3'
CatSper4 (human)	Forward	5'-TGCCCAAGCATTTCCAGAAC-3'
	Reverse	5'-ACAATGCACCAGTGGCAGCT-3'
CatSper3 (rat)	Forward	5'-TGTTTGGCAATGCGGATAGA-3'
	Reverse	5'-GCAAGCAGGATGAAGAGGAT-3'
CatSper4 (rat)	Forward	5'-CATTCGGGAGATGGCTAACA-3'
	Reverse	5'-AGAGGGATGGGCTCCAGTTT-3'
GAPDH (human)	Forward	5'-ACCACAGTCCATGCCATCAC-3'
	Reverse	5'-TCCACCACCCTGTTGCTGTA-3'
β-actin (rat)	Forward	5'-AGCCATGTACGTAGCCATCC-3'
	Reverse	5'-GCCATCTCTTGCTCGAAGTC-3'

Table S3. CatSper1 shRNA nucleotide sequences. Related to Material and Methods.

Name	Primer	Sequence
shRNA-1	Forward	5’-GCGTGGATCAGACTTCCATGA-3’
	Reverse	5’-TCATGGAAGTCTGATCCACGC-3’
shRNA-2	Forward	5’-GCACCTCTCCTTCTCATATGA-3’
	Reverse	5’-TCATATGAGAAGGAGAGGTGC-3’
shRNA-3	Forward	5’-GCATGTTCCGGATCCTCAAAG-3’
	Reverse	5’-CTTTGAGGATCCGGAACATG-3’

Supplementary Video Legends

Supplementary Video S1. Calcium imaging recorded from the sperm of healthy subjects and idiopathic asthenozoospermic (iAZS) patients, and in the presence or absence of CatSper inhibitor NNC. Related to Fig. 1H. (Video S1-1): Healthy subjects and iAZS patients. $[Ca^{2+}]_i$ fluorescent signals were recorded before and after application of NH_4Cl (30 mM), and the NH_4Cl was added into the HTF^+ medium at 20-30 s post-recording. Note that NH_4Cl induces a significant increase in Fura-2 fluorescent signals ($[Ca^{2+}]_i$ fluorescent signals) in the sperm of healthy subjects, whereas no significant change in $[Ca^{2+}]_i$ fluorescent signals is seen in the sperm of iAZS patients. (Video S1-2): Healthy subjects in the presence or absence of CatSper inhibitor NNC 55-0396 (NNC, 10 μM, diluted in HTF^+ medium). NH_4Cl induces an increase in $[Ca^{2+}]_i$ fluorescent signals in the sperm pretreated with vehicle (HTF^+ medium), however, no obvious change in $[Ca^{2+}]_i$ fluorescent signals is observed in the sperm pretreated with NNC. (Video S1-3): iAZS patients in the presence or absence of NNC. No significant change in NH_4Cl-induced $[Ca^{2+}]_i$ fluorescent signals is observed in the sperm pretreated with either NNC or vehicle.

Supplementary Video S2. Calcium imaging recorded from the sperm of idiopathic asthenozoospermic (iAZS) patients with mock-TEAS or 2 Hz-TEAS treatment. Related to Fig. 2I-2J. (Video S2-1): iAZS patients with mock-TEAS treatment. $[Ca^{2+}]_i$ fluorescent signals were recorded before and after application of NH_4Cl (30 mM), and the NH_4Cl was added into the HTF^+ medium at 20-30 s post-recording. No significant change in NH_4Cl-induced $[Ca^{2+}]_i$ fluorescent signals is observed in the sperm of iAZS patients before and after mock-TEAS treatment. (Video S2-2): iAZS patients with 2 Hz-TEAS treatment. A significant increase in NH_4Cl-induced $[Ca^{2+}]_i$ fluorescent signals is found in the sperm of iAZS patients after TEAS treatment compared to before TEAS treatment. (Video S2-3 and S2-4): iAZS patients with mock-TEAS treatment in the presence or absence of CatSper inhibitor NNC 55-0396 (NNC, 10 μM, diluted in HTF^+ medium). No significant change in NH_4Cl-induced $[Ca^{2+}]_i$ fluorescent signals is seen either in the presence or absence of NNC. (Video S2-5 and S2-6): iAZS patients with 2 Hz-TEAS treatment in the presence or absence of NNC. No significant change in NH_4Cl-induced $[Ca^{2+}]_i$ fluorescent signals is seen in the sperm pretreated with vehicle (HTF^+ medium) or NNC in iAZS patients before TEAS treatment (Video 2-5), whereas after 2 Hz-TEAS treatment, the NH_4Cl-induced $[Ca^{2+}]_i$ fluorescent signals are increased in the sperm pretreated with vehicle but not NNC (Video S2-6).

Supplementary Video S3. Computer-assisted semen analysis (CASA) and calcium imaging for the epididymal sperm of asthenozoospermic (AZS) model rats. Related to Fig. 3 and Figure S2E-2J. (Video S3-1, related to Fig. 3A and Figure S2E-2J): CASA for the sperm motility of naïve rats, vehicle (CMC-Na solution)-treated rats, and ornidazole (ORN)-induced AZS rats. Note that the sperm motility is significantly decreased in the epididymal sperm of AZS rats compared to naïve and vehicle control rats. (Video S3-2, related to Fig. 3C-3D): Calcium imaging recorded from the epididymal sperm of AZS and control rats. $[Ca^{2+}]_i$ fluorescent signals were recorded before and after application of NH_4Cl (30 mM), and the NH_4Cl was added into the HS^+ medium at 20-30 s post-recording. Note that NH_4Cl induces a significant increase in $[Ca^{2+}]_i$ fluorescent signals in the sperm of vehicle (CMC-Na solution)-treated rats

but not AZS rats. (Video S3-3, related to Fig. 3E): Calcium imaging recorded from the epididymal sperm of vehicle (CMC-Na solution)-treated rats in the presence or absence of CatSper inhibitor NNC 55-0396 (NNC, 10 μM, diluted in HS^+ medium). NH_4Cl induces a significant increase in $[Ca^{2+}]_i$ fluorescent signals in the absence but not presence of NNC. (Video S3-4, related to Fig. 3F): Calcium imaging recorded from the epididymal sperm of AZS rats in the presence or absence of CatSper inhibitor NNC. No significant change in NH_4Cl-induced $[Ca^{2+}]_i$ fluorescent signals is seen in the epididymal sperm of AZS rats either in the presence or absence of NNC.

Supplementary Video S4. Computer-assisted semen analysis (CASA) and calcium imaging for the epididymal sperm of CatSper1 knockdown (KD) rats. Related to Fig. 4 and Figure S6E-6J. (Video S4-1, related to Fig. 4D-E, and Figure S6E-J): CASA for the sperm motility of LV-shCatSper1-treated KD rats and LV-ZsGreen-treated control rats. Note that the sperm motility is significantly decreased in the epididymal sperm of KD rats compared to control rats. (Video S4-2, related to Fig. 4F): Calcium imaging recorded from the epididymal sperm of KD and control rats. $[Ca^{2+}]_i$ fluorescent signals were recorded before and after application of NH_4Cl (30 mM), and the NH_4Cl was added into the HS^+ medium at 20-30 s post-recording. Note that NH_4Cl induces a significant increase in $[Ca^{2+}]_i$ fluorescent signals in the sperm of control rats but not KD rats. (Video S4-3, related to Fig. 4F): Calcium imaging recorded from the epididymal sperm of LV-ZsGreen-treated control rats in the presence or absence of CatSper inhibitor NNC 55-0396 (NNC, 10 μM, diluted in HS^+ medium). NH_4Cl induces a significant increase in $[Ca^{2+}]_i$ fluorescent signals in the absence but not presence of NNC. (Video S4-4, related to Fig. 4F): Calcium imaging recorded from the epididymal sperm of KD rats in the presence or absence of CatSper inhibitor NNC. No significant change in NH_4Cl-induced $[Ca^{2+}]_i$ fluorescent signals is observed in the epididymal sperm of KD rats either in the presence or absence of NNC.

Supplementary Video S5. Computer-assisted semen analysis (CASA) and calcium imaging for the epididymal sperm of asthenozoospermic (AZS) rats with 2 Hz-EA or mock-EA treatment. Related to Fig. 6 and Figure S13. (Video S5-1, related to Fig. 6A and Figure S13): CASA for the sperm motility of AZS rats with 2 Hz-EA or mock-EA treatment. Note that the sperm motility is increased in the epididymal sperm of AZS rats treated with 2 Hz-EA compared to mock-EA. (Video S5-2, related to Fig. 6C): Calcium imaging recorded from the epididymal sperm of AZS rats with 2 Hz-EA or mock-EA treatment. $[Ca^{2+}]_i$ fluorescent signals were recorded before and after application of NH_4Cl (30 mM), and the NH_4Cl was added into the HS^+ medium at 20-30 s post-recording. Note that NH_4Cl induces a significant increase in $[Ca^{2+}]_i$ fluorescent signals in the sperm of AZS rats treated with 2 Hz-EA but not mock-EA. (Video S5-3, related to Fig. 6C): Calcium imaging recorded from the epididymal sperm of mock EA-treated AZS rats in the presence or absence of CatSper inhibitor NNC 55-0396 (NNC, 10 μM, diluted in HS^+ medium). No significant change in NH_4Cl-induced $[Ca^{2+}]_i$ fluorescent signals is seen in the epididymal sperm of mock EA-treated AZS rats either in the presence or absence of NNC. (Video S5-4, related to Fig. 6C): Calcium imaging recorded from the epididymal sperm of 2 Hz EA-treated AZS rats in the presence or absence of NNC. NH_4Cl induces a significant increase in $[Ca^{2+}]_i$ fluorescent signals in the absence but not presence of NNC.

Supplementary Video S6. Computer-assisted semen analysis (CASA) and calcium imaging for the epididymal sperm of 2 Hz EA-treated AZS rats with CatSper1 knockdown (KD). Related to Fig. 7 and Figure S16E-J. (Video S6-1, related to Fig. 7C-D and Figure S16E-J): CASA for the sperm motility of 2 Hz EA-treated AZS rats with CatSper1 knockdown (LV-shCatSper1) or control vector (LV-ZsGreen). Note that the sperm motility is decreased in the epididymal sperm of 2 Hz EA-treated AZS rats with CatSper1 knockdown (LV-shCatSper1) compared to control vector (LV-ZsGreen). (Video S6-2, related to Fig. 7E): Calcium imaging recorded from the epididymal sperm of 2 Hz EA-treated AZS rats with CatSper1 knockdown (LV-shCatSper1) or control vector (LV-ZsGreen). $[Ca^{2+}]_i$ fluorescent signals were recorded before and after application of NH_4Cl (30 mM), and the NH_4Cl was added into the HS^+ medium at 20-30 s post-recording. Note that NH_4Cl induces a significant increase in $[Ca^{2+}]_i$ fluorescent signals in the sperm of 2 Hz EA-treated AZS rats with control vector (LV-ZsGreen) but not LV-shCatSper1 treatment. (Video S6-3, related to Fig. 7E): Calcium imaging recorded from the epididymal sperm of 2 Hz EA-treated AZS rats with control vector (LV-ZsGreen) treatment in the presence or absence of CatSper inhibitor NNC 55-0396 (NNC, 10 μM, diluted in HS^+ medium). NH_4Cl induces a significant increase in $[Ca^{2+}]_i$ fluorescent signals in the absence but not presence of NNC. (Video S6-4, related to Fig. 7E): Calcium imaging recorded from the epididymal sperm of 2 Hz EA-treated AZS rats with LV-shCatSper1 treatment (KD) in the presence or absence of NNC. No significant change in NH_4Cl-induced $[Ca^{2+}]_i$ fluorescent signals is seen in the epididymal sperm of 2 Hz EA-treated AZS rats with CatSper1 knockdown (LV-shCatSper1) either in the presence or absence of NNC.
